# Get Checked… Where? The Development of a Comprehensive, Integrated Internet-Based Testing Program for Sexually Transmitted and Blood-Borne Infections in British Columbia, Canada

**DOI:** 10.2196/resprot.6293

**Published:** 2016-09-20

**Authors:** Mark Gilbert, Devon Haag, Travis Salway Hottes, Mark Bondyra, Elizabeth Elliot, Cathy Chabot, Janine Farrell, Amanda Bonnell, Shannon Kopp, John Andruschak, Jean Shoveller, Gina Ogilvie

**Affiliations:** ^1^ British Columbia Centre for Disease Control Vancouver, BC Canada; ^2^ School of Population and Public Health University of British Columbia Vancouver, BC Canada; ^3^ College of Registered Nurses of British Columbia Vancouver, BC Canada; ^4^ Provincial Health Services Authority Vancouver, BC Canada; ^5^ British Columbia Centre for Excellence in HIV/AIDS Vancouver, BC Canada; ^6^ Children and Women’s Health Centre of British Columbia Vancouver, BC Canada

**Keywords:** Internet, sexually transmitted diseases, testing, health care delivery, health services research, intervention studies

## Abstract

**Background:**

Testing for sexually transmitted and blood-borne infections (STBBI) is an effective public health strategy that can promote personal control of one’s health and prevent the spread of these infections. Multiple barriers deter access to testing including fear of stigmatization, inaccurate health care provider perceptions of risk, and reduced availability of clinic services and infrastructure. Concurrent increases in sexually transmitted infection (STI) rates and demands on existing clinical services make this an even more pressing concern. Web-based testing offers several advantages that may alleviate existing clinical pressures and facilitate appropriate testing access.

**Objective:**

This paper describes the planning, development, and usability testing of a novel Web-based testing service, GetCheckedOnline (GCO), as a complementary testing option integrated within existing sexual health services within British Columbia (BC).

**Methods:**

From 2009 to 2014, we engaged a multidisciplinary team in the design and development of GCO. We conducted 3 initial research studies to ascertain the opinions of youth, men who have sex with men (MSM), and STI clinic clients regarding Web-based testing and elicited perspectives of sexual health care providers through focus groups. We developed an informed consent process, risk assessment questions, and test recommendations based on provincial and national guidelines and evaluated these through consultations with clinical and community stakeholders. We also conducted a preliminary health equity impact assessment whose findings also informed the GCO program mode. Finally, from April 2011 to December 2012 we gathered qualitative data from 25 participants on the functionality and usability of a GCO prototype and incorporated their recommendations into a final model.

**Results:**

GCO launched in the fall of 2014 across 6 pilot sites in Vancouver, BC. The service involves 3 main steps: (1) create an account, complete an assessment, and print a laboratory requisition, (2) provide blood and urine specimens at participating laboratory locations, and (3) receive test results on the Internet or by phone. During this pilot phase, we promoted GCO to existing STI clinic clients and MSM in the Greater Vancouver region. A rigorous mixed-method evaluation of GCO’s uptake, acceptability, and health system impacts is currently underway.

**Conclusions:**

GCO is the first comprehensive Web-based STBBI testing program in Canada that is integrated with existing sexual health services, with the potential to reduce pressures on existing clinical services and reach populations facing the greatest barriers to testing. Our experience highlights the facilitators and challenges of developing and implementing novel complex eHealth interventions within the health care system, and underscores the importance of considering broader implementation contexts.

## Introduction

In 1999, an outbreak of syphilis among gay, bisexual, and other men who have sex with men (MSM) in San Francisco who were users of online chat room heralded the beginning of a “new era” for prevention of sexually transmitted infections (STI) [1]. The recognition that the Internet posed both a new risk environment for STI and offered unique opportunities to reach populations affected by STI has since led to the development of a wide array of Web-based interventions, including partner notification programs, tailored educational interventions, and Web-based outreach [2,3]. Around the same time in British Columbia (BC) as elsewhere, community surveys and STI clinic records demonstrated increasing use of the Internet to find sex partners by MSM and other populations at higher risk of infection [4]. As a result of these behavioral and intervention paradigm shifts, the British Columbia Centre for Disease Control (BCCDC) prioritized the development of Web-based sexual health services, starting in 2004 with the first nursing-led cyber-outreach program in Canada [5]. Following further consultation with international experts in this nascent field and assessment of local service gaps, development of a Web-based testing program for sexually transmitted and blood-borne infections (STBBI) was prioritized in 2008.

While promoting testing is a longstanding cornerstone of public health strategies for control of STBBI, multilevel barriers to appropriate testing persist in BC as in most jurisdictions. These include individual (eg, risk perception, privacy concerns, fear of disclosing sexual behavior, knowledge of testing locations, discomfort with health care professionals), provider (eg, discomfort with questions about sexual orientation, inaccurate perceptions of risk), and clinic barriers (eg, travel requirements, limited clinic hours, wait times), and these can be particularly pronounced in rural areas [6-9]. BC is also similar to other jurisdictions by having increasing STI rates and demands on STI clinical services that coincide with overall reductions in clinical capacity and infrastructure [10,11]. Web-based testing services may overcome some of these barriers and demands, as these services promote a patient-centered approach and allow clients to access testing without presenting to a provider or clinic.

Typically, Web-based testing involves visiting a website to request a home self-collection kit or to print a laboratory form to take to a laboratory to provide specimens, with results being provided through a website, text messaging, or by phone. Web-based testing programs take a variety of forms, the most common being population-based screening for a single infection (usually chlamydia by self-sampling) [12,13]. Comprehensive models testing for multiple STBBI are less common [14,15]. Programs can be stand-alone programs or be fully integrated with clinical services [15-19]. Evidence of acceptability, reach, and satisfaction with these programs is robust, confirming that Web-based STBBI testing is in high demand, particularly among youth and MSM. Some programs have demonstrated uptake of Web-based testing in high-risk or target groups and found high positivity rates, including clients from diverse socioeconomic positions and races, clients with a history of STI diagnosis, and clients with behavioral markers of STI risk [20-27]. A small number of studies have suggested Web-based testing is cost effective [25,28-30].

Recognizing the potential of Web-based testing to reduce inequities in access to testing among populations with higher infection rates, in 2009 the Provincial Health Services Authority (PHSA) awarded the BCCDC (an agency within the PHSA) 5 years of funding to develop an Online Sexual Health Services Program. This included the development of an Internet-based testing program with 3 objectives: (1) to improve sexual health by increasing the uptake and frequency of STBBI testing and earlier diagnosis, (2) to reach populations with a greater prevalence of infection and barriers to access testing, and (3) to increase the capacity of STI clinic services and improve the use of clinician resources. MSM, youth younger than 25 years, and people living in rural areas were named as initial priority populations for this service, given high infection rates and Web-based sex-seeking behaviors [11,31], barriers to accessing confidential, culturally sensitive and appropriate care [6,8], and demonstrated acceptance of Web-based sexual health interventions [32-35]. In this article, we will describe the development of this Web-based testing service for STBBI – branded GetCheckedOnline (GCO) – and plans for its evaluation. In so doing, we hope that we will also provide helpful insights for other researchers and service providers interested in developing Web-based sexual health services within complex health care systems.

## Methods

### Theoretical Framework

Our approach to developing GCO involved the same steps and activities typically recommended for the development of eHealth interventions. These included: using a multidisciplinary approach (with involvement of different research and provider disciplines on the development team), involvement of stakeholders including potential users throughout the process, conducting continuous and systematic evaluation throughout all phases of development, and use of robust, mixed-methods for formative and summative evaluation [36]. Our primary assumption and rationale was that GCO would reduce barriers to STBBI testing among individuals who are already motivated to test (and was not to change testing behavior among unmotivated individuals per se). As our focus was on adoption of GCO, we used elements of diffusion of innovation theory to inform the development and evaluation of GCO; for example, by considering the relative advantages and disadvantages of GCO from the perspective of potential users, identifying the characteristics of potential adopters, and considering the health system contexts in which GCO is implemented [37,38]. Finally, an important theoretical underpinning of GCO was our positioning of the intervention as being complementary to (not replacing), and fully integrated with, existing public health and clinic-based sexual health services in BC.

### Planning Phase: 2009-2011

#### Establishing a Multidisciplinary Team

The development of GCO was led by the BCCDC Online Sexual Health Services (OSHS) program, which consisted of a medical lead, program manager, business analyst, and epidemiologist. At the outset, a program of research was established between the OSHS and the Youth Sexual Health Team, a research unit at the University of British Columbia, based on an integrated knowledge translation model (ie, where knowledge translation principles are applied to the entire research process, with involvement of knowledge users as equal partners) [39], and drawing on a range of qualitative and quantitative research disciplines including public health and clinical research, epidemiology, and social sciences.

#### Identifying, Engaging, and Consulting With Stakeholders

We identified 3 groups of stakeholders to engage in the development of GCO either on an ad hoc or continuous basis: internal stakeholders within the BCCDC; stakeholders within other PHSA agencies (eg, public health laboratory, privacy, information technology); and external stakeholders (Table 1).

We conducted a stakeholder analysis and developed a communication strategy for engaging stakeholders, including the development of fact sheets and standard presentations. Initially, we met with each stakeholder to provide an orientation to the concept of Web-based STBBI testing and GCO, and to discuss the nature of their involvement with GCO development. We provided ongoing updates about GCO development through a team blog and email bulletins, as well as seeking out opportunities to disseminate information about GCO through community agency networks, publications, and events [40-42]. We then established 3 stakeholder committees to guide the activities of, and provide updates about, GCO development:

1. Clinical Integration Committee (CIC): An internal decision-making group that met monthly to determine how GCO should be integrated with the provincial STI clinic at BCCDC, which would be responsible for clinical and public health follow-up of test results. The CIC was comprised of medical and nursing leads for the clinic, education and outreach programs at BCCDC.

2. Community Consultation Working Group (CCWG): An external advisory group that met biannually to ensure GCO would be a useful resource to community organizations and tailored to meet the needs of the populations they served. The CCWG was comprised of community organizations working in sexual health and STBBI prevention (including with youth and MSM).

3. Internet Services Committee (ISC): An advisory group that met monthly to receive updates about GCO and to guide its development, comprised of BCCDC, PHSA, and external stakeholders.

#### Reviewing the Literature and Expert Consultation

We reviewed published and gray literature to identify Web-based STBBI testing programs and evaluations of their impact, and to summarize barriers and facilitators of STBBI testing that are potentially mediated through Web-based testing. We then contacted program experts involved with comprehensive Web-based testing programs integrated with clinical services in San Francisco and Amsterdam, to learn about their experience with setting up these programs [14,15].

**Figure 1:**
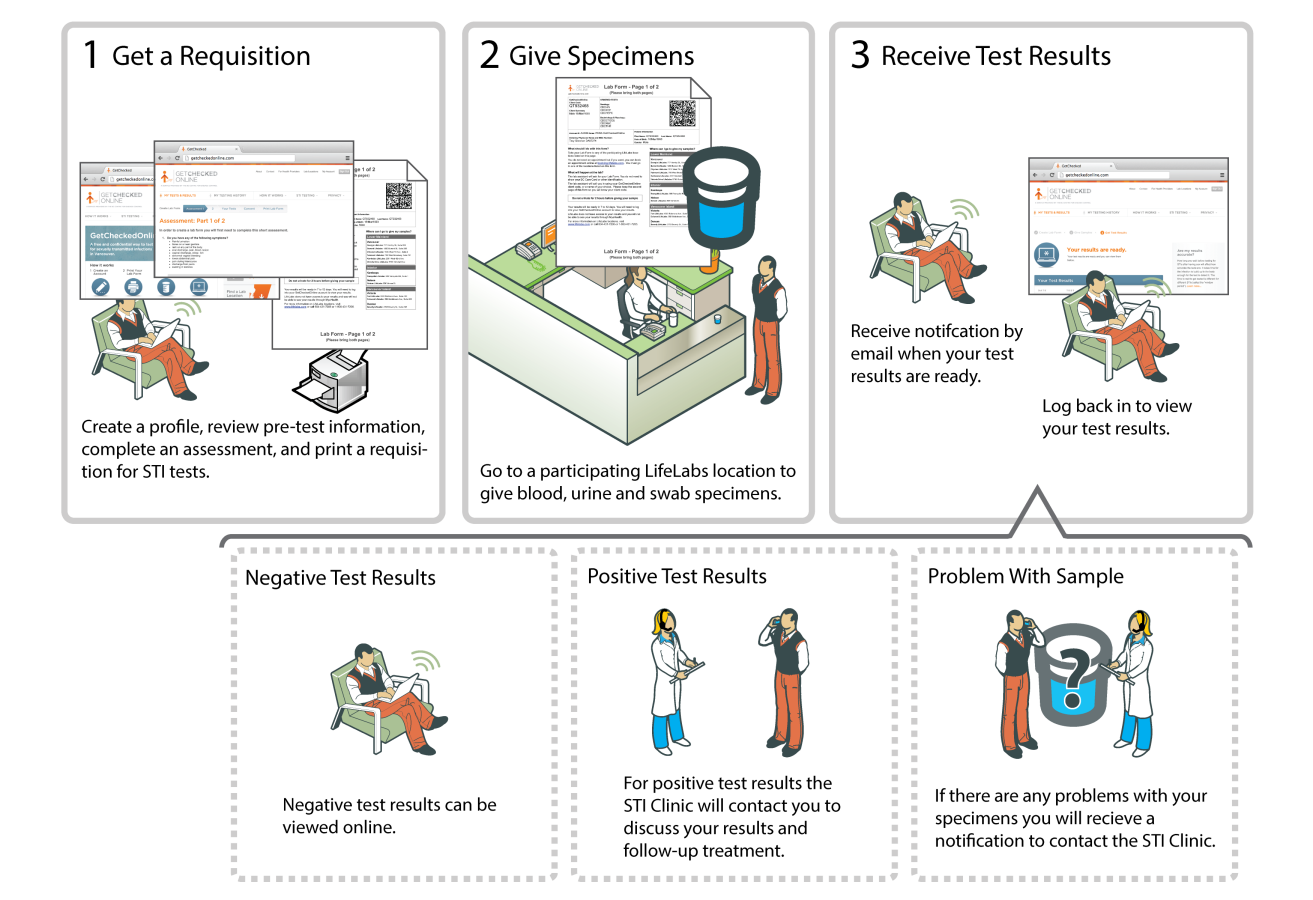
High level overview of GetCheckedOnline used during formative research.

Based on this information and consultation, we developed a high level model for GCO to use in future consultations. This model described the program’s 3 main steps: create an account, complete a risk assessment, and print a laboratory requisition; provide blood and urine specimens (only; no oral, vaginal, or rectal swabs); receive test results (with paths described if negative, positive, or if there is a problem with the result) (Figure 1). This model did not include mailing of home test kits for human immunodeficiency virus (HIV) as these are not yet licensed for use in Canada. We also did not propose including self-collection of specimens at home as sending collected specimens potentially containing infectious agents by general mail is not permitted in Canada. In this high level model, positive results are delivered by a health care provider by phone, which is consistent with current practice at the provincial STI clinic in order to provide appropriate posttest counseling and ensure treatment and appropriate follow-up. In contrast, all negative test results would be viewed on the Internet, as is the case for many Web-based testing programs in order to reduce barriers to accessing test results (ie, by eliminating requirements to contact the clinic).

**Table 1:** Key stakeholders involved in the development of GetCheckedOnline.

Stakeholder		Role in relation to GCO^a^	Involvement with GCO development
**BCCDC^b^**			
	Provincial sexually transmitted infection clinic	Responsible for clinical aspects of GCO implementation (authority for ordering tests, review and management of results, entering results into app)	Continuous; part of ISC, CIC, GWG^c^; key knowledge user involved in research activities
	Education and outreach programs	Provide support for or lead other Web-based/clinic-based sexual health services with which GCO is integrated	Continuous; part of ISC
	Communications	Provide support for media and public communications	Continuous; part of ISC
	Executive	Responsible for overall strategic direction and operations of BCCDC including the Division responsible for GCO	Ad hoc; key knowledge user involved in research activities
**PHSA^d^ (government agency within which BCCDC is located)**			
	BC Public Health Laboratory	Responsible for conducting all tests ordered through GCO	Continuous; part of ISC, GWG; key knowledge user involved in research activities
	Privacy	Provides privacy-related advice on GCO	Continuous; part of ISC
	Risk management and legal	Provides legal advice regarding risk management of GCO	Ad hoc
	Information management/IMITS^e^	Responsible for approving the technical specifications and final application	Continuous; part of TWC^f^
	Executive	Responsible for overall strategic direction and administration of PHSA (including BCCDC, BC Public Health Laboratory, privacy, risk management, and IMITS	Ad hoc; key knowledge user involved in research activities
**External stakeholders**			
	Health care providers conducting STBBI^g^	Interact with users of GCO (eg, refer clients to GCO)	Consulted during planning phase
	Community organizations working with youth or men who have sex with men, and/or in sexual health	Interact with users of GCO (eg, refer clients to GCO). Promotion of GCO as part of education or outreach programs to clients.	Continuous; part of ICS, CIC^c^, CCWG^h^; key knowledge user involved in research activities
	LifeLabs	Private laboratory company that operates the specimen collection sites for GCO	Consulted during development, testing, and implementation planning
	Public Health programs in the other 6 regional or provincial health authorities in BC	Oversee regional public health testing initiatives with which GCO must be aligned	Ad hoc
	Ministry of Health	Sets provincial strategies for STBBI testing and oversight for provincial testing initiatives with which GCO must be aligned	Continuous; part of ISC; key knowledge user involved in research activities
	Professional practice regulatory bodies (College of Physicians and Surgeons of BC; College of Registered Nurses of BC)	Determines acceptable scope of practice for physicians and registered nurses involved with GCO	Ad hoc

^a^GetCheckedOnline.

^b^British Columbia Centre for Disease Control.

^c^Internet Services Committee (ISC); Clinical Integration Committee (CIC); GetCheckedOnline Working Group (GWG).

^d^Provincial Health Services Authority (PHSA).

^e^Information technology services (IMTS).

^f^Technical working group (TWC).

^g^Sexually transmitted and blood-borne infections testing (STBBI).

^h^Community consultation working group (CCWG).

#### Consulting With Potential Users

Given the importance of end-user input at early stages in the development of novel eHealth interventions, we conducted 3 research studies to ascertain the opinions of youth, MSM, and STI clinic clients about Web-based sexual health services and our Web-based testing model [32,34,43]. Overall, GCO was perceived as private, convenient, and providing greater control over testing. We elicited a number of concerns, including privacy of data and security of the app, reliance on outdated technologies (eg, printing), and anxiety at receiving a positive result on the Internet. In these studies, the participants proposed mitigation strategies for these concerns, many of which were incorporated into the GCO model (Table 2).

#### Determining Regulatory and Practice Requirements

As a new testing paradigm, Web-based testing raised a number of questions regarding its relation to existing regulatory and practice requirements for physicians and nurses in BC (eg, can a physician order tests recommended through a Web-based app, and if so how, and is any liability assumed?). To answer these questions, we consulted with provincial practice regulatory bodies for physicians and nurses, and the national medical protection insurance agency. We established that GCO’s model, where a BCCDC STI specialist physician is the ordering physician with results reported to and followed up by provincial STI certified registered nurses and managed according to standard protocols, is an extension of acceptable clinical practice. We also developed policies to address identified clinical nursing practice gaps, including guidelines for providing nursing services on the Internet and for emailing clients.

**Table 2:** Key findings from potential users on the acceptability and perceptions of Web-based sexual health services/testing and how these influenced the design of GetCheckedOnline.

Activity	Key findings	Influence on GCO^a^ design
Interviews and focus groups with youth to determine their perceptions of sexual health websites [32]	For sexual health–related websites youth preferred practical information, professional approaches to design and content (vs colloquial or explicit language or images)	Adopted professional tone using every day, noncolloquial language and select use of imagery
Interviews and focus groups with youth, MSM^b^, and clinic clients to determine their perceptions of Web-based testing in general and GCO specifically [43]	Web-based testing perceived as convenient, offering immediate access to testing, greater privacy, reduced anxiety compared with face-to-face testing, and greater control over the testing process Concerns about providing personal information via the Internet, potential for abuse (eg, if an account was created using email belong to someone else), distrust of security of data provided via the Internet, lack of comprehensive pretest information, lack of support for individuals receiving a positive result. Expectations that Web-based testing would be professional, adhere to standard guidelines (and advise when different, such as lack of swabs), be fully on the Internet (eg, from booking appointments for specimen collection, to electronic ordering of tests, to getting results and prescriptions), ability to control how and when they receive notifications	Minimum data is collected with rationale for questions provided Account creation requires email validation Explicit privacy policy and terms of use developed to explain how data is collected, stored, and used Advice provided for additional privacy measures (eg, clearing cache) Detailed pretest information provided No positive results provided via the Internet, only by phone, with links to other services accessible throughout the website Testing reminders can be turned off Tailored educational information is provided for other sexually transmitted and blood-borne infections testing or prevention strategies not available through GCO (eg, emergency contraception, throat, and rectal swabs) GCO clearly identified as a program of the British Columbia Centre for Disease Control Wording on the GCO website and promotions emphasize privacy and convenience of the service
Web-based national survey of Canadian MSM to determine intention to use Web-based testing [34]	Overall intention to use Web-based testing was 72%, with little variation by participant characteristics. Greatest perceived benefits were privacy, convenience, and testing any time. Greatest drawbacks were inability to see a doctor or nurse, wanting to talk to someone about results, not wanting Web-based results, and low trust in the service	

^a^GCO: GetCheckedOnline.

^b^MSM: men who have sex with men.

#### Consulting With Sexual Health Care Providers

As GCO is implemented in an existing sexual health care system where Web-based testing clients may also be accessing sexual health services at other clinics not operated by BCCDC, we conducted focus groups with local sexual health care providers to understand their opinions of GCO [44]. Providers perceived GCO as an inevitable evolution within the current system of care, with perceived benefits including shifting the locus of control from providers to patients, addressing testing barriers (eg, privacy concerns, clinic hours of operation), facilitating increased engagement in sexual health care (eg, including reminders for pap testing), and freeing up provider time and ability to see more complex patients. Providers also considered that these benefits may be offset by perpetuating existing inequities in populations GCO is trying to reach (eg, youth who do not have access to a private printer, MSM requiring swabs for diagnosis of oral or rectal STI) or predominantly being used by individuals who already have the resources necessary to access testing (eg, tech “savvy,” higher income). A number of potential personal or clinical harms were identified, such as anxiety at receipt of result notifications, repeated use by individuals at lower risk (“worried well”), misunderstanding test limitations, such as window periods, inadequate pre- and posttest counseling, and missed opportunities for education and prevention such as contraception. As with potential users, mitigation strategies for these harms were also proposed and incorporated into GCO (Table 3). At the same time, providers recognized that many of these harms could also occur within face-to-face clinical testing encounters.

**Table 3:** Potential harms and mitigation strategies recommended by sexual health care providers, and how these were addressed in the design of GetCheckedOnline.

Potential harm	Recommended mitigation strategy	How addressed
Anxiety related to viewing email notification or retrieving voicemail (if positive) outside of clinic hours	Provide after-hours support, send notifications early in the day Notification emails should be generic and not include results	Links to BCCDC^a^ sexual health website and provincial after-hours support services Generic wording used for notification emails
Not addressing underlying anxiety of repeat tests by the “worried well”	Ability to monitor and intervene if appropriate (eg, refer to clinic for care)	Monitored during the pilot evaluation Clinic protocol developed to handle this scenario
Misunderstanding information on the website, such as window periods, symptoms	Ensure appropriate educational content on website related to test limitations and symptoms	Information accessible throughout the site related to test limitations and window periods Links to British Columbia Centre for Disease Control sexual health website for more information about symptoms
Inadequate pre- and posttest counseling	Provide equivalent information on website, with some mandatory information Include clear consent process and disclaimer regarding limitations of Web-based testing	Content from provincial pre/posttest guidelines incorporated, with mandatory and optional content Consent page including acknowledgement of limitations as final step before printing requisition
Missed opportunities for education and prevention that can be elicited during clinical testing encounters	Include information and referrals for pap testing, human papilloma virus vaccine	Tailored recommendations for sexually transmitted and blood-borne infections prevention provided based on assessment responses, including vaccines, oral and rectal swabs, emergency contraception, HIV postexposure prophylaxis
Does not include all potentially relevant tests (eg, Hepatitis C, swabs)	Include Hepatitis C testing Have clear referrals to clinics for other tests On assessment include question about specific sexual acts (oral, vaginal, anal) and recommend swabs if appropriate Explain why certain tests are not offered	Hepatitis C testing included for men who have sex with men, or history of injection drug use Swabs prioritized for inclusion after implementation
Not answering assessment questions accurately and inappropriate tests recommended (or not)	Give option to skip assessment and recommend all tests Encourage clients to provide accurate information (through disclaimer, encourage to select “prefer not to answer” option)	Importance of providing accurate information emphasized Clients have option of deselecting any recommended tests
Positive results not followed up because of client providing fake contact information	Encourage use of real name and phone number	Importance of using real name or consistent pseudonym, and providing telephone number emphasized

^a^BCCDC: British Columbia Centre for Disease Control.

### Development, Usability Testing, and Revision: 2011-2014

**Figure 2:**
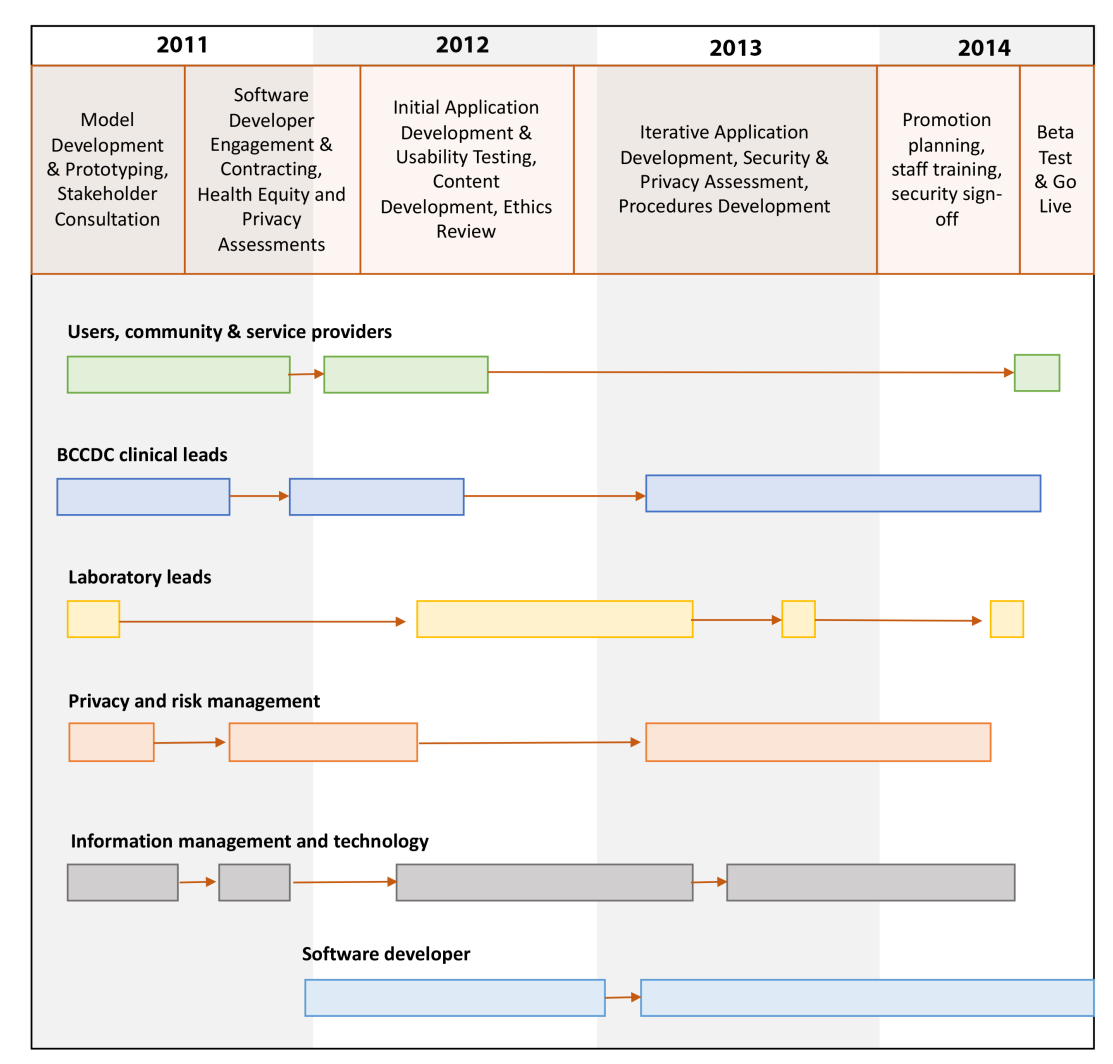
Key players involved in the development, testing, and revision of the GetCheckedOnline app.

Stakeholder and end user consultation continued throughout the development, usability testing, and revision stages (details shown in Figure 2).

We established a GCO Working Group including the core GCO development team and representatives from the BCCDC clinic (nursing and clerical) and the PHSA Public Health Laboratory (which conducts the testing for GCO specimens). The working group met approximately every 2 weeks to develop the detailed requirements (ie, blueprint) for the GCO app and the final GCO service. In addition, we established a GCO Technical Working Group, comprised of the GCO development team and representatives from different program areas in Information Technology (IT) (eg, databases, servers, network security). The group met every 2 weeks to develop the architecture, hosting, and other technical requirements for the app. The final model was informed by findings from the planning phase as well as the additional activities below.

#### Assessing GetCheckedOnline’s Impact on Health Equity

The “techno-optimism” with which Web-based health interventions are viewed is tempered by the reality that their adoption is patterned along social gradients (eg, digital divides) [45,46]. New testing technologies such as GCO may reinforce or reproduce the relationship between social position and health status if only taken up by individuals who already have the resources needed to access clinic-based STBBI testing (eg, social capital, education) [47]. We conducted a preliminary health equity impact assessment (HEIA; screening, scoping) consisting of a literature review and expert consultations. In so doing, we identified ways in which GCO was likely to reinforce or circumvent health inequities in sexual health for historically underserved and marginalized populations with a higher burden of STBBI (including: youth; MSM; people from ethnocultural minorities; intersex, transgender or gender variant populations; Indigenous people; residents of rural areas) [48]. HEIA recommendations that were incorporated into GCO design included: collecting information on ethnicity and gender identity; avoiding normative and stigmatizing language and images; expanding testing options to include hepatitis C.

#### Meeting Requirements for Pretest Counseling and Informed Consent

As flagged during consultations with end-users and sexual health care providers, we needed to determine how provider-delivered pretest counseling and the obtaining of informed consent could be translated to a Web-based app. We did so by: (1) reviewing the published literature to determine the effectiveness of alternate models of providing pretest counseling (eg, videos, written information), (2) reviewing national and provincial testing policies and procedures, and (3) consulting with a clinical ethicist, privacy advisor, legal counsel, and provincial nursing practice leads to determine the necessary app requirements in order to obtain informed consent on the Internet (eg, mandatory vs optional steps). We included a specific consent step on GCO that must be completed by users prior to printing a laboratory test requisition, and conducted individual interviews with end-users following their participation in usability testing of the GCO app to probe specifically about their perceptions of the consent webpage (which overall were favorable) [49].

#### Determining Risk Assessment Questions and Test Recommendations

As GCO was conceptualized as an extension of clinical STBBI testing services offered by BCCDC, we intended the assessment step and testing recommendations to mirror routine clinical practice as much as possible. We reviewed national and provincial guidelines for STBBI testing, treatment and scope of practice, and the BC epidemiology of STBBI in our target populations. To identify assessment questions that could be used for recommending specific tests and tailored educational messages, we reviewed existing Web-based risk assessment tools and the published literature to identify models or variables that were predictive of STI infection [50,51].

Our final set of assessment questions, testing recommendations, and tailored messages were evaluated through usability testing and consultation with clinical and community stakeholders. These included routine recommendation for all clients of urine testing for chlamydia and gonorrhea, and serum testing for HIV and syphilis (as these are generally routinely recommended for BCCDC STI clinic clients and MSM); serum testing for hepatitis C was also recommended for MSM (optional) and individuals with a history of injection drug use. Clients can opt out (deselect) any of the recommended tests. Assessment questions were also designed to (1) provide tailored recommendations for additional testing or prevention interventions that may be indicated (including HIV postexposure prophylaxis, emergency contraception, and need for oral and/or rectal swabs for chlamydia and gonorrhea testing), and (2) to determine the recommended frequency of testing in order to set up testing reminders that are sent by email to GCO clients (ie, 3, 6, or 12 months).

#### Usability Testing and Revision

As the incorporation of user feedback is a key component of app development, we conducted testing of the website with potential end-users at 3 separate points during the development process. All usability testing was done in-person with participants recruited through Web-based advertising and from attendees of the Provincial STI Clinic at BCCDC. The first round of testing, performed on a prototype with 10 participants, was designed to observe how users interacted with the app and to validate the risk assessment; outcomes informed numerous changes to the app user interface. The second round of testing, with 8 participants, was performed on a functional but incomplete version of the website to test the overall functioning and content of the app; results led to a redesign of the homepage and changes to the website information architecture. The final round of usability testing, with 14 participants, was completed on a fully functional version of the website to test different options for presenting information via the homepage and the informed consent page; feedback informed the final homepage design and validated the informed consent process.

#### Final Model

**Figure 3:**
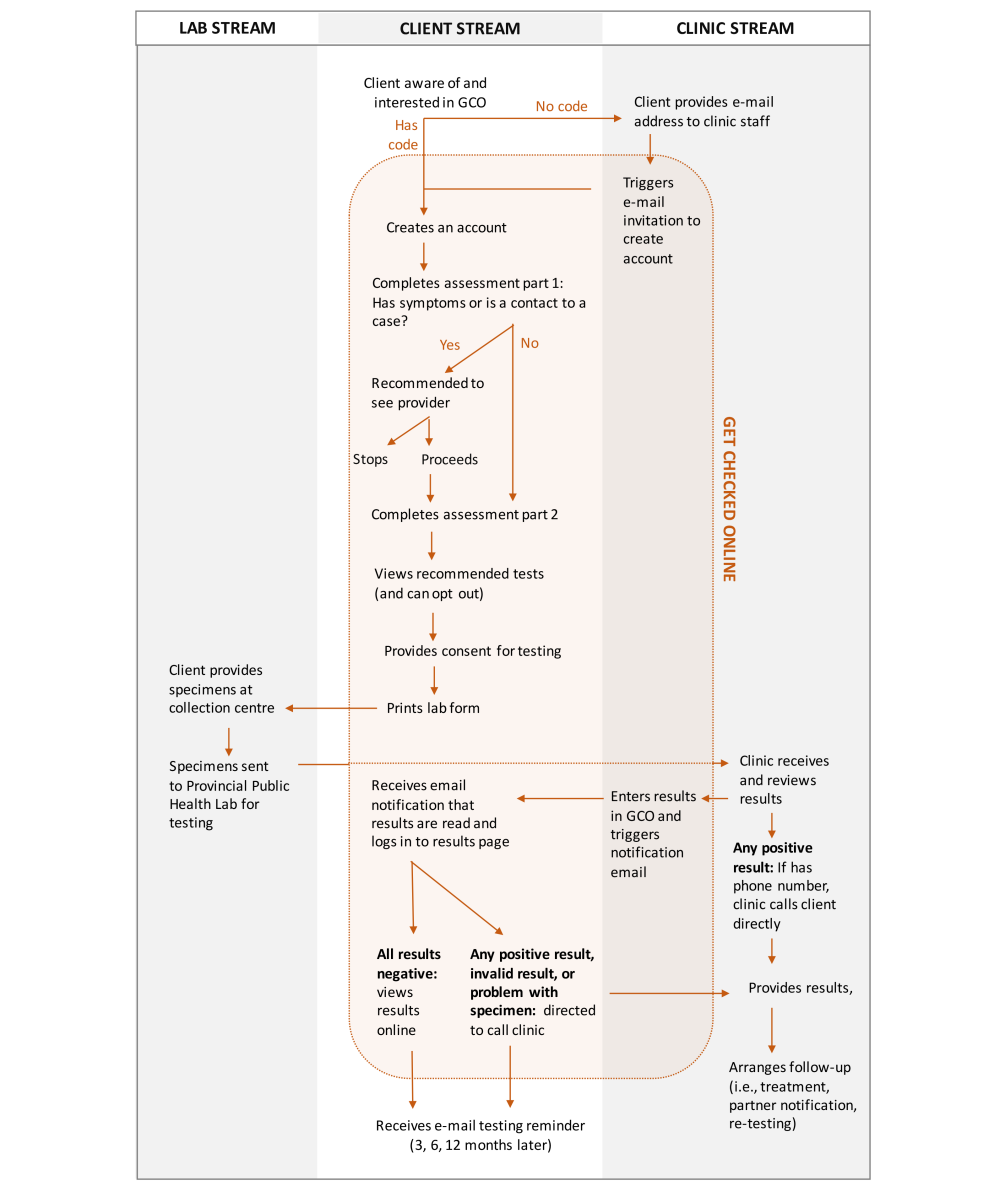
GetCheckedOnline program model demonstrating interactions between clients, clinicians, laboratories, and the GetCheckedOnline app.

The final model and flow diagram for GCO [52] is shown in Figure 3. The app screenshots and a video walk-through are included in Multimedia Appendices 1 and 2, respectively.

In brief, potential users (clients) of GCO can create an account if they have an access code (included with promotional materials, or provided by the BCCDC STI clinic or a local sexual health care provider) or have provided an email address, which is then used by BCCDC staff to send an invitation to create an account. During the account creation process, the client provides an email address to serve as their login (which is subsequently verified) and chooses a password. Both mandatory (name, date of birth, sex; not verified) and optional information (phone number, first 3 digits of postal code, ethnicity) are collected. Clients are advised to use their real name or initials although without verification pseudonyms are possible, a model consistent with low-threshold clinic-based testing services offered through BCCDC in order to reduce barriers for clients with privacy concerns. Clients are asked to provide consent to be contacted for research purposes, and must indicate their understanding of the terms of use and privacy policy. While there is no minimum age requirement for use of GCO, individuals less than 19 years are defined as children in BC legislation and can consent to their own medical care if capable. All clients indicating an age less than 19 years of age are recommended to seek testing at an STI clinic in order that capacity to provide consent can be assessed. However, as the majority of clients using GCO less than 19 years of age are expected to be capable of providing consent they are not barred from proceeding.

Clients then complete the first part of the assessment, where the questions are used to identify clients who have symptoms or are a contact to someone with an STBBI. These clients are subsequently recommended not to test through GCO but to seek standard clinical STBBI testing as other tests or immediate treatment may be indicated; clients do have the option to acknowledge the recommendation and continue with Web-based testing. The second part of the assessment includes questions used to recommend hepatitis C testing and provide tailored educational messages. Next, the GCO app provides test recommendations and educational messages as described above. Clients can deselect tests if desired, proceed to the consent page to indicate their understanding of key pretest counseling messages, and then print their laboratory test requisition (which is saved for later printing if needed).

Clients present their laboratory test requisitions, on which their name has been replaced with a unique GCO client code, at a private laboratory specimen collection site and provide urine and/or blood specimens. Specimens are shipped to the PHSA Public Health Laboratory for testing, with results reported to the BCCDC STI clinic for appropriate management. All results are entered into the GCO app by clerical staff who then trigger the app to send a notification email to the client that their results are ready to view. If all results are negative, the client can see their results in their GCO account and there is no interaction with clinic staff. If any of the results are invalid (for example, a problem occurred with the specimen), clients will view any negative test results and see a notification to call the BCCDC clinic to arrange for retesting for the invalid result. If any of the results are positive, the client can see only a message directing them to call the BCCDC clinic for their results; at the same time, if the client has provided a phone number a BCCDC clinic nurse will attempt to contact the client directly. For each client, a testing history is maintained on the app with dates of testing and test types; test results are not retained within GCO after 1 month as a privacy and security precaution. Testing reminders are set for all clients at 3 or 12 months based on the degree of sexual risk reported on the assessment questions; clients can opt out or change these settings.

#### Preparation for Implementation

Preparing for implementation of the GCO service involved numerous activities related to privacy and security, IT support, operational protocols, reporting, communications, and final validation. We collaborated closely with PHSA Privacy, Risk Management, and IT Security teams to conduct a thorough privacy impact assessment and security threat and risk assessment that included testing of the app’s security model by an external vendor (penetration testing); recommendations from these assessments were incorporated into the final app. A privacy policy and terms of use specific to the program were developed, along with administrative policies around access control, auditing and data reporting, and all were vetted with PHSA Privacy and IT Security. A new software vendor was contracted to provide ongoing app support in partnership with PHSA IT, and extensive documentation was completed to establish internal IT support around client services, network, hardware, and software maintenance. Clinical procedures for the management of GCO clients were developed in consultation with clinical and clerical team leads, and multiple training sessions were conducted to familiarize staff with procedures and the app itself. A series of reports were developed to routinely monitor testing volumes, uptake of the service and drop-off of clients at different points in the testing process. Promotional materials (ie, sign-up sheets, posters, wallet cards, and brochures) were designed and a communications package was created, including email templates for the launch announcement, sample content for social media, a one-page overview of the program, and frequently asked questions. The last step prior to the official launch of the service was a comprehensive final validation (beta-testing) of the app, specimen collection and transport, specimen testing, and clinical procedures using actual clients; where possible, we used feedback from the testing to improve the overall user experience.

#### Funding Model

The pilot phase of GCO is fully funded by the PHSA, with costs shared between the BCCDC (costs of program operation, app revision, specimen collection) and the BC Public Health Laboratory (BCPHL) (costs of laboratory testing).

## Results

### GetCheckedOnline Pilot Phase

GCO [52] went live in September 2014, with 6 participating private laboratory specimen collection sites in Vancouver, BC and is now sustained through ongoing operational funding. During this pilot phase, we promoted GCO to existing BCCDC STI clinic clients and subsequently to MSM in the Vancouver region in April 2015. This pilot phase lasted until December 2015 and an evaluation of this pilot phase is underway (eg, number of accounts created, number of specimens submitted, positivity rates, and treatment outcomes).

### How GetCheckedOnline Will be Evaluated

As recommended for the evaluation of eHealth interventions, we will use mixed-methods to evaluate the impact of GCO at individual, population, and health service delivery levels [53,54] (Table 4). Our objectives and outcomes of interest were identified from stakeholder consultations and by reviewing the eHealth implementation literature (eg, acceptability, mitigation of testing barriers, risk behavior, HIV knowledge, treatment and follow-up, uptake, reach). These will be evaluated using 5 methods (Textbox 1) funded through research grants obtained from the Canadian Institutes of Health Research [Gilbert et al, unpublished data 2011 and 2014].

**Five methods to evaluate objectives and outcomes.**

Web-based survey of GetCheckedOnline (GCO) and British Columbia Centre for Disease Control (BCCDC) sexually transmitted infection (STI) clinic clients following a Web- or clinic-based testing encounter (baseline) and 3 months later

•To measure acceptability of GCO; identify characteristics of GCO compared with clinic clients; compare baseline and short-term HIV knowledge, and risk behavior.

Web-based surveys and community intercept surveys of men who have sex with men (MSM)

•To measure acceptability of GCO; measure awareness and diffusion of GCO among networks; identify characteristics associated with uptake; assess reach to MSM most at-risk of infection.

Interviews with individuals testing through GCO

•To measure acceptability of GCO; identify if and how GCO mitigates testing barriers.

An administrative data cohort using retrospective and prospective longitudinal testing data for GCO and BCCDC STI clinic clients

•To measure acceptability (repeated use) of GCO; identify differences in testing, treatment and partner notification, test frequency, and infection rates.

Analysis of GCO and BCCDC STI clinic health services data (eg, tests conducted, number of clinic and drop-in visits, estimates of physician and nursing time)

•To determine changes in STI clinic staff configuration, staff tasks, overall clinic capacity, and laboratory testing volumes following GCO implementation.

**Table 4:** Evaluation matrix showing level of potential impact, objectives, data collection methods, and metrics.

Level of impact	Objective to determine	Data collection method(s)	Outcome measures
**Individual**			
	The acceptability of GCO^a^ (among both clients using the service and prospective clients)	Virtual cohort	Percentage and characteristics of clients who repeat-test
		Web-based client survey	Self-reported satisfaction and willingness to refer a friend
		Web-based community survey	Intention to use GCO (prospective clients)
		Client interviews	Qualitative analysis of comments on experience with GCO
	How GCO mitigates existing barriers to accessing STI^b^/HIV testing	Client interviews	Analysis of self-described factors which facilitate or limit clients’ opportunities to access in-clinic or Web-based STI/HIV testing
	If GCO clients have any short-term differences in risk behavior and posttest HIV knowledge in comparison to clinic-based clients receiving traditional in-person pre/posttest counseling	Web-based client survey (0 vs 3 months)	Risk behavior measures; 5-point true/false scale including items related to HIV transmission, risk reduction, testing, and public health follow-up
	If outcomes differ for clients testing positive via GCO (ie, are less likely to access STI treatment, or to be reached by public health for follow-up including partner notification)	Virtual cohort	Percent of those who test positive who access treatment and public health follow-up
**Population**			
	The diffusion of GCO into priority populations (ie, men who have sex with men in Phase 1)	Web-based community survey	Percent of respondents who have heard of GCO, used GCO, and seen promotional materials
	The client characteristics associated with uptake and nonuptake of GCO	Web-based community survey	Ethnicity, education, income, STI/HIV testing history, sexual risk behaviors, perceptions of GCO, use of other health services and Web-based services
	Whether GCO reaches individuals who are most at-risk of infection	Web-based client survey	Measures of sexual risk behavior
		Web-based community survey	Measures of sexual risk behavior
	Whether GCO clients have higher rates of infection than those testing in-clinic	Virtual cohort	Incidence of infection (HIV, chlamydia, gonorrhea, syphilis)
		Web-based community survey	Percent reporting recent STI or HIV diagnosis
	If GCO results in increased test frequency and earlier diagnosis among individuals most at-risk of infection	Virtual cohort	Percent of clients who repeat-test and intertest intervals (including interval between positive test and last negative test)
**Health services delivery**			
	What changes in staff configuration and tasks will occur as GCO is integrated with existing clinic sexual health services	Sexual health systems data	Estimates of total/aggregate clerical and clinical staff time spent entering test results into system, seeing asymptomatic clients in-clinic, delivering test results, and following-up with positive cases; number of episodes and estimated clerical time spent on GCO user support
	If the introduction of GCO increases the capacity of existing clinic-based sexual health services		Number of drop-in appointments and turn-aways ; number and types of STI/HIV tests conducted
	The impact on laboratory testing volume as a result of introducing GCO		Number and types of STI/HIV tests conducted

^a^GCO: GetCheckedOnline.

^b^STI: sexually transmitted infection.

#### Model Changes Following Implementation

We made 2 major changes to the GCO model following implementation, reflecting further refinement based on the findings of the planning and development phases:

In the fall of 2015, we revised the risk assessment questions in tandem with an update of our preliminary HEIA in order to improve their appropriateness for clients of diverse gender identities. We also made revisions to prospectively collect the necessary variables to validate clinical prediction rules (CPR) developed by our team for urine testing for chlamydia and gonorrhea, and blood testing for HIV. These CPR were developed using test results from over 30,000 asymptomatic clients at STI clinics across BC [55,56]. Once these are further validated using prospective GCO data, we aim to include these CPRs prior to promoting GCO outside of our target populations, where our current model for recommending tests may not be appropriate.

In February 2016, we added self-collected rectal and throat swabs for chlamydia and gonorrhea testing. Swabs are recommended for MSM clients if reporting giving oral or receiving anal sex during the assessment step, women if reporting receptive anal sex (as per routine clinical practice). While always intended for future versions of GCO, we accelerated the inclusion of swabs as their absence was flagged as a clinical risk during provider consultations and a potential exacerbation of health inequities for MSM. Clients are given self-collection kits at the private laboratory collection sites when providing urine and/or blood samples, and clients can either self-swab on-site or take home for self-collection and return. Self-collection instruction guides (Multimedia Appendix 3) were developed through reviewing examples found on the Internet with 2 focus groups of potential users, and pilot tested with 12 users who found the guides easy to understand, sensitive to various genders and sexual identities, and conducive to successful self-collection [57].

## Discussion

### A Unique Opportunity

GCO is the first comprehensive Web-based STBBI testing program in Canada, with few global counterparts, and is integrated with existing sexual health services. It represents a new paradigm for offering testing services that we believe has great potential to reach populations in BC that have high rates of infection and face the greatest barriers to accessing testing. With other health authorities recognizing this potential, and GCO’s alignment with recent provincial strategies and funding for expanding HIV testing services in BC [58], planning for scale-up to other regions began in early 2014, with the first specimen collection sites coming on board in March 2016. We have a unique opportunity to comprehensively study the implementation, impact, and transferability of a Web-based health service intervention within the Canadian health care system and across diverse populations and settings in BC. We will be conducting the research necessary to determine if we have successfully achieved our objectives both in Vancouver and in subsequent scale-up, paying particular attention to examining whether GCO improves or exacerbates existing health inequities (ie, whether the rhetoric of eHealth interventions matches the reality of their implementation) [45].

While we did not adopt a specific theoretical framework, our approach to the development of GCO was most consistent with van Gemert-Pijnen and colleagues’ [36] principles for a holistic approach to developing eHealth technologies by: using a participatory process with involvement of stakeholders throughout, including potential users; applying continuous evaluation cycles that are iterative, flexible, and dynamic; considering conditions necessary for implementation from the outset; recognizing that GCO will change the organization of health care; use of persuasive design techniques; and use of advanced methods to assess impact. As others have recommended for successful development of eHealth apps, we also believe that a critical factor in the development of GCO was the skill set of our multidisciplinary team, including early establishing of research partnerships [59]. We would also particularly emphasize the iterative, flexible, and dynamic nature of the formative evaluation and work needed to develop GCO, which we have described as a sequential series of discrete steps out of necessity. In reality, these activities were overlapping, interconnected, and mutually reinforcing of our final program model.

### Challenges With Developing GetCheckedOnline

An astute reader will have noted that while funded in 2009, GCO was not implemented until 2014, which was longer than we had anticipated. As a complex health system intervention involving multiple sectors, the development of GCO was dependent on the capacity and competing priorities of key stakeholders outside (and beyond the control of) the BCCDC. For example, the PHSA and other health authorities in the greater Vancouver region underwent a reorganization and consolidation of technical and support services during this time period, leading to substantial delays in engagement of stakeholders needed to provide the internal technical support essential to developing a Web-based intervention. Characteristics of GCO were also ground-breaking within PHSA, including automated use of email notifications and allowing patients direct Web-based access to their own personal health information. Developing the policy and technical infrastructure to support these novel aspects of GCO did take time to address, yet this has paved the way for the implementation of other eHealth apps within these agencies. However, institutional barriers remain that prevent the inclusion of features that our formative research indicates are desired by potential users of GCO. Most notably, our requirement for GCO clients to print a laboratory requisition was widely regarded as a barrier to using the service, yet receipt and storage of all laboratory requisitions is a laboratory accreditation requirement. Electronic ordering of laboratory tests is not yet widespread in Canada outside of specific electronic medical record systems such as in hospitals. Our private laboratory partner, which carries out the specimen collection is in the process of establishing electronic ordering of laboratory tests that we anticipate will be in place in the next year. This will permit barcode scanning from a client’s smartphone, which we are planning to include in GCO once this is possible.

### Implications for Development of eHealth Interventions

Our experience speaks to the challenges of developing and implementing novel, complex eHealth interventions, and adds weight to recommendations to expand technology adoption models to consider the role of broader implementation contexts that both facilitate and challenge the development and uptake of Web-based/digital health services [60,61]. The organizational context is particularly important; for example, in our experience, the commitment of ongoing operational funds, and a health agency environment that seeks to foster innovations in health care, have been critical to the successful implementation of GCO.

It is striking that while developing and advancing eHealth interventions is widely prioritized, there is relatively little practical guidance on their implementation. We hope that our detailed description of the steps taken to plan and develop GCO will be helpful not just to other jurisdictions developing similar Web-based testing programs, but more broadly to developers of similarly complex interventions that are integrated within health care systems.

## Appendix

Multimedia Appendix 1 Screenshots of the GCO application.

Multimedia Appendix 2 Video walk-through of the GCO application.

Multimedia Appendix 3 Self-collection instruction guides for oral and rectal swabs.

## Abbreviations

BC: British Columbia
BCCDC: British Columbia Centre for Disease Control
BCPHL: BC public health laboratory
CCWG: community consultation working group
CIC: clinical integration committee
CPR: clinical prediction rules
GCO: GetCheckedOnline
GWG: GetCheckedOnline Working Group
HEIA: health equity impact assessment
HIV: human immunodeficiency virus
ISC: Internet Services Committee
IT: information technology
MSM: men who have sex with men
PHSA: Provincial Health Services Authority
STBBI: sexually transmitted and blood-borne infections
STI: sexually transmitted infection
TWC: technical working group
