# Supplementary material for: Get Checked… Where? The Development of a Comprehensive, Integrated Internet-Based Testing Program for Sexually Transmitted and Blood-Borne Infections in British Columbia, Canada
Source: JMIR Res Protoc. 2016 Sep 20;5(3):e186. doi: 10.2196/resprot.6293 (PMC5050385; doi:10.2196/resprot.6293)

GetCheckedOnline Screenshots

Homepage

This is the landing page for GetCheckedOnline. From here, clients can create a new account, sign-in to their existing account or learn more about how the site works.

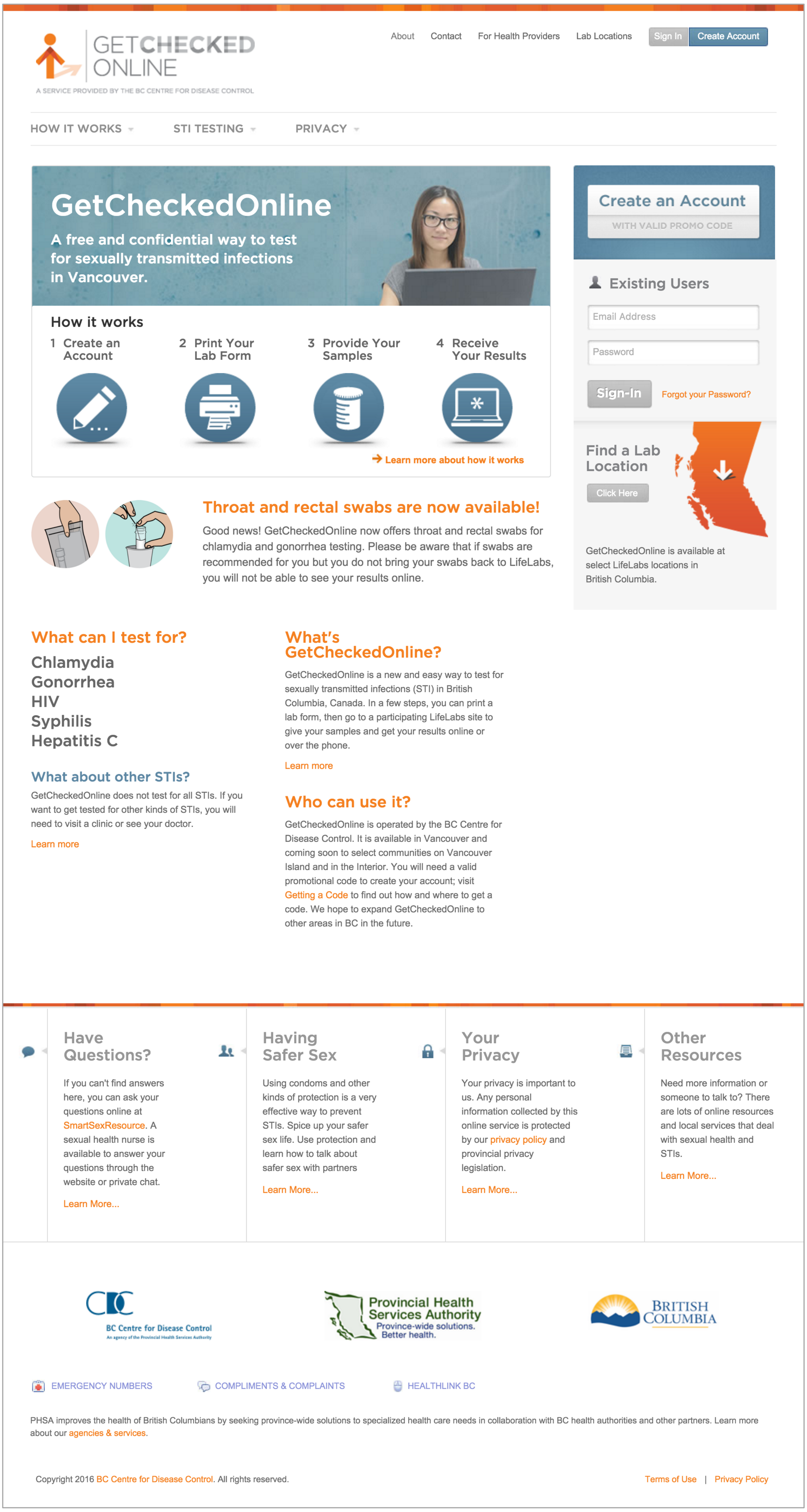

Content Pages: How it works

This section is a collection of pages describing how GetCheckedOnline works and how to use it.

GETCHECKED ONLINE

A SERVICE PROVIDED BY THE BC CENTRE FOR DISEASE CONTROL

About | Contact | For Health Providers | Lab Locations | Sign In | Create Account

HOW IT WORKS | STI TESTING | PRIVACY

How it works

- How GetCheckedOnline works
- Getting a code
- Creating an account
- Creating a lab form
- Giving samples
- Getting test results
- Where to get treatment
- Setting test reminders

How it works

Getting Started

GetCheckedOnline is a free and confidential online sexually transmitted infection (STI) testing service provided by the BC Centre for Disease Control (BCCDC). To get started, follow these steps:

Create an account. Enter your promotional code or click on the link in your invitation email to set up your account. You will need to provide some personal information, including name, date of birth, gender, city and phone number. This is so the lab can process your samples and we can contact you about your results.

Create a lab form. Complete a sexual history questionnaire to find out what tests are recommended for you. Read the consent to testing page and then print out your lab form.

Give samples at a lab. Take your lab form to a participating LifeLabs location. You will not be asked to show ID or your BC Care Card. At the lab, you will be asked to give a blood and/or urine sample, and you may be given a swab kit to take home. Do not bring your lab form to the BC Centre for Disease Control.

Get your test results. You will receive an email from GetCheckedOnline letting you know when your test results are ready. If any of your results are positive or inconclusive, a nurse will contact you to talk about treatment or retesting.

Your GetCheckedOnline account will be linked to your electronic chart at the Provincial STI Clinic at BCCDC using a unique number. This is so that we can provide you with the best care possible.

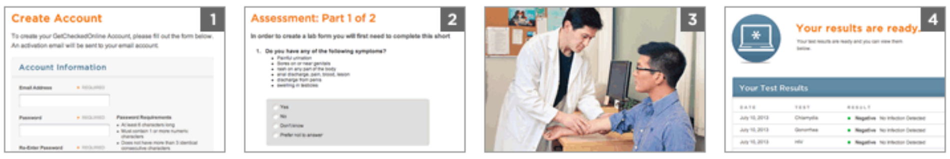

Who can use GetCheckedOnline?

At this time, GetCheckedOnline is available in Vancouver, Victoria, Duncan, Kamloops and Nelson in BC.

If you are coming to a BCCDC STI clinic in Vancouver for testing, you can sign up for GetCheckedOnline at the clinic. We will send you an email invitation with a link to set up your account. You can also use a promotional code to create your GetCheckedOnline account. Codes are available at a number of locations and through various promotional campaigns in each participating town or city. Click here to find out where and how to get a code.

GetCheckedOnline is a project of the BC Centre for Disease Control. During the first phases of this program, we will be evaluating the service to see how acceptable it is to people. We hope to expand this service to other areas of the province in the future.

When should I use GetCheckedOnline?

Before you get started, you should find out if GetCheckedOnline is for you or if it is better to see a health care provider in person. GetCheckedOnline is a great service if you:

- Want to keep up with regular STI testing.
- Do not want to see a health care provider for STI testing every time.
- Have trouble getting to a clinic for testing.

There are times when it is better to see a health care provider. To get the best care, we recommend that you see a health care provider when:

- You or your sex partners have symptoms of an STI, such as discharge, pain, itching or sores on the genitals.
- You had sex with someone who has an STI or a sex partner has told you to get tested.
- You need tests that GetCheckedOnline does not offer.
- You had sex without your consent (sexual assault).
- You need a printed copy of your test results with your name on it.

If you need a printed copy of your test results, you can test in person at a BCCDC STI clinic, a public health or walk-in clinic, or at your family doctor. You will need to show ID when testing and picking up your results.

Have Questions?

If you can't find answers here, you can ask your questions online at SmartSexResource. A sexual health nurse is available to answer your questions through the website or private chat.

Learn More...

Having Safer Sex

Using condoms and other kinds of protection is a very effective way to prevent STIs. Spice up your safer sex life. Use protection and learn how to talk about safer sex with partners

Learn More...

Your Privacy

Your privacy is important to us. Any personal information collected by this online service is protected by our privacy policy and provincial privacy legislation.

Learn More...

Other Resources

Need more information or someone to talk to? There are lots of online resources and local services that deal with sexual health and STIs.

Learn More...

Content Pages: STI Testing

This section has general information about STI and HIV testing.

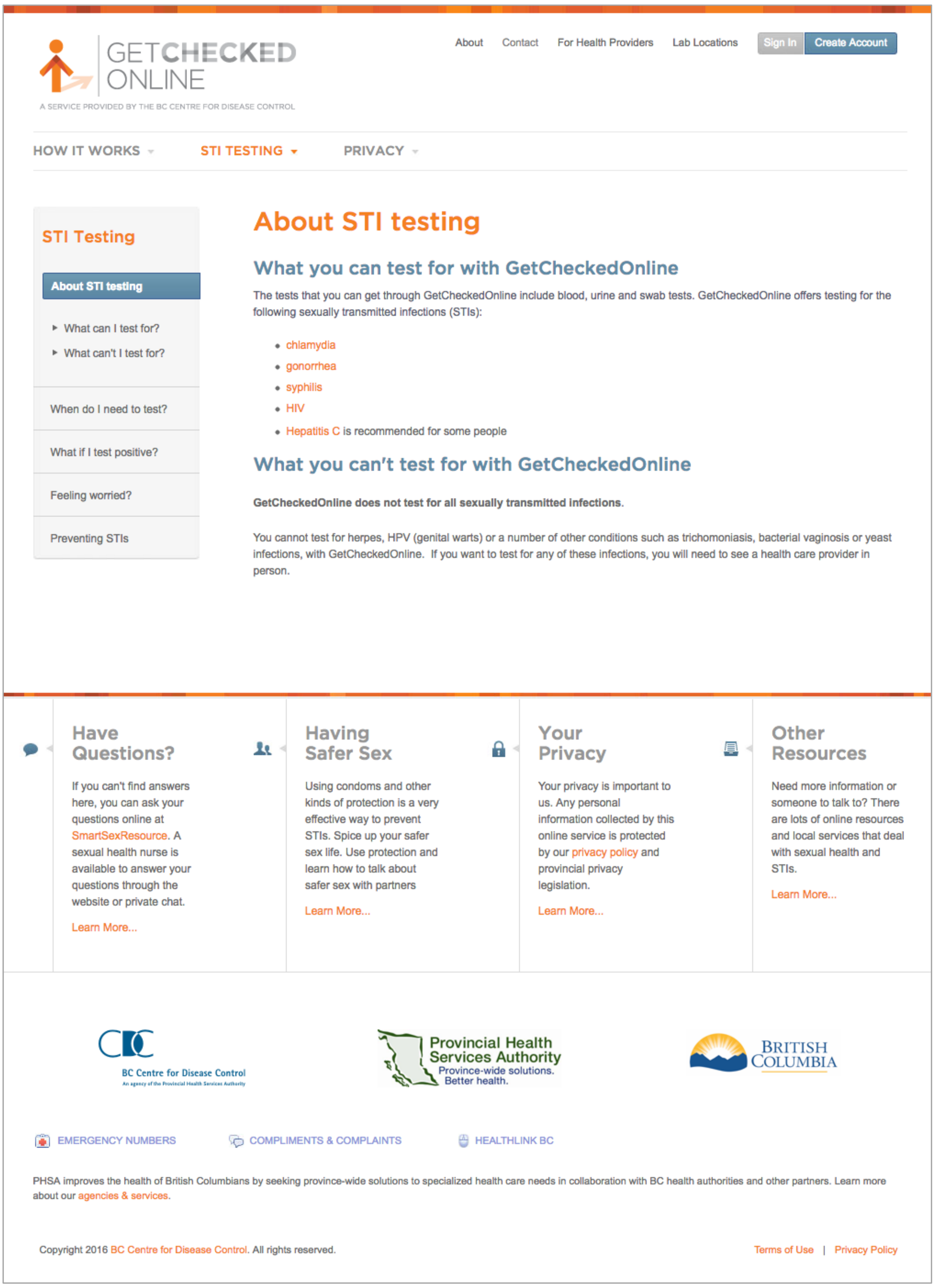

GETCHECKED ONLINE

A SERVICE PROVIDED BY THE BC CENTRE FOR DISEASE CONTROL

About | Contact | For Health Providers | Lab Locations | Sign In | Create Account

HOW IT WORKS | STI TESTING | PRIVACY

STI Testing

- About STI testing
  - What can I test for?
  - What can't I test for?
- When do I need to test?
- What if I test positive?
- Feeling worried?
- Preventing STIs

About STI testing

What you can test for with GetCheckedOnline

The tests that you can get through GetCheckedOnline include blood, urine and swab tests. GetCheckedOnline offers testing for the following sexually transmitted infections (STIs):

- chlamydia
- gonorrhea
- syphilis
- HIV
- Hepatitis C is recommended for some people

What you can't test for with GetCheckedOnline

GetCheckedOnline does not test for all sexually transmitted infections.

You cannot test for herpes, HPV (genital warts) or a number of other conditions such as trichomoniasis, bacterial vaginosis or yeast infections, with GetCheckedOnline. If you want to test for any of these infections, you will need to see a health care provider in person.

Have Questions?

If you can't find answers here, you can ask your questions online at SmartSexResource. A sexual health nurse is available to answer your questions through the website or private chat.

Learn More...

Having Safer Sex

Using condoms and other kinds of protection is a very effective way to prevent STIs. Spice up your safer sex life. Use protection and learn how to talk about safer sex with partners

Learn More...

Your Privacy

Your privacy is important to us. Any personal information collected by this online service is protected by our privacy policy and provincial privacy legislation.

Learn More...

Other Resources

Need more information or someone to talk to? There are lots of online resources and local services that deal with sexual health and STIs.

Learn More...

BC Centre for Disease Control | Provincial Health Services Authority: Province-wide solutions. Better health. | British Columbia

EMERGENCY NUMBERS | COMPLIMENTS & COMPLAINTS | HEALTHLINK BC

PHSA improves the health of British Columbians by seeking province-wide solutions to specialized health care needs in collaboration with BC health authorities and other partners. Learn more about our agencies & services.

Copyright 2016 BC Centre for Disease Control. All rights reserved.

Terms of Use | Privacy Policy

Content Pages: Privacy

This section has pages that deal with the privacy aspects of GetCheckedOnline.

GETCHECKED ONLINE

A SERVICE PROVIDED BY THE BC CENTRE FOR DISEASE CONTROL

About | Contact | For Health Providers | Lab Locations | Sign In | Create Account

HOW IT WORKS | STI TESTING | PRIVACY

Privacy

- **Privacy FAQs**
- Protecting my privacy
- Privacy policy

Privacy FAQs

Your privacy is important to us. Any personal information that you provide on GetCheckedOnline will be kept secure and confidential. The information that we ask for on GetCheckedOnline is similar to the information required when you see a health care provider in person.

You may have a lot of questions about your privacy and how it is protected. Please contact us if you have questions that are not answered here.

- What personal information is collected when I test with GetCheckedOnline?
- Why do you collect this information?
- Where is my information stored?
- Who has access to my information?
- What information is visible online when I sign into GetCheckedOnline?

1. What personal information is collected when I get tested with GetCheckedOnline?

To provide you with the best possible care when you use GetCheckedOnline, we need to collect some personal information from you. This is similar information that a health care provider collects when you visit them in person.

Your contact information.

To set up an account with GetCheckedOnline, you will need to provide your name, email address, date of birth and gender. Your phone number is optional but is highly recommended so that we can contact you right away with any positive test results. Your name will not show anywhere on your lab form. Instead, you will be given a unique GetCheckedOnline Client Code that will be printed on your lab form and used to process your tests at the lab.

A brief sexual history.

To assess what tests and additional health information you may need, you will be asked to complete an online assessment. The assessment asks questions about your sexual history, condom use and sexual partners. This information is also used to recommend how often you should get tested. You have the option "I prefer not to answer" if you are not comfortable with giving an answer to a question.

2. Why do you collect this information?

To provide you with the best possible care, we need your contact information and a brief sexual history so that we can:

Contact you with results.

If your test results are positive (meaning you have a sexually transmitted infection), a nurse from the BC Centre for Disease Control will contact you by phone or email to talk about the next steps. The nurse will not give any information about your tests or results in the email or voice mail message. You will be asked to phone the clinic and speak with a nurse who will tell you your results over the phone. If your results are positive, the nurse will speak with you about treatment and talking to your partners.

Report to public health.

Positive test results will be shared with public health using your GetCheckedOnline Client Code and the contact information you provide. Public health will use this information to track and monitor STIs in BC, and to develop programs for STI prevention and sexual health promotion.

For HIV, you have the option to not have your contact information reported to public health. All personal information reported to public health is confidential and there are protections in place to ensure your privacy.

Evaluate GetCheckedOnline.

You have the option to stay informed about opportunities to evaluate this service. If you choose, we will send you an email to tell you when we are conducting evaluations such as surveys or interviews. You can choose at that time if you would like to participate. We will not contact you to participate in program evaluation without your permission.

3. Where is my information stored?

Whether you get tested for STIs through GetCheckedOnline or through a BC Centre for Disease Control (BCCDC) STI clinic, your personal information and test results become part of your health record. When you test through GetCheckedOnline, these are the places where your health information is stored.

- **Provincial Health Services Authority (PHSA) databases.** GetCheckedOnline will store your account information and your testing history, which includes the dates you tested and what tests you had. Negative test results will be stored in GetCheckedOnline for 30 days and then deleted. Positive test results are never stored in GetCheckedOnline. When you test using GetCheckedOnline, you are considered to be a client of the BCCDC STI clinic. This means that your account information and test results will be entered into the BCCDC clinical database. Your record in the BCCDC clinical database will be linked to your GetCheckedOnline account by a unique number. Both GetCheckedOnline and the BCCDC clinical database are hosted on servers located within British Columbia and are subject to strict PHSA security and privacy standards.
- **Provincial Laboratory Information Systems**. In BC, laboratory test results are kept in a provincial system called the Provincial Laboratory Information System (PLIS). It is a secure database that stores personal health information and test results for patients across BC. Whether you use GetCheckedOnline or visit a BCCDC STI clinic, your personal information and test results will be stored here. Through GetCheckedOnline, a unique code will be used instead of your name. How much a health care provider can see of your record depends on their role and health care providers who are not providing you with care will not be able to access your record.
- **Laboratory databases**. The laboratory that collects your samples will store your GetcheckedOnline Client Code, gender and date of birth in their database, along with the tests that were ordered. The Provincial Public Health laboratory that tests your samples will store your GetcheckedOnline Client Code, gender and date of birth in their database, along with your test results.
- **Health Authority Databases**. In BC, positive test results are reported to public health in the area where a person gets tested. This report includes your GetcheckedOnline Client Code, gender and date of birth. If you test positive for a reportable STI, the health authority will store your personal information and test results in a secure database.

4. Who has access to my information?

Your personal health information will be accessed only by health care providers who need to look at it in the context of your health care. How much a health care provider can see of your record depends on their role. Health care providers who are not providing you with care will not be able to access your record.

- **BC Centre for Disease Control (BCCDC) staff**: Nurses from the STI Clinic at BCCDC need your information so that they can contact you about your test results and give you information about treatment and follow-up.
- **Heath Authority staff**: Public health gets a record of all positive test results in order to monitor the number of infections in the health authority. In some situations, a public health nurse from the health authority may continue with follow-up after a positive result to ensure that you get the care and support you need.

5. What information is visible online in my GetCheckedOnline account?

You can sign in to your GetCheckedOnline account anytime. You can update and change your contact information as needed by signing in and managing your account.

Your account is private and can only be viewed with the correct sign-in and password information. Once you are signed in, you can see the status of any tests and review your testing history. Negative test results will be stored in your GetCheckedOnline account for 30 days. Positive test results are not stored in your GetCheckedOnline account.

Have Questions? | Having Safer Sex | Your Privacy | Other Resources

Content Pages: About

This section has information about the BC Centre for Disease Control, our clinics, program evaluation and other information.

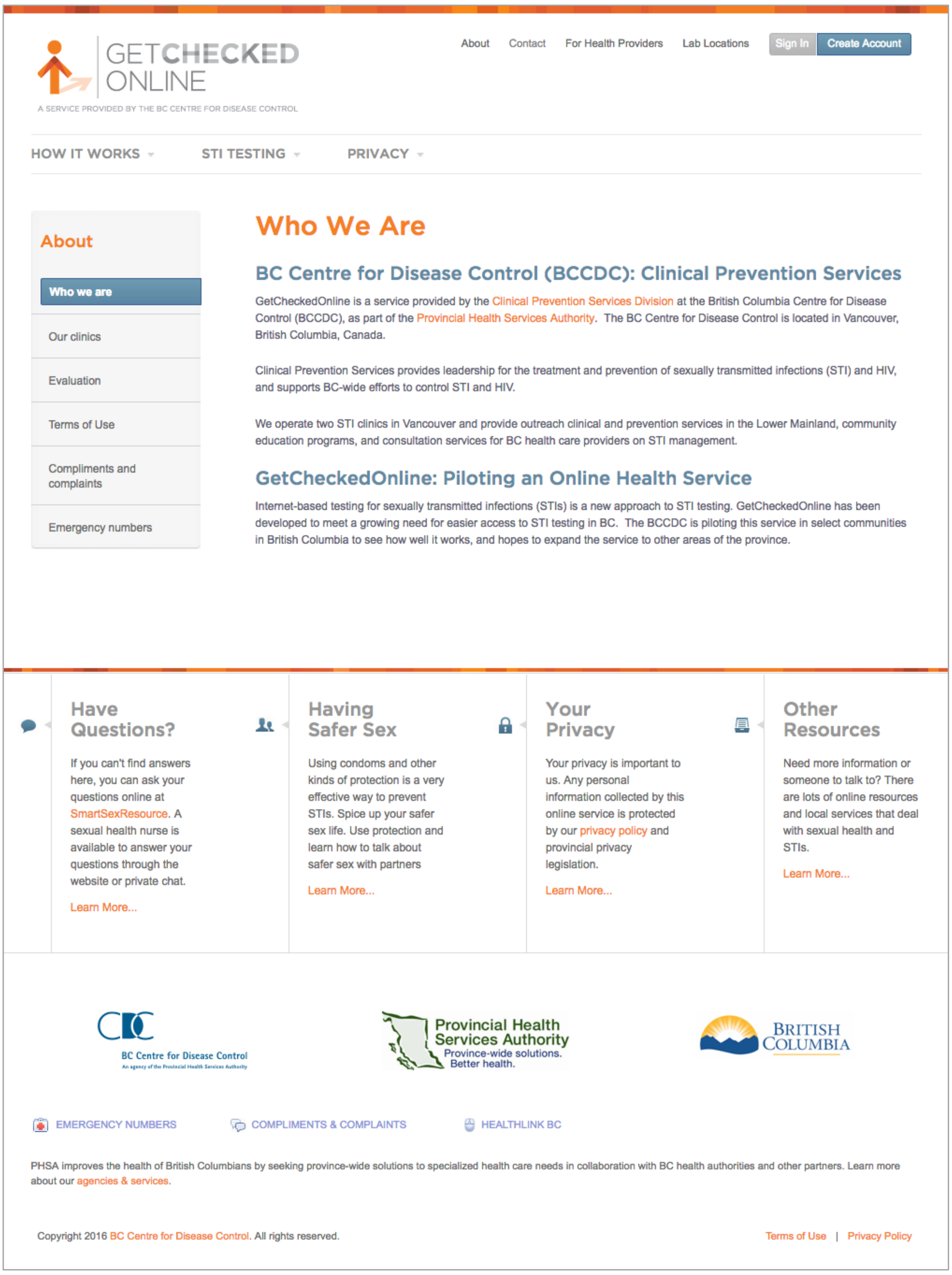

Content Pages: For Health Providers

This section has information for health providers who may be treating clients who use GetCheckedOnline. It includes information sheets about chlamydia, gonorrhea and hepatitis C as well as links to the BCCDC STI Treatment Guidelines.

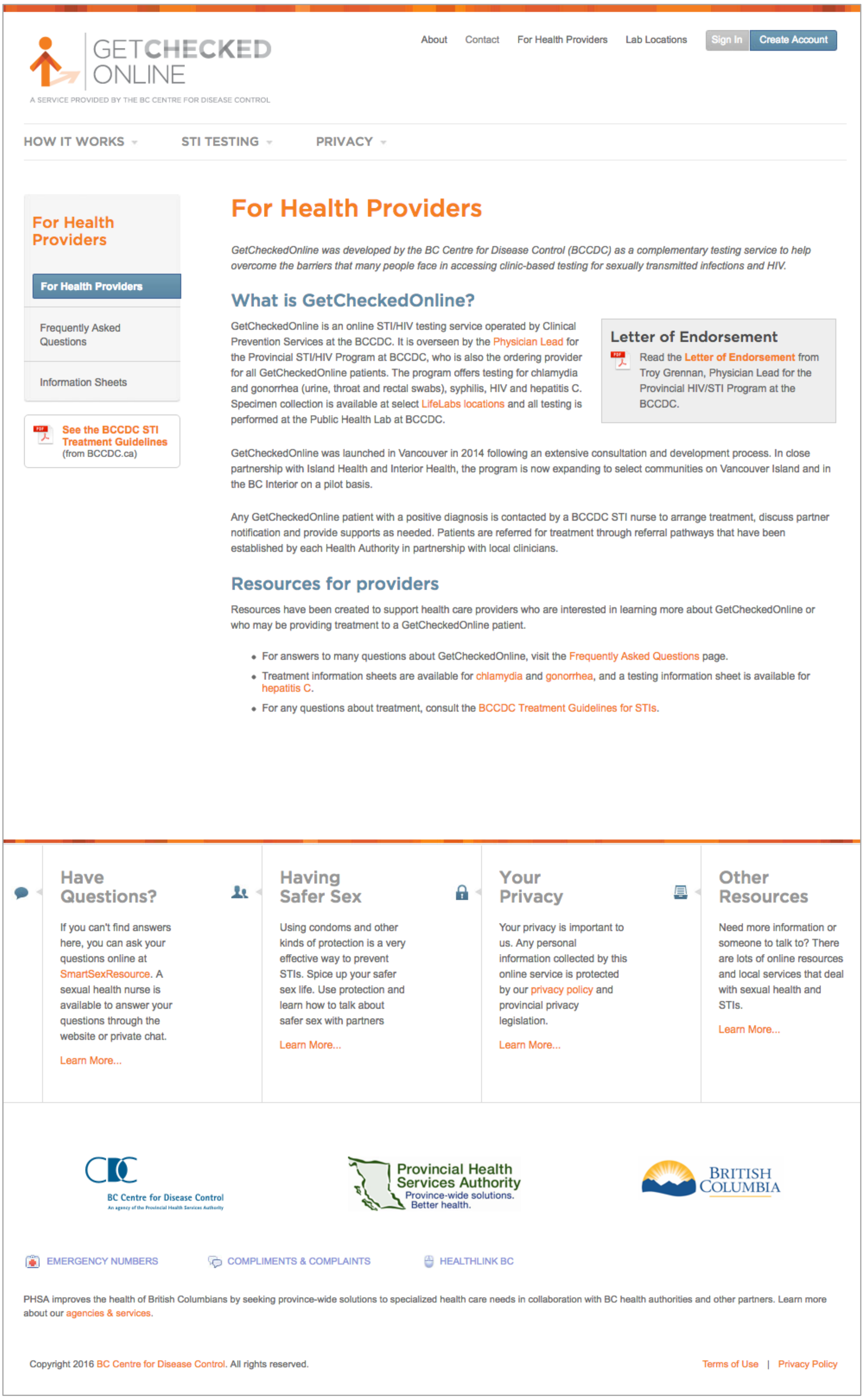

GETCHECKED ONLINE
A SERVICE PROVIDED BY THE BC CENTRE FOR DISEASE CONTROL

About | Contact | For Health Providers | Lab Locations | Sign In | Create Account

HOW IT WORKS | STI TESTING | PRIVACY

For Health Providers

- For Health Providers
- Frequently Asked Questions
- Information Sheets

See the BCCDC STI Treatment Guidelines (from BCCDC.ca)

For Health Providers

GetCheckedOnline was developed by the BC Centre for Disease Control (BCCDC) as a complementary testing service to help overcome the barriers that many people face in accessing clinic-based testing for sexually transmitted infections and HIV.

What is GetCheckedOnline?

GetCheckedOnline is an online STI/HIV testing service operated by Clinical Prevention Services at the BCCDC. It is overseen by the Physician Lead for the Provincial STI/HIV Program at BCCDC, who is also the ordering provider for all GetCheckedOnline patients. The program offers testing for chlamydia and gonorrhea (urine, throat and rectal swabs), syphilis, HIV and hepatitis C. Specimen collection is available at select LifeLabs locations and all testing is performed at the Public Health Lab at BCCDC.

Letter of Endorsement

Read the Letter of Endorsement from Troy Grennan, Physician Lead for the Provincial HIV/STI Program at the BCCDC.

GetCheckedOnline was launched in Vancouver in 2014 following an extensive consultation and development process. In close partnership with Island Health and Interior Health, the program is now expanding to select communities on Vancouver Island and in the BC Interior on a pilot basis.

Any GetCheckedOnline patient with a positive diagnosis is contacted by a BCCDC STI nurse to arrange treatment, discuss partner notification and provide supports as needed. Patients are referred for treatment through referral pathways that have been established by each Health Authority in partnership with local clinicians.

Resources for providers

Resources have been created to support health care providers who are interested in learning more about GetCheckedOnline or who may be providing treatment to a GetCheckedOnline patient.

- For answers to many questions about GetCheckedOnline, visit the Frequently Asked Questions page.
- Treatment information sheets are available for chlamydia and gonorrhea, and a testing information sheet is available for hepatitis C.
- For any questions about treatment, consult the BCCDC Treatment Guidelines for STIs.

Have Questions?

If you can't find answers here, you can ask your questions online at SmartSexResource. A sexual health nurse is available to answer your questions through the website or private chat.

Learn More...

Having Safer Sex

Using condoms and other kinds of protection is a very effective way to prevent STIs. Spice up your safer sex life. Use protection and learn how to talk about safer sex with partners

Learn More...

Your Privacy

Your privacy is important to us. Any personal information collected by this online service is protected by our privacy policy and provincial privacy legislation.

Learn More...

Other Resources

Need more information or someone to talk to? There are lots of online resources and local services that deal with sexual health and STIs.

Learn More...

BC Centre for Disease Control
An agency of the Provincial Health Services Authority

Provincial Health Services Authority
Province-wide solutions. Better health.

BRITISH COLUMBIA

EMERGENCY NUMBERS | COMPLIMENTS & COMPLAINTS | HEALTHLINK BC

PHSA improves the health of British Columbians by seeking province-wide solutions to specialized health care needs in collaboration with BC health authorities and other partners. Learn more about our agencies & services.

Copyright 2016 BC Centre for Disease Control. All rights reserved.

Terms of Use | Privacy Policy

Content Pages: Other Resources

This page contains links to other sexual health resources.

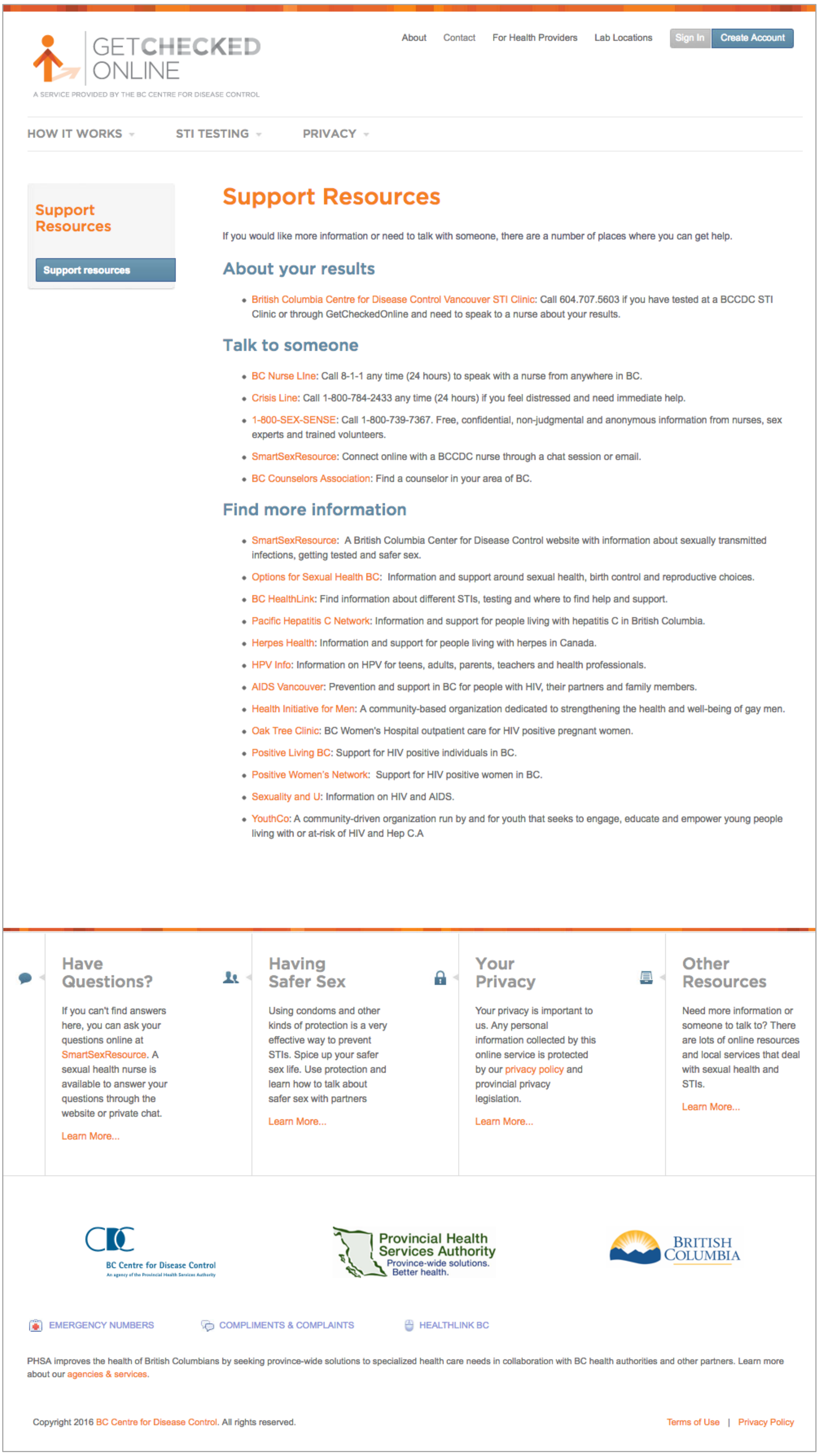

GETCHECKED ONLINE

A SERVICE PROVIDED BY THE BC CENTRE FOR DISEASE CONTROL

About | Contact | For Health Providers | Lab Locations | Sign In | Create Account

HOW IT WORKS | STI TESTING | PRIVACY

Support Resources

Support resources

Support Resources

If you would like more information or need to talk with someone, there are a number of places where you can get help.

About your results

- British Columbia Centre for Disease Control Vancouver STI Clinic: Call 604.707.5603 if you have tested at a BCCDC STI Clinic or through GetCheckedOnline and need to speak to a nurse about your results.

Talk to someone

- BC Nurse LIne: Call 8-1-1 any time (24 hours) to speak with a nurse from anywhere in BC.
- Crisis Line: Call 1-800-784-2433 any time (24 hours) if you feel distressed and need immediate help.
- 1-800-SEX-SENSE: Call 1-800-739-7367. Free, confidential, non-judgmental and anonymous information from nurses, sex experts and trained volunteers.
- SmartSexResource: Connect online with a BCCDC nurse through a chat session or email.
- BC Counselors Association: Find a counselor in your area of BC.

Find more information

- SmartSexResource: A British Columbia Center for Disease Control website with information about sexually transmitted infections, getting tested and safer sex.
- Options for Sexual Health BC: Information and support around sexual health, birth control and reproductive choices.
- BC HealthLink: Find information about different STIs, testing and where to find help and support.
- Pacific Hepatitis C Network: Information and support for people living with hepatitis C in British Columbia.
- Herpes Health: Information and support for people living with herpes in Canada.
- HPV Info: Information on HPV for teens, adults, parents, teachers and health professionals.
- AIDS Vancouver: Prevention and support in BC for people with HIV, their partners and family members.
- Health Initiative for Men: A community-based organization dedicated to strengthening the health and well-being of gay men.
- Oak Tree Clinic: BC Women's Hospital outpatient care for HIV positive pregnant women.
- Positive Living BC: Support for HIV positive individuals in BC.
- Positive Women's Network: Support for HIV positive women in BC.
- Sexuality and U: Information on HIV and AIDS.
- YouthCo: A community-driven organization run by and for youth that seeks to engage, educate and empower young people living with or at-risk of HIV and Hep C.A

Have Questions?

If you can't find answers here, you can ask your questions online at SmartSexResource. A sexual health nurse is available to answer your questions through the website or private chat.

Learn More...

Having Safer Sex

Using condoms and other kinds of protection is a very effective way to prevent STIs. Spice up your safer sex life. Use protection and learn how to talk about safer sex with partners

Learn More...

Your Privacy

Your privacy is important to us. Any personal information collected by this online service is protected by our privacy policy and provincial privacy legislation.

Learn More...

Other Resources

Need more information or someone to talk to? There are lots of online resources and local services that deal with sexual health and STIs.

Learn More...

BC Centre for Disease Control

Provincial Health Services Authority. Province-wide solutions. Better health.

BRITISH COLUMBIA

EMERGENCY NUMBERS | COMPLIMENTS & COMPLAINTS | HEALTHLINK BC

PHSA improves the health of British Columbians by seeking province-wide solutions to specialized health care needs in collaboration with BC health authorities and other partners. Learn more about our agencies & services.

Copyright 2016 BC Centre for Disease Control. All rights reserved.

Terms of Use | Privacy Policy

Create Account Pages

The account creation process often starts with entering a promo code on the homepage. Clients then fill out the Create Account form and verify their email address.

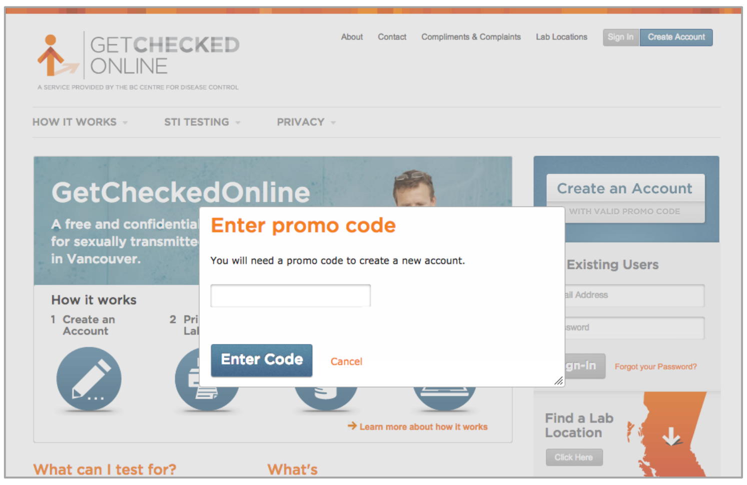

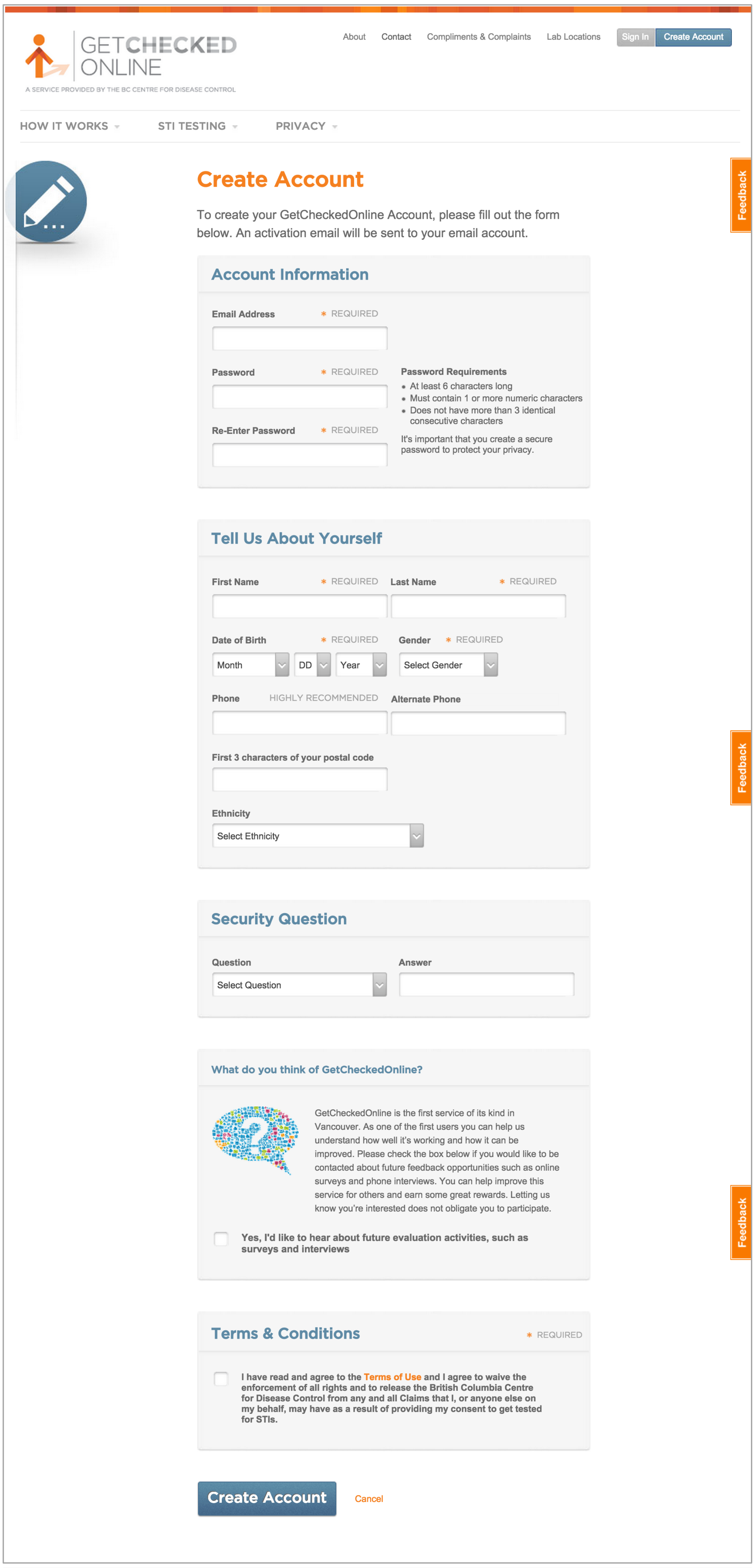

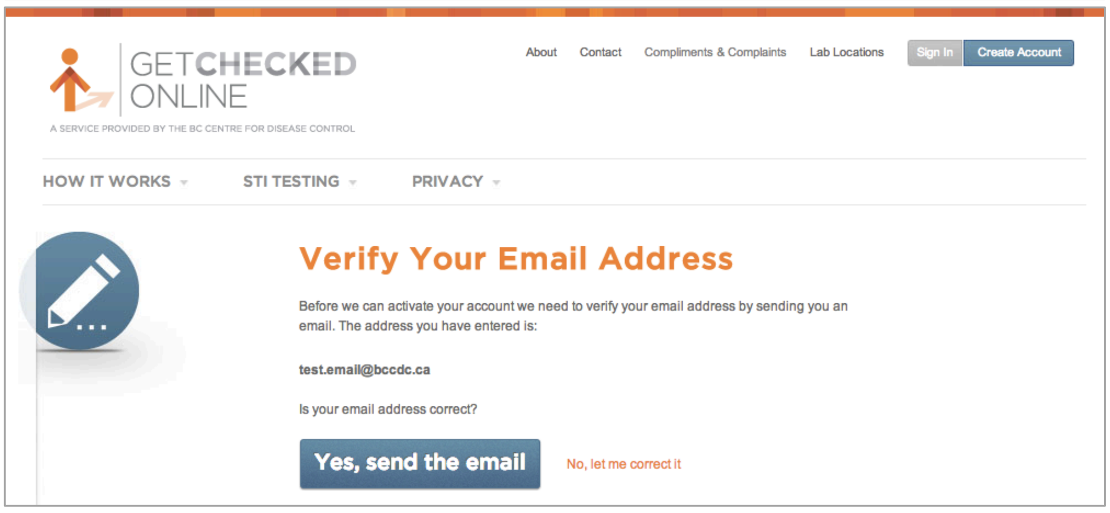

Creating a Lab Form: Tests & Results Page

Clients start the Lab Form creation process on the Tests & Results page.

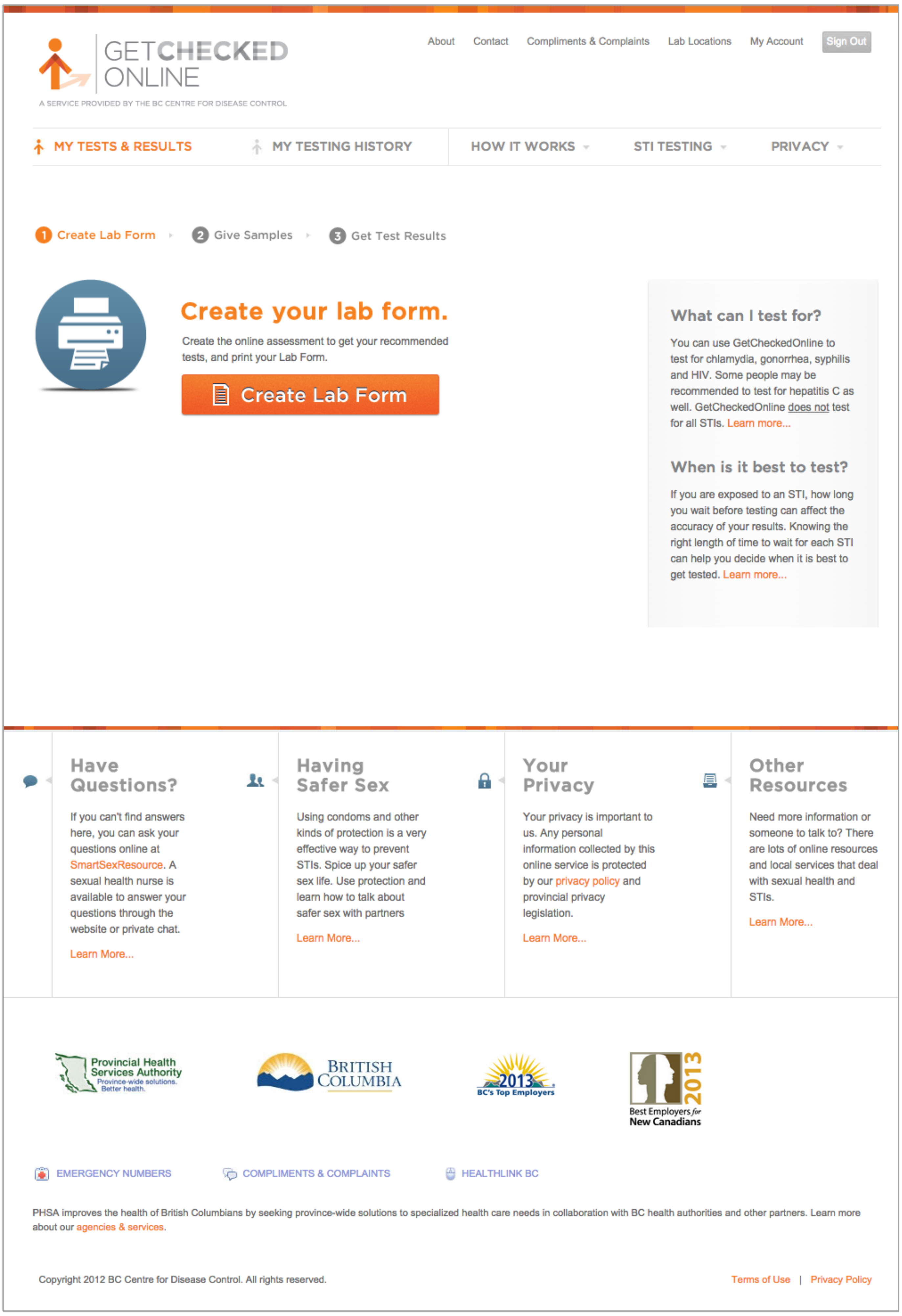

GETCHECKED ONLINE
A SERVICE PROVIDED BY THE BC CENTRE FOR DISEASE CONTROL

About | Contact | Compliments & Complaints | Lab Locations | My Account | Sign Out

MY TESTS & RESULTS | MY TESTING HISTORY | HOW IT WORKS | STI TESTING | PRIVACY

1 Create Lab Form › 2 Give Samples › 3 Get Test Results

Create your lab form.

Create the online assessment to get your recommended tests, and print your Lab Form.

Create Lab Form

What can I test for?

You can use GetCheckedOnline to test for chlamydia, gonorrhea, syphilis and HIV. Some people may be recommended to test for hepatitis C as well. GetCheckedOnline does not test for all STIs. Learn more...

When is it best to test?

If you are exposed to an STI, how long you wait before testing can affect the accuracy of your results. Knowing the right length of time to wait for each STI can help you decide when it is best to get tested. Learn more...

Have Questions?

If you can't find answers here, you can ask your questions online at SmartSexResource. A sexual health nurse is available to answer your questions through the website or private chat.

Learn More...

Having Safer Sex

Using condoms and other kinds of protection is a very effective way to prevent STIs. Spice up your safer sex life. Use protection and learn how to talk about safer sex with partners

Learn More...

Your Privacy

Your privacy is important to us. Any personal information collected by this online service is protected by our privacy policy and provincial privacy legislation.

Learn More...

Other Resources

Need more information or someone to talk to? There are lots of online resources and local services that deal with sexual health and STIs.

Learn More...

Provincial Health Services Authority — Province-wide solutions. Better health.
BRITISH COLUMBIA
2013 BC's Top Employers
2013 Best Employers for New Canadians

EMERGENCY NUMBERS | COMPLIMENTS & COMPLAINTS | HEALTHLINK BC

PHSA improves the health of British Columbians by seeking province-wide solutions to specialized health care needs in collaboration with BC health authorities and other partners. Learn more about our agencies & services.

Copyright 2012 BC Centre for Disease Control. All rights reserved.

Terms of Use | Privacy Policy

Creating a Lab Form: Risk Assessment Part 1 & Triage Screen

The Risk Assessment is split into 2 pages. Clients who say yes to question 1 or 2 on Part 1 will be shown the triage screen.

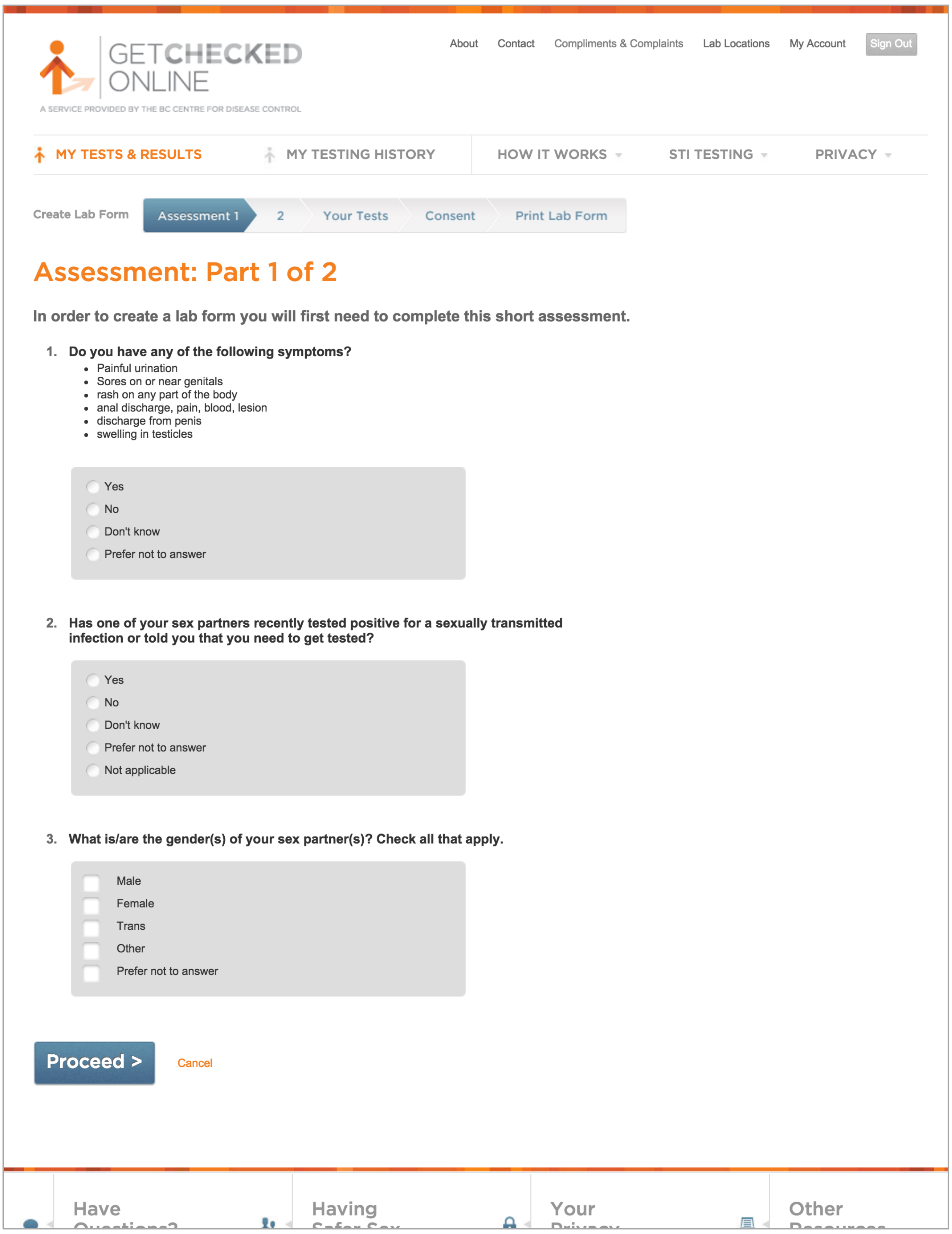

Triage screen:

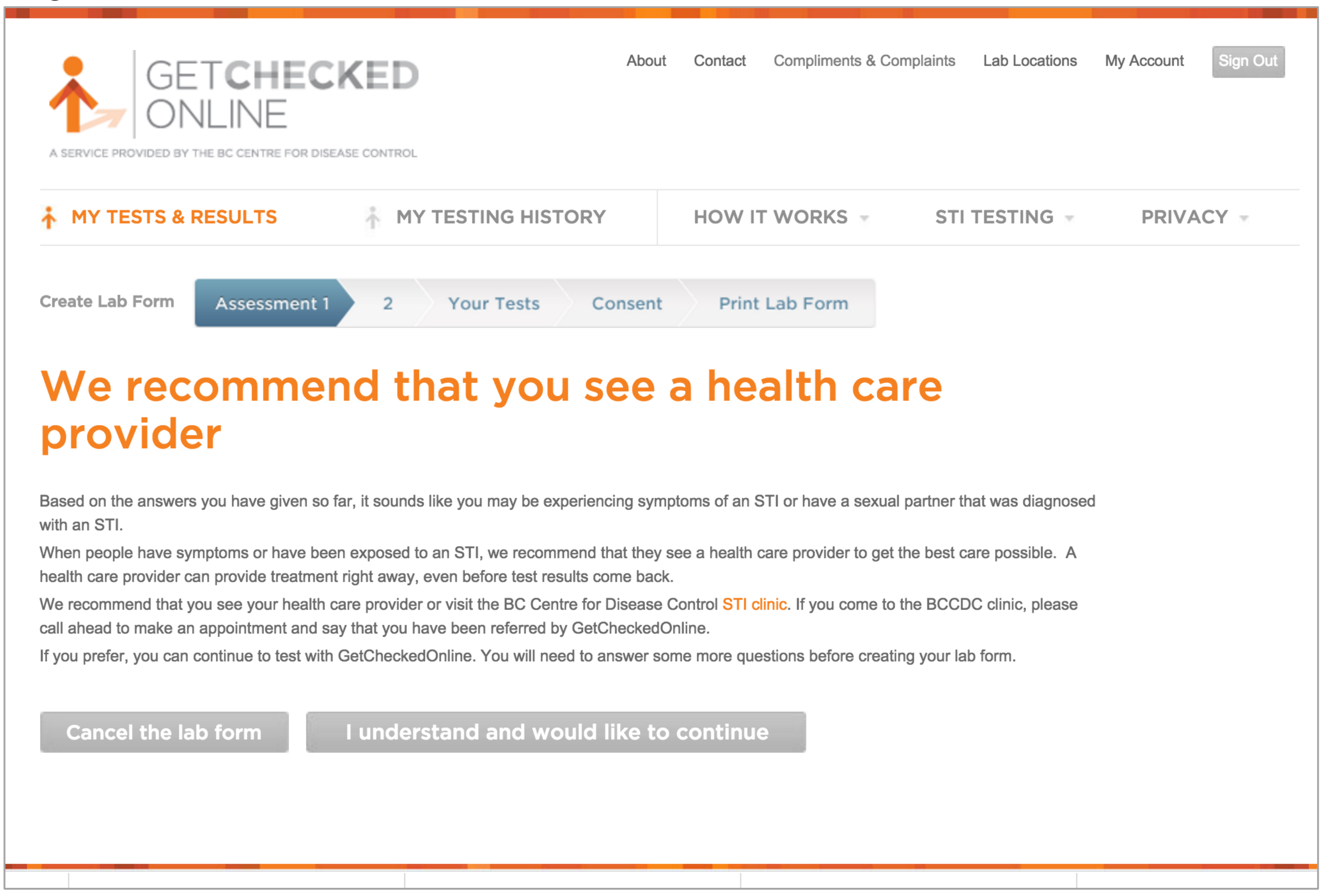

Creating a Lab Form: Risk Assessment Part 2

This is part 2 of the assessment.

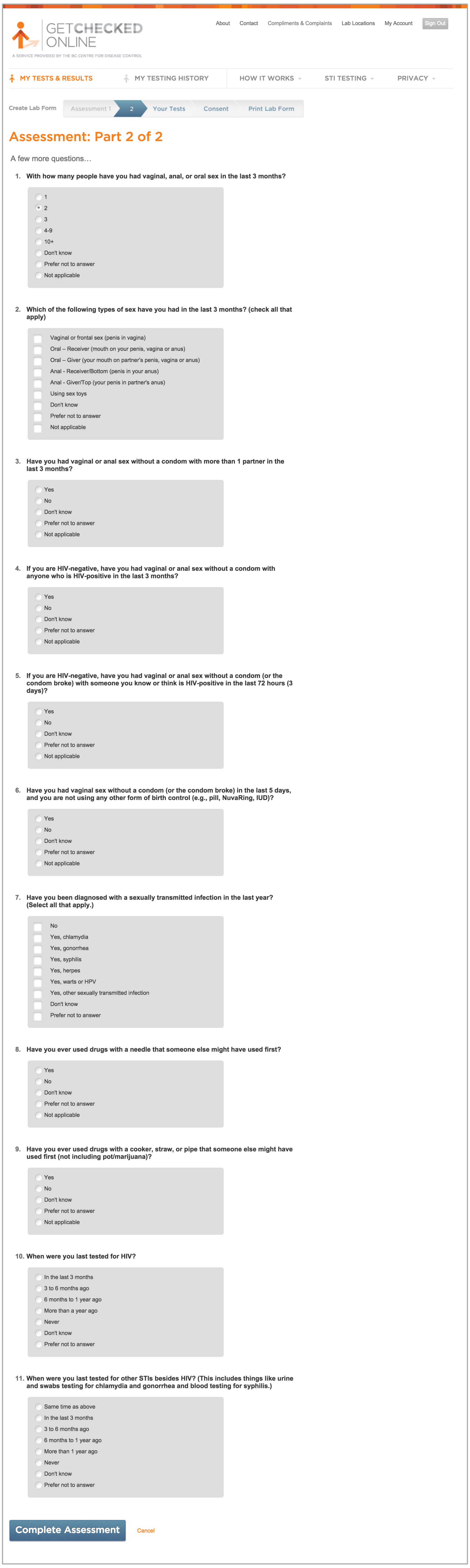

GETCHECKED ONLINE
A SERVICE PROVIDED BY THE BC CENTRE FOR DISEASE CONTROL

About | Contact | Compliments & Complaints | Lab Locations | My Account | Sign Out

MY TESTS & RESULTS | MY TESTING HISTORY | HOW IT WORKS | STI TESTING | PRIVACY

Create Lab Form: Assessment 1 › 2 › Your Tests › Consent › Print Lab Form

Assessment: Part 2 of 2

A few more questions…

1. With how many people have you had vaginal, anal, or oral sex in the last 3 months?
   - 1
   - 2
   - 3
   - 4-9
   - 10+
   - Don't know
   - Prefer not to answer
   - Not applicable

2. Which of the following types of sex have you had in the last 3 months? (check all that apply)
   - Vaginal or frontal sex (penis in vagina)
   - Oral – Receiver (mouth on your penis, vagina or anus)
   - Oral – Giver (your mouth on partner's penis, vagina or anus)
   - Anal - Receiver/Bottom (penis in your anus)
   - Anal - Giver/Top (your penis in partner's anus)
   - Using sex toys
   - Don't know
   - Prefer not to answer
   - Not applicable

3. Have you had vaginal or anal sex without a condom with more than 1 partner in the last 3 months?
   - Yes
   - No
   - Don't know
   - Prefer not to answer
   - Not applicable

4. If you are HIV-negative, have you had vaginal or anal sex without a condom with anyone who is HIV-positive in the last 3 months?
   - Yes
   - No
   - Don't know
   - Prefer not to answer
   - Not applicable

5. If you are HIV-negative, have you had vaginal or anal sex without a condom (or the condom broke) with someone you know or think is HIV-positive in the last 72 hours (3 days)?
   - Yes
   - No
   - Don't know
   - Prefer not to answer
   - Not applicable

6. Have you had vaginal sex without a condom (or the condom broke) in the last 5 days, and you are not using any other form of birth control (e.g., pill, NuvaRing, IUD)?
   - Yes
   - No
   - Don't know
   - Prefer not to answer
   - Not applicable

7. Have you been diagnosed with a sexually transmitted infection in the last year? (Select all that apply.)
   - No
   - Yes, chlamydia
   - Yes, gonorrhea
   - Yes, syphilis
   - Yes, herpes
   - Yes, warts or HPV
   - Yes, other sexually transmitted infection
   - Don't know
   - Prefer not to answer

8. Have you ever used drugs with a needle that someone else might have used first?
   - Yes
   - No
   - Don't know
   - Prefer not to answer
   - Not applicable

9. Have you ever used drugs with a cooker, straw, or pipe that someone else might have used first (not including pot/marijuana)?
   - Yes
   - No
   - Don't know
   - Prefer not to answer
   - Not applicable

10. When were you last tested for HIV?
    - In the last 3 months
    - 3 to 6 months ago
    - 6 months to 1 year ago
    - More than a year ago
    - Never
    - Don't know
    - Prefer not to answer

11. When were you last tested for other STIs besides HIV? (This includes things like urine and swabs testing for chlamydia and gonorrhea and blood testing for syphilis.)
    - Same time as above
    - In the last 3 months
    - 3 to 6 months ago
    - 6 months to 1 year ago
    - More than 1 year ago
    - Never
    - Don't know
    - Prefer not to answer

Complete Assessment | Cancel

Creating a Lab Form: Your Tests Page

Tests are recommended to clients based on their assessment answers. Clients can select and deselect tests as desired. This page can also contain educational messages.

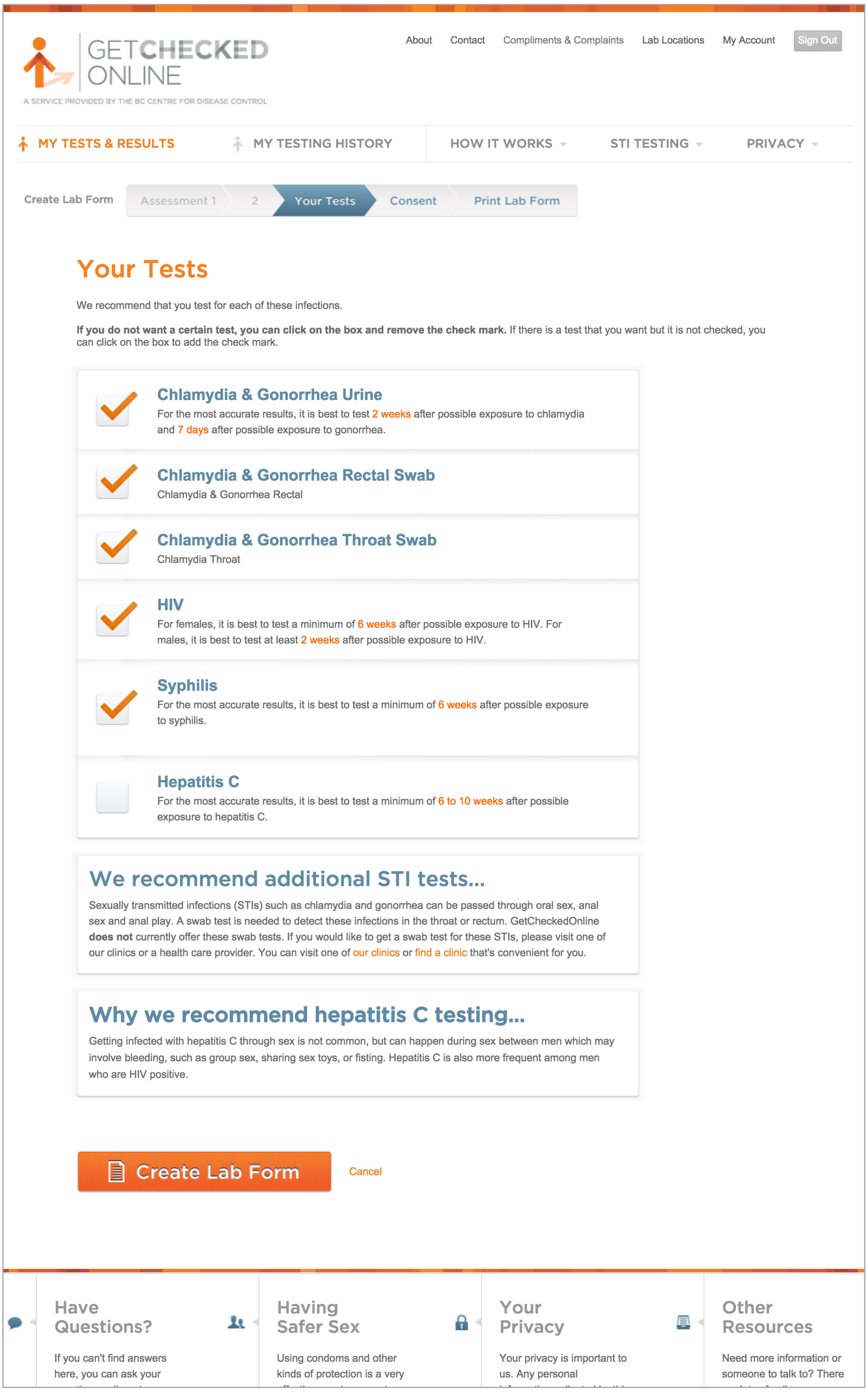

Creating a Lab Form: Consent Page

Before clients get their Lab Form they need to consent to testing. Each of the consent statements can be expanded to get more information.

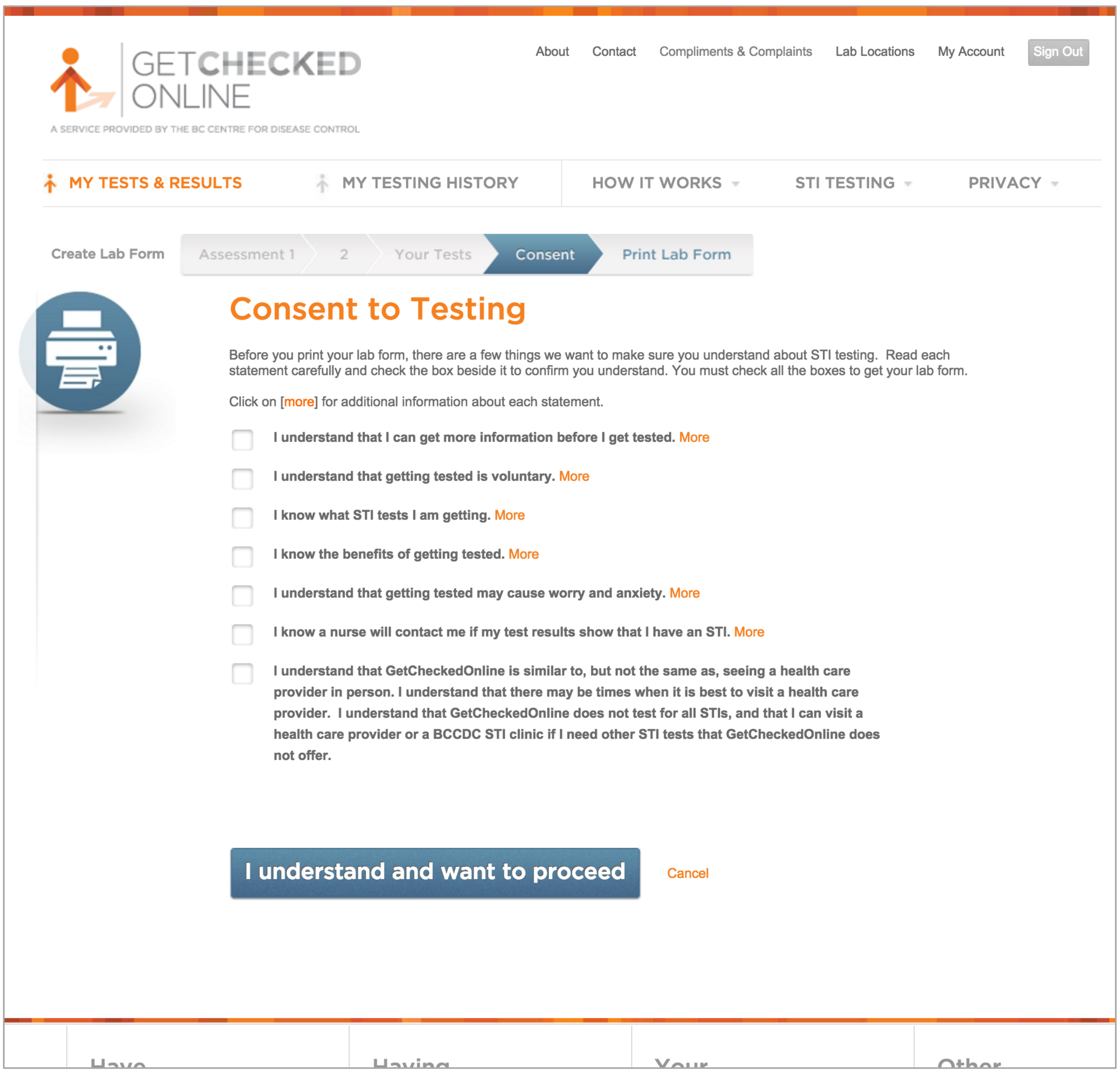

Creating a Lab Form: Print Lab Form

The last step in creating a Lab Form is the Print Lab Form page. Here the client can see a preview of the lab form and then print it.

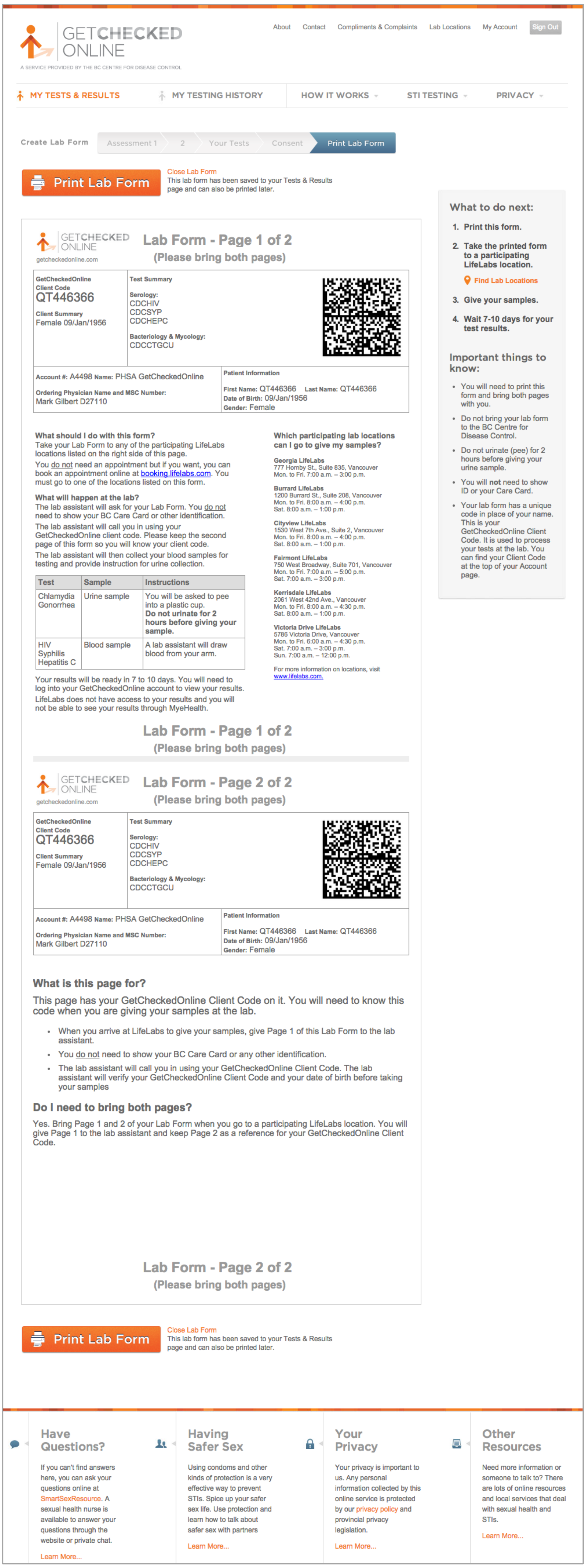

Creating a Lab Form: Tests & Results Page with Lab Form

The Tests & Results page now shows the client's pending tests. The client's testing reminders have also been set and are visible on this page.

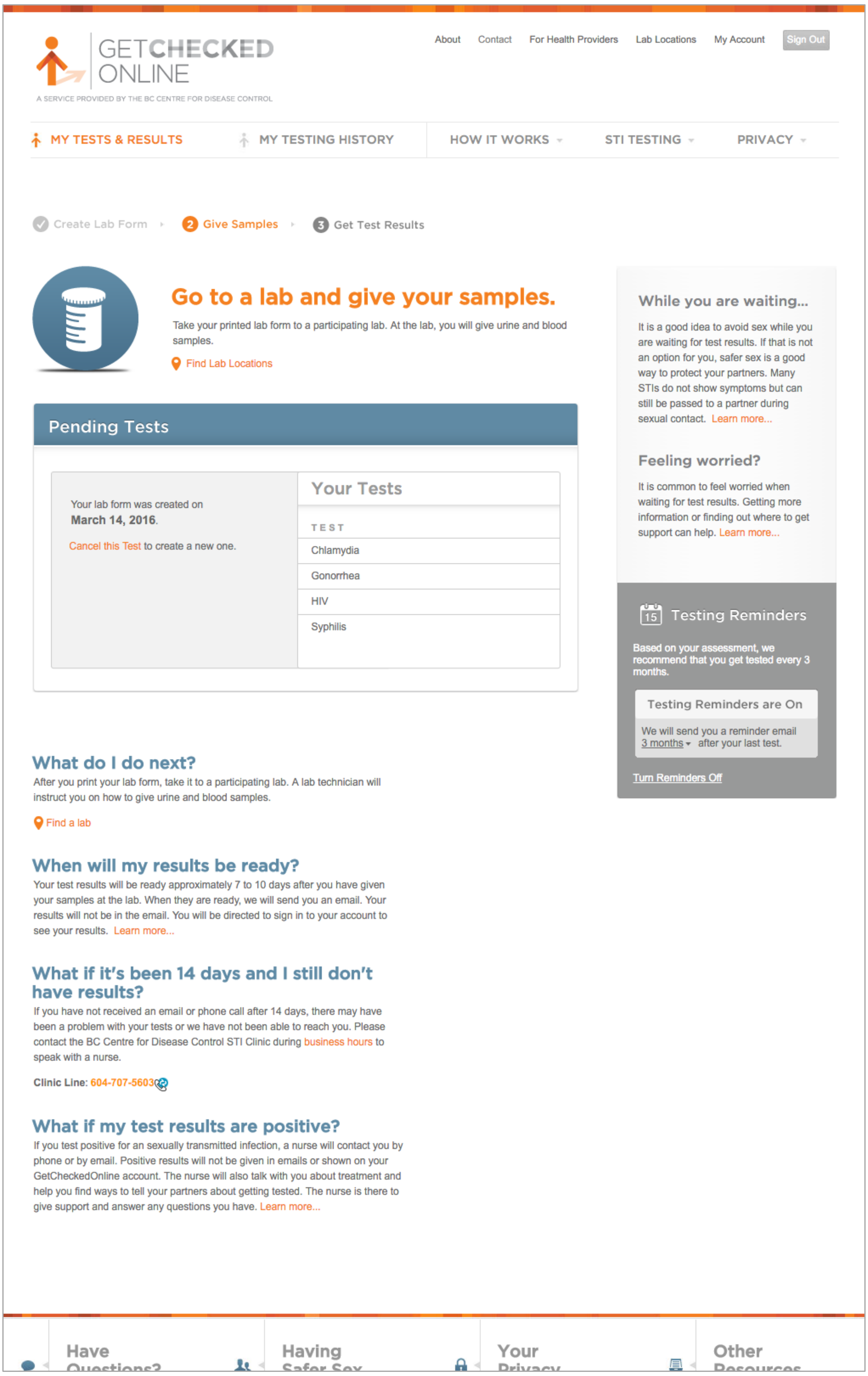

Getting Test Results: Negative Results

Negative test results are given to clients on the Tests & Results page.

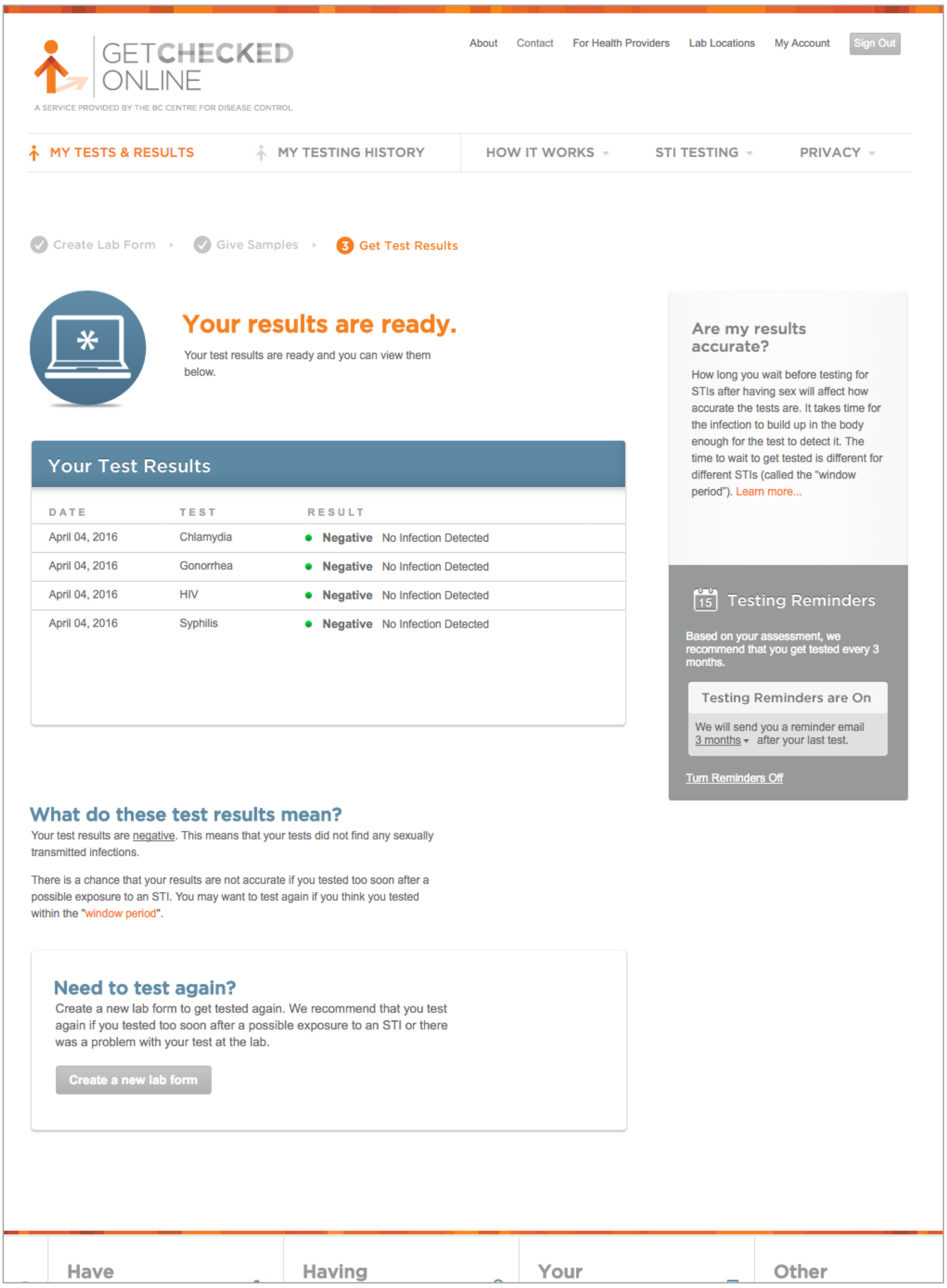

Getting Test Results: Problem with Sample & Previous Syphilis

In some cases, there may be problems with a client's test results. In these cases clients are shown an appropriate message.

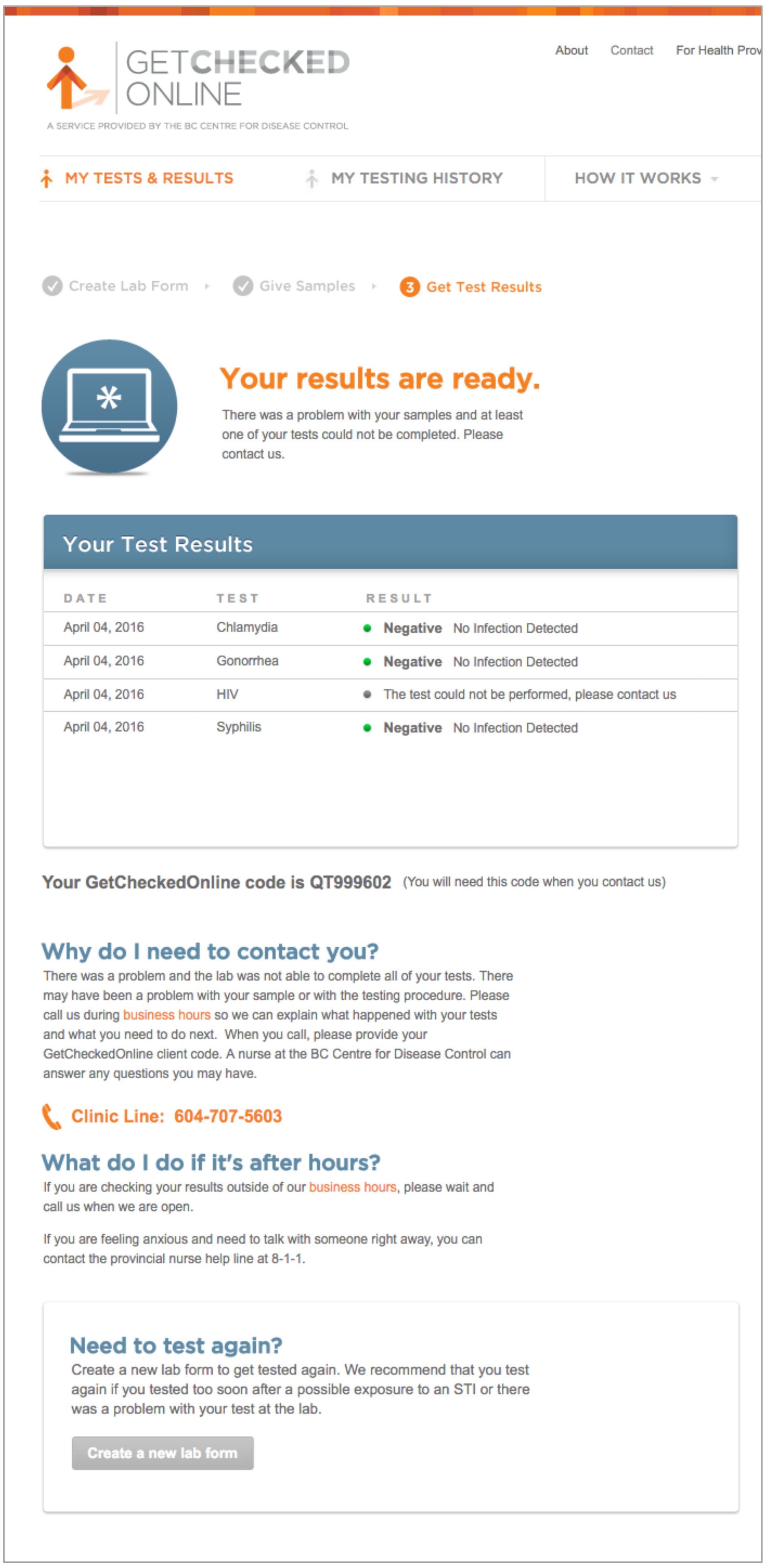

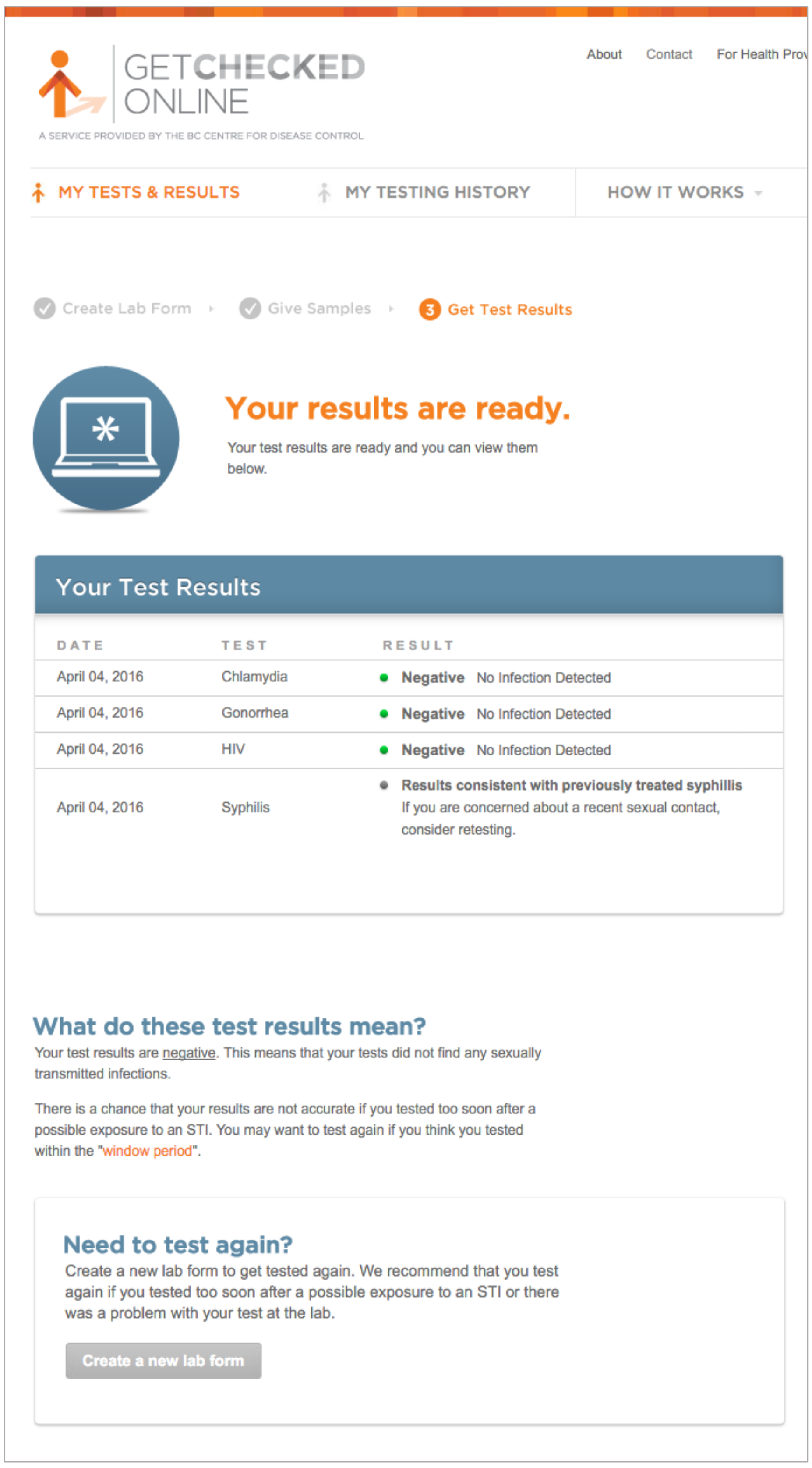

Getting Test Results: Please contact us

Clients with positive test results may be asked to contact us through a message on the Tests & Results page.

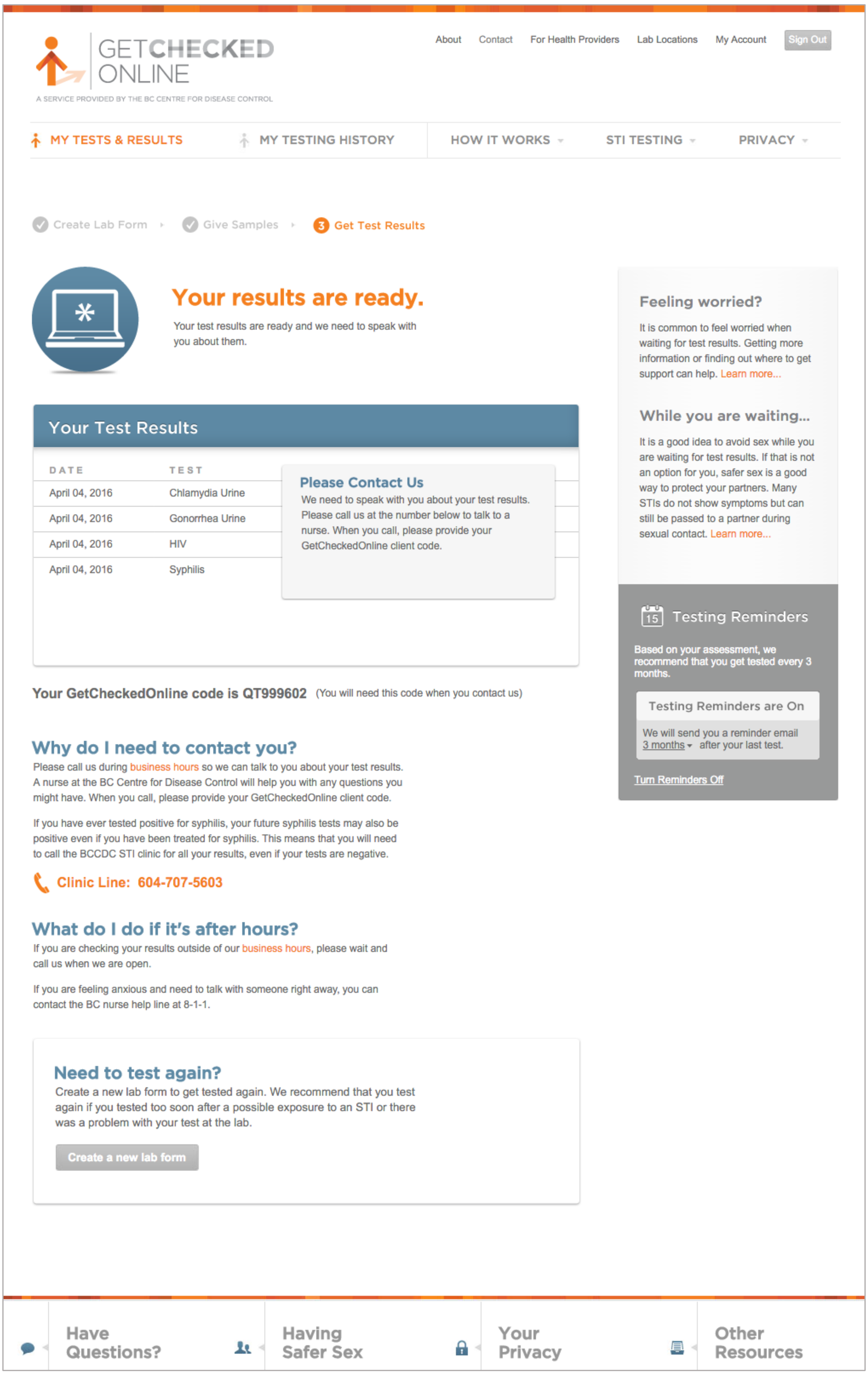

Getting Test Results: Your results are ready

Once we have contacted a client with positive test results the message on their Tests & Results page will change to indicate that their test episode is completed.

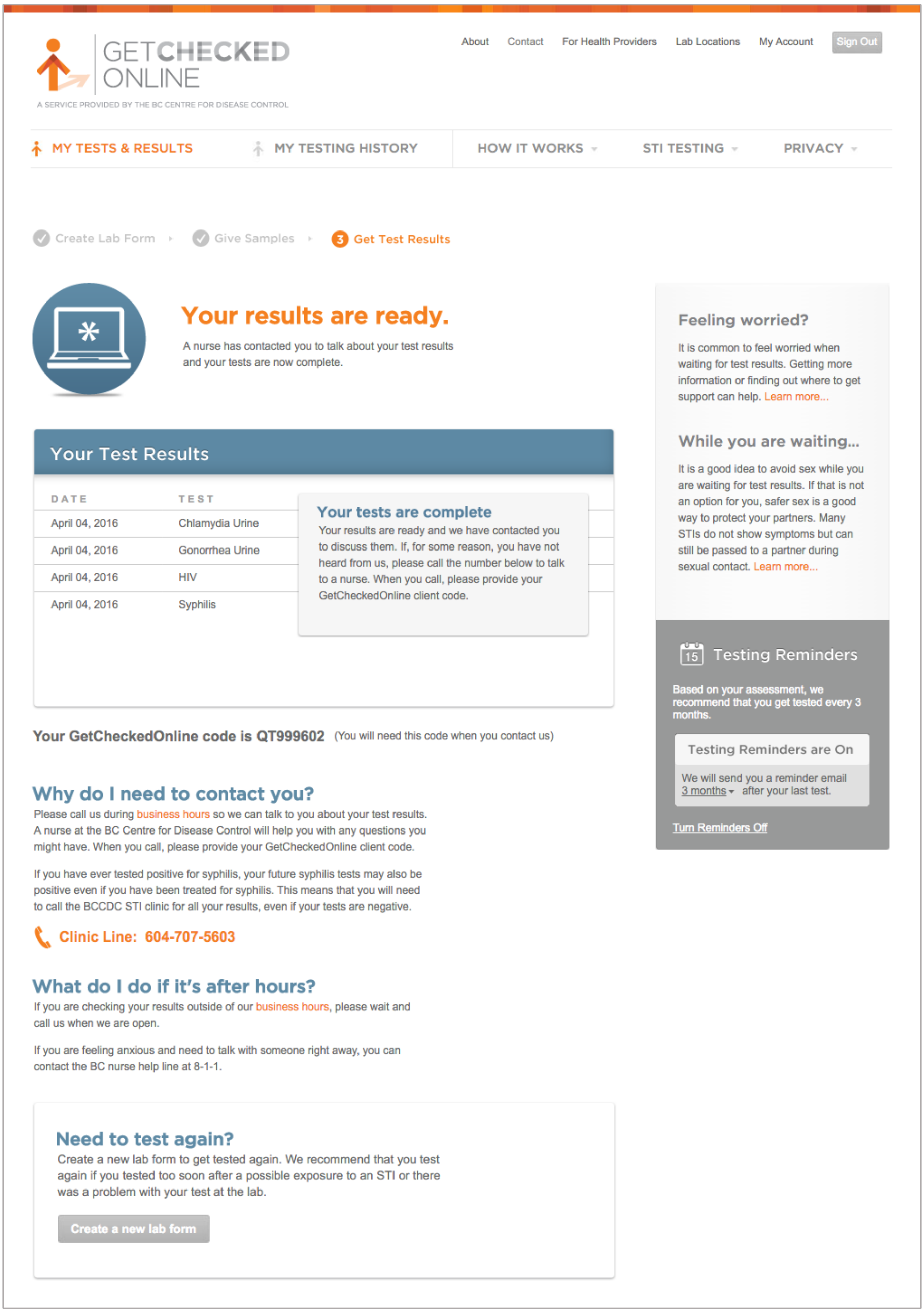

Testing History

The testing history page shows the client's previous test episodes.

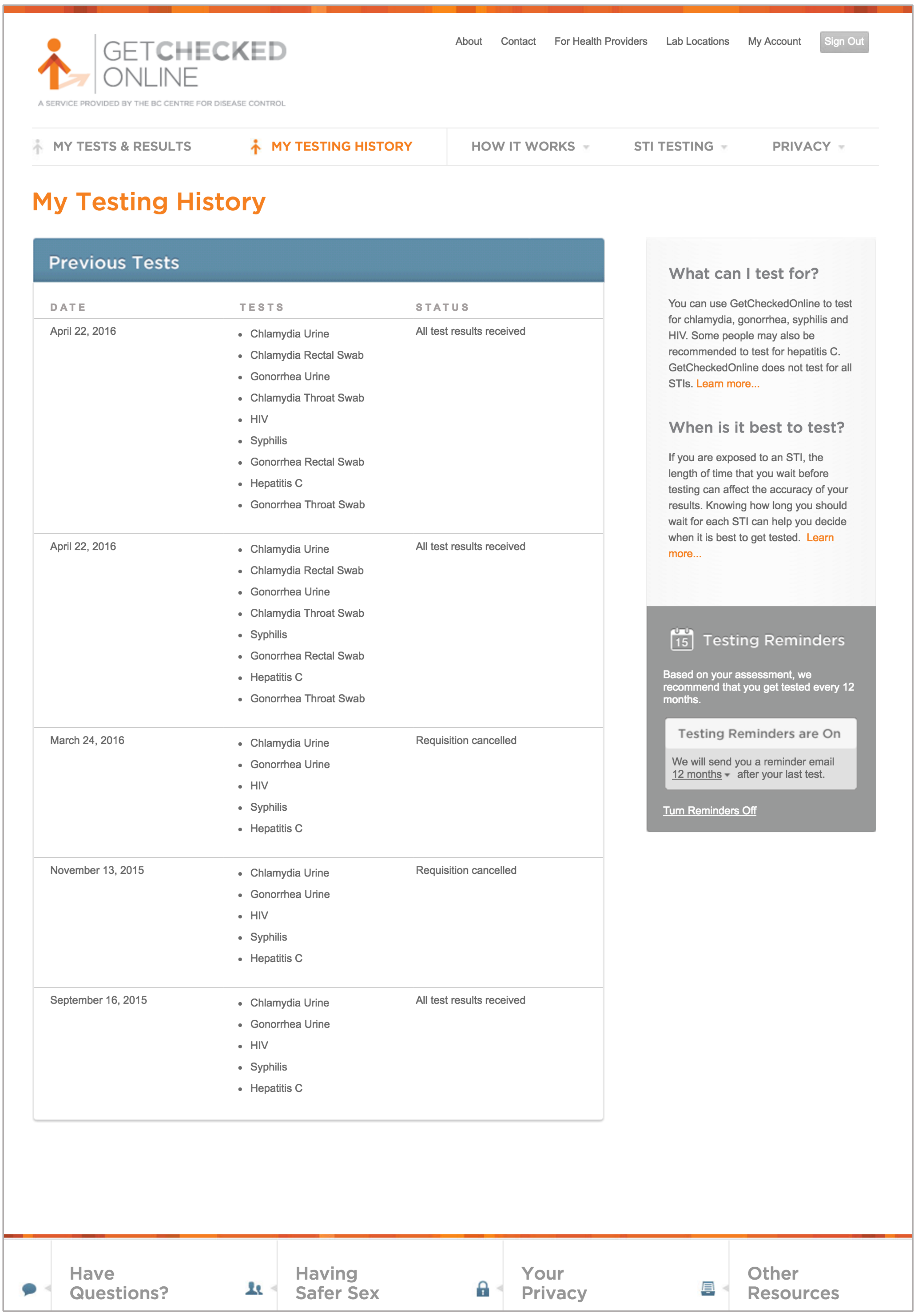

Lab Locations: Map and List pages

The Lab Locations page shows clients where they can go to give their specimens. Clients can either view a map, or a list of locations.

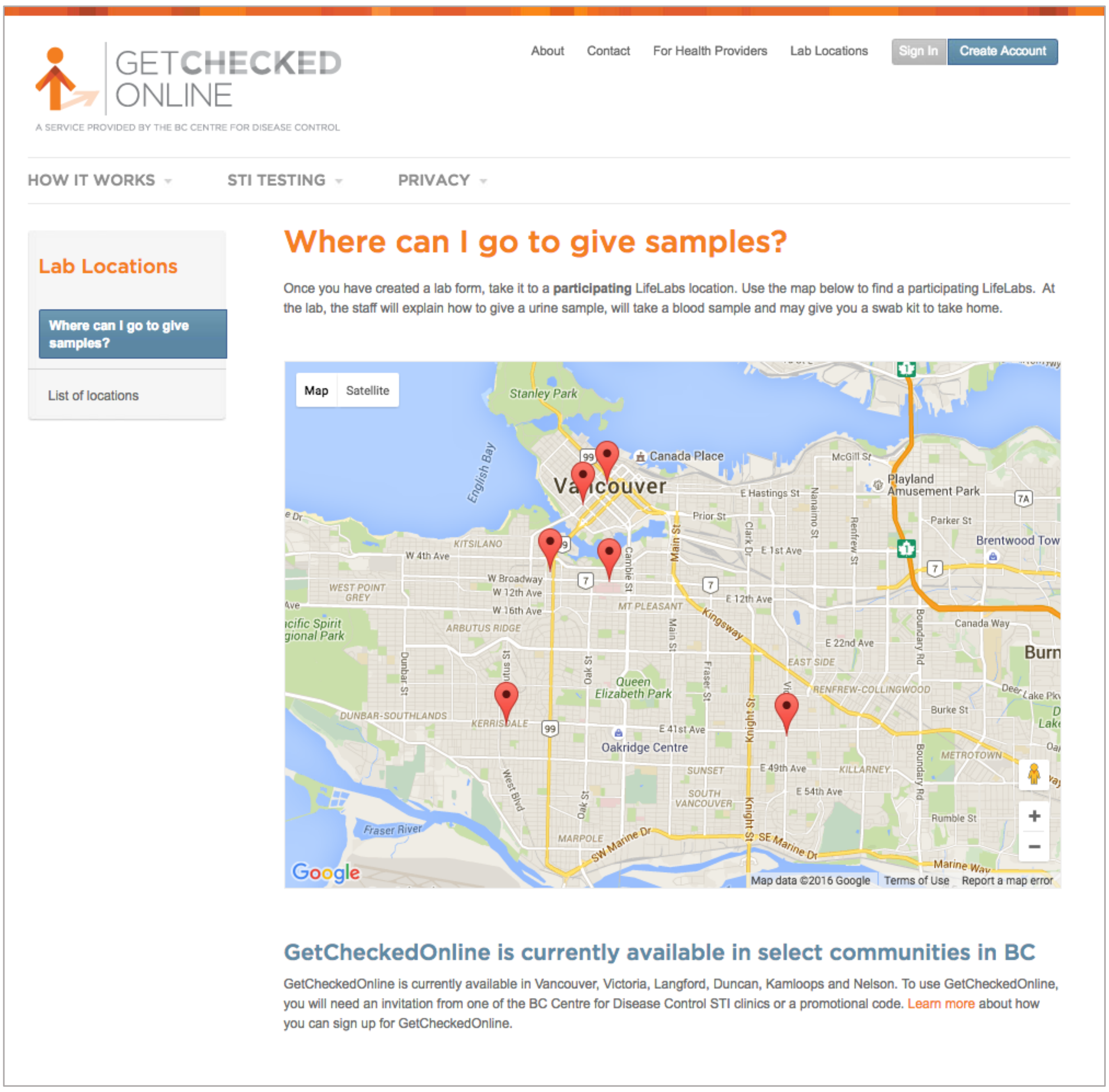

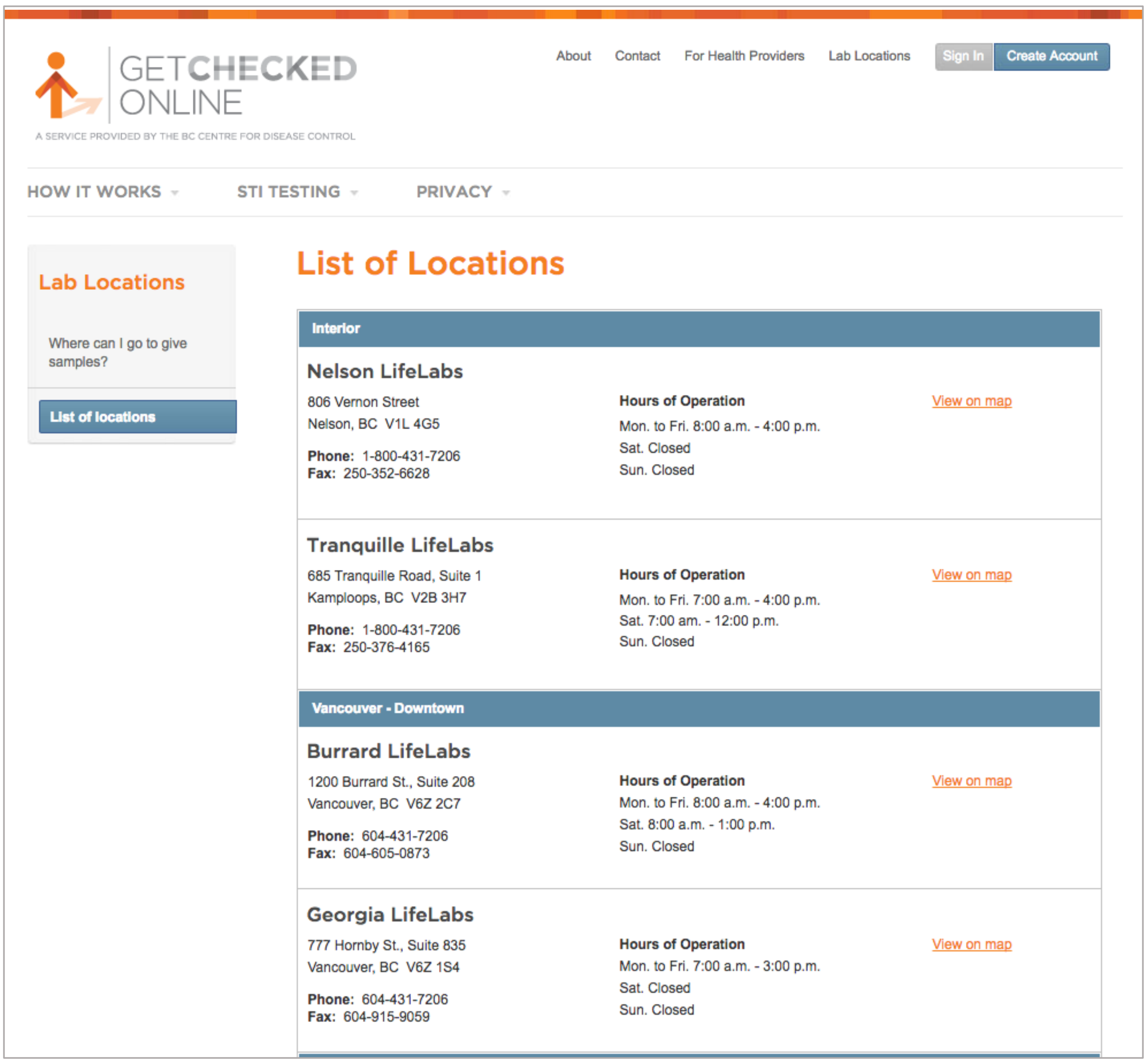

My Account

Clients can use the My Account pages to update their account details.

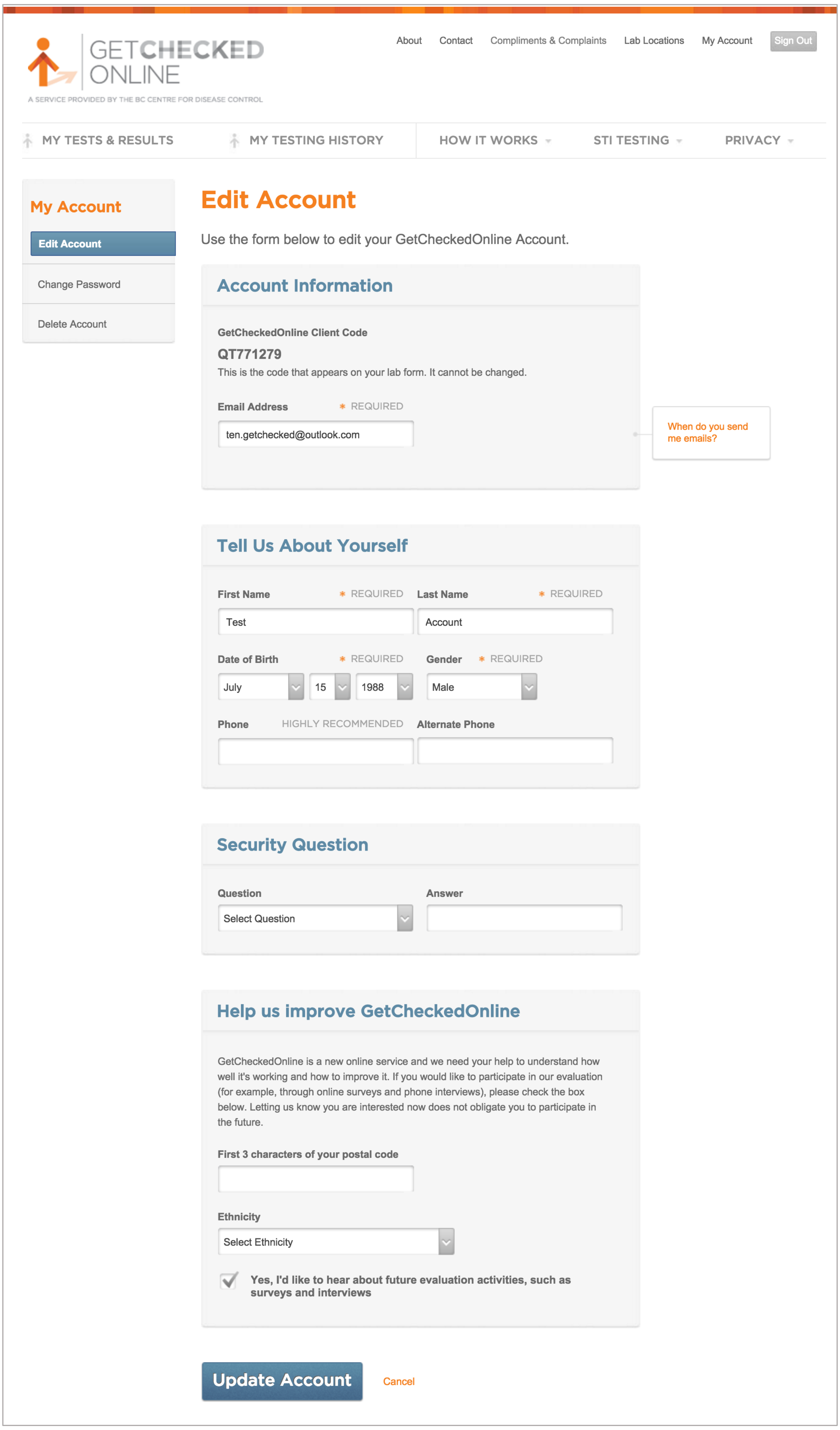

Contact Us

Clients can use this page to contact us for help or to give us feedback.

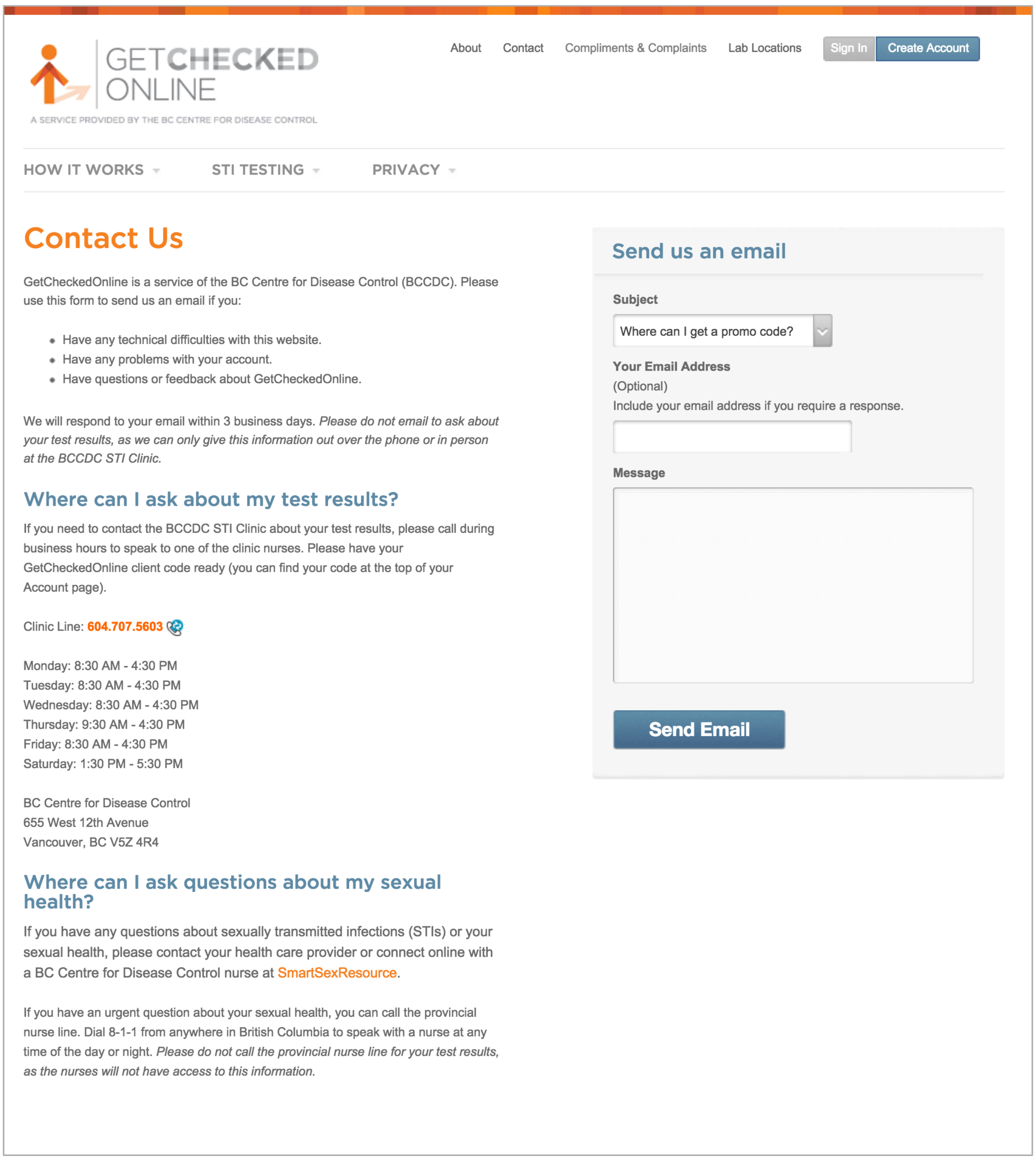

GETCHECKED ONLINE

A SERVICE PROVIDED BY THE BC CENTRE FOR DISEASE CONTROL

About Contact Compliments & Complaints Lab Locations Sign In Create Account

HOW IT WORKS STI TESTING PRIVACY

Contact Us

GetCheckedOnline is a service of the BC Centre for Disease Control (BCCDC). Please use this form to send us an email if you:

- Have any technical difficulties with this website.
- Have any problems with your account.
- Have questions or feedback about GetCheckedOnline.

We will respond to your email within 3 business days. *Please do not email to ask about your test results, as we can only give this information out over the phone or in person at the BCCDC STI Clinic.*

Where can I ask about my test results?

If you need to contact the BCCDC STI Clinic about your test results, please call during business hours to speak to one of the clinic nurses. Please have your GetCheckedOnline client code ready (you can find your code at the top of your Account page).

Clinic Line: **604.707.5603**

Monday: 8:30 AM - 4:30 PM
Tuesday: 8:30 AM - 4:30 PM
Wednesday: 8:30 AM - 4:30 PM
Thursday: 9:30 AM - 4:30 PM
Friday: 8:30 AM - 4:30 PM
Saturday: 1:30 PM - 5:30 PM

BC Centre for Disease Control
655 West 12th Avenue
Vancouver, BC V5Z 4R4

Where can I ask questions about my sexual health?

If you have any questions about sexually transmitted infections (STIs) or your sexual health, please contact your health care provider or connect online with a BC Centre for Disease Control nurse at SmartSexResource.

If you have an urgent question about your sexual health, you can call the provincial nurse line. Dial 8-1-1 from anywhere in British Columbia to speak with a nurse at any time of the day or night. *Please do not call the provincial nurse line for your test results, as the nurses will not have access to this information.*

Send us an email

Subject

Where can I get a promo code?

Your Email Address
(Optional)
Include your email address if you require a response.

Message

Send Email

Internal Pages: Patient Profile Search and Enter Test Results

The internal portion of the website is used to look-up client information, enter test results, get reports and administer the application. This section of the site is only accessible to internal users.

The Client Search screen is used to find clients. Tests results are entered on this page using the Enter Test Results lightbox.

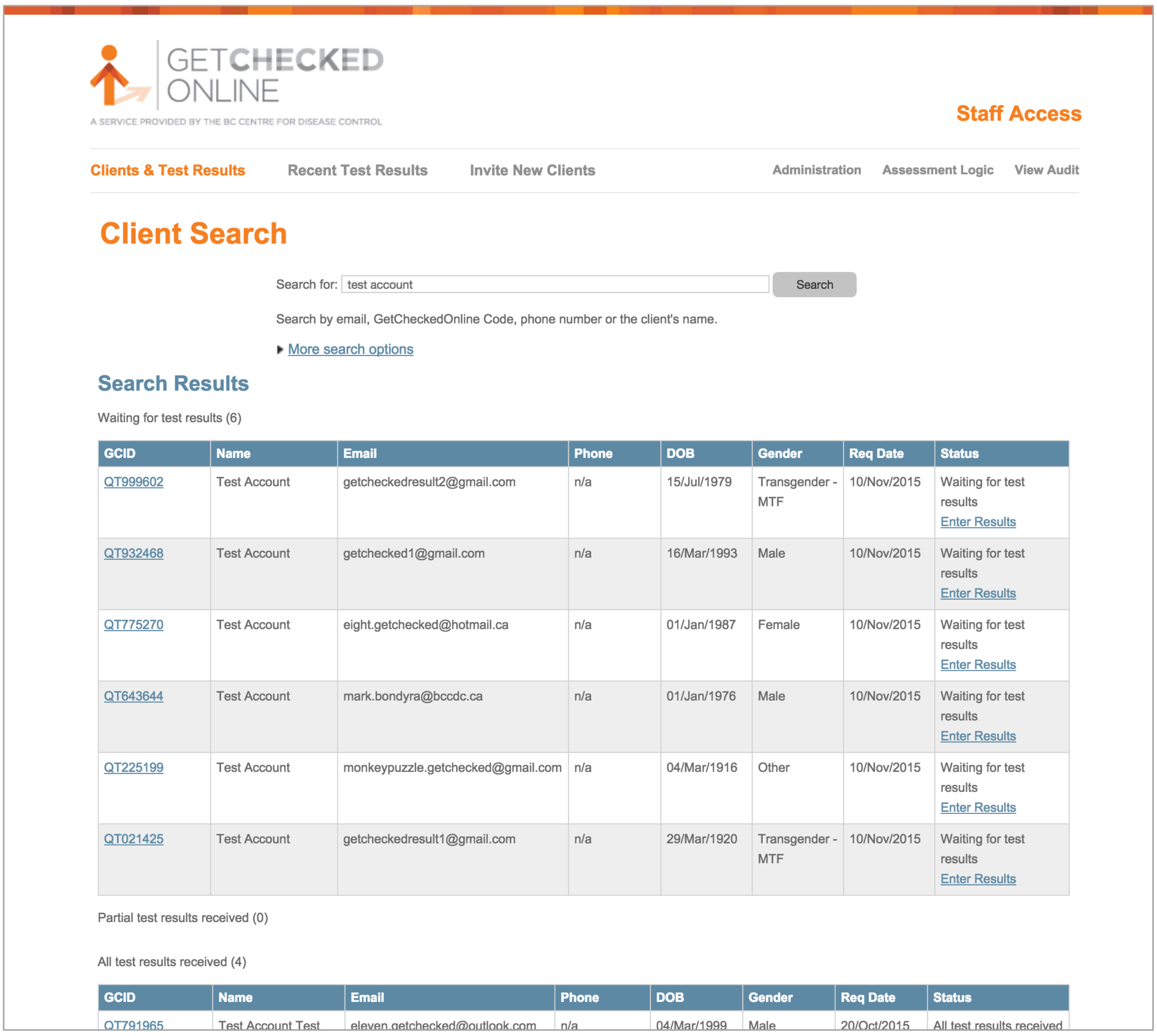

Enter Test Results lightbox:

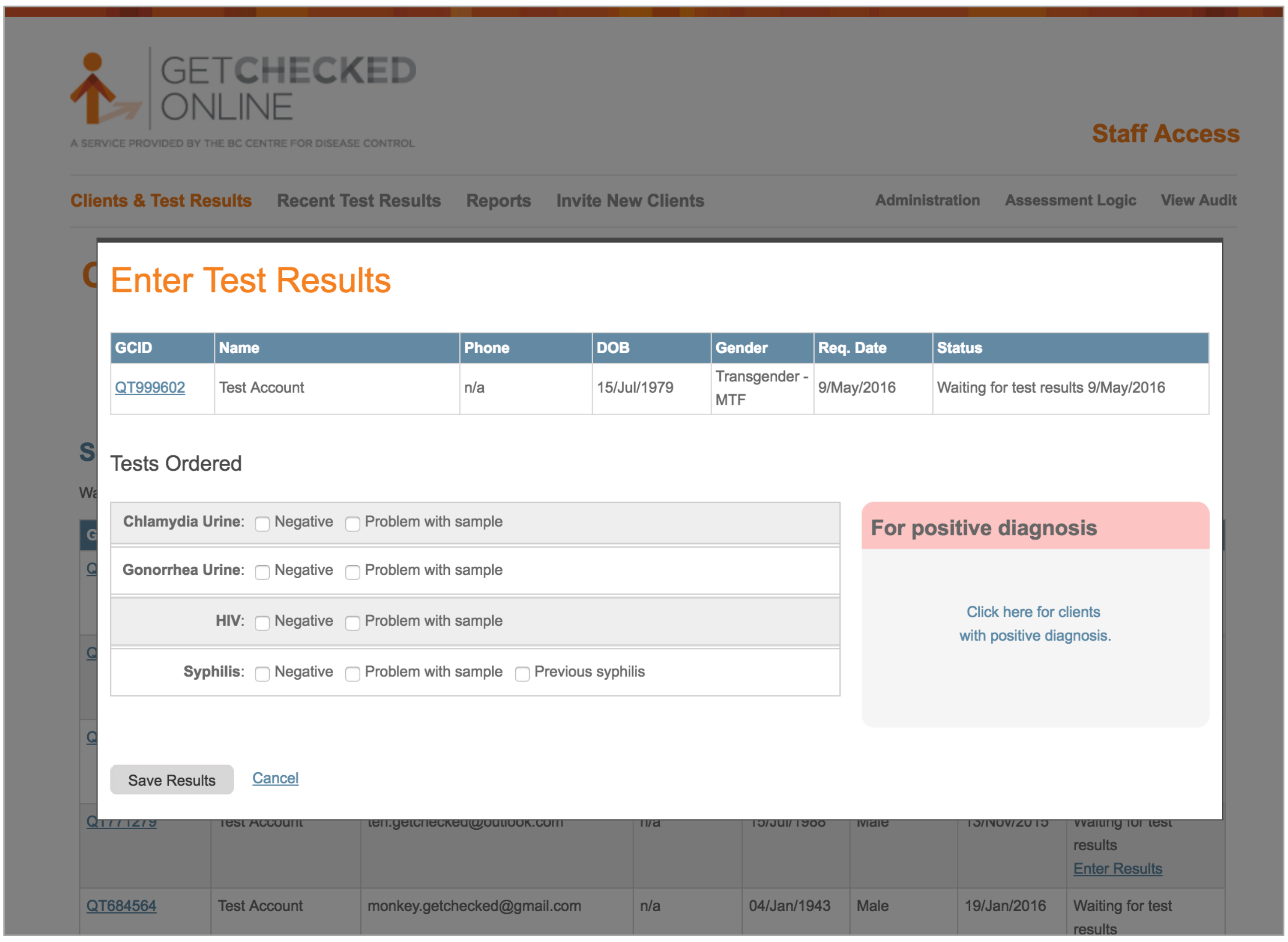

Internal Pages: Patient Profile

The patient profile screen shows more information about the patient including their testing history. Test results can also be entered from this page. A client's risk assessments can be viewed from this page as well.

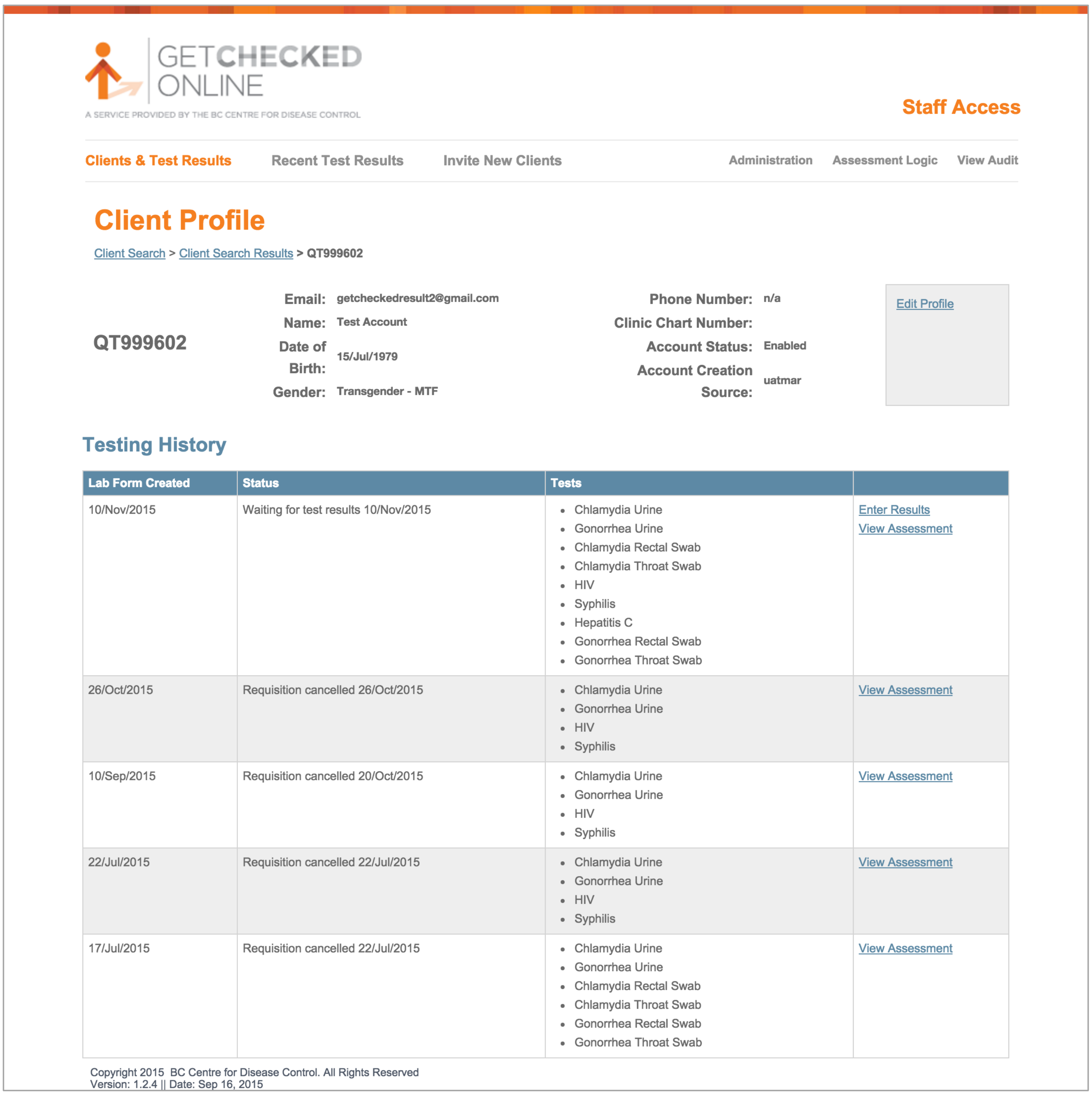

GETCHECKED ONLINE
A SERVICE PROVIDED BY THE BC CENTRE FOR DISEASE CONTROL

Staff Access

Clients & Test Results | Recent Test Results | Invite New Clients | Administration | Assessment Logic | View Audit

Client Profile

Client Search > Client Search Results > QT999602

QT999602

**Email:**	getcheckedresult2@gmail.com	**Phone Number:**	n/a
**Name:**	Test Account	**Clinic Chart Number:**	
**Date of Birth:**	15/Jul/1979	**Account Status:**	Enabled
**Gender:**	Transgender - MTF	**Account Creation Source:**	uatmar

Edit Profile

Testing History

Lab Form Created	Status	Tests	
10/Nov/2015	Waiting for test results 10/Nov/2015	• Chlamydia Urine<br>• Gonorrhea Urine<br>• Chlamydia Rectal Swab<br>• Chlamydia Throat Swab<br>• HIV<br>• Syphilis<br>• Hepatitis C<br>• Gonorrhea Rectal Swab<br>• Gonorrhea Throat Swab	Enter Results<br>View Assessment
26/Oct/2015	Requisition cancelled 26/Oct/2015	• Chlamydia Urine<br>• Gonorrhea Urine<br>• HIV<br>• Syphilis	View Assessment
10/Sep/2015	Requisition cancelled 20/Oct/2015	• Chlamydia Urine<br>• Gonorrhea Urine<br>• HIV<br>• Syphilis	View Assessment
22/Jul/2015	Requisition cancelled 22/Jul/2015	• Chlamydia Urine<br>• Gonorrhea Urine<br>• HIV<br>• Syphilis	View Assessment
17/Jul/2015	Requisition cancelled 22/Jul/2015	• Chlamydia Urine<br>• Gonorrhea Urine<br>• Chlamydia Rectal Swab<br>• Chlamydia Throat Swab<br>• Gonorrhea Rectal Swab<br>• Gonorrhea Throat Swab	View Assessment

Copyright 2015 BC Centre for Disease Control. All Rights Reserved
Version: 1.2.4 || Date: Sep 16, 2015

Internal Pages: Recent Test Results

The Recent Test Results Screen show tests results which were recently entered.

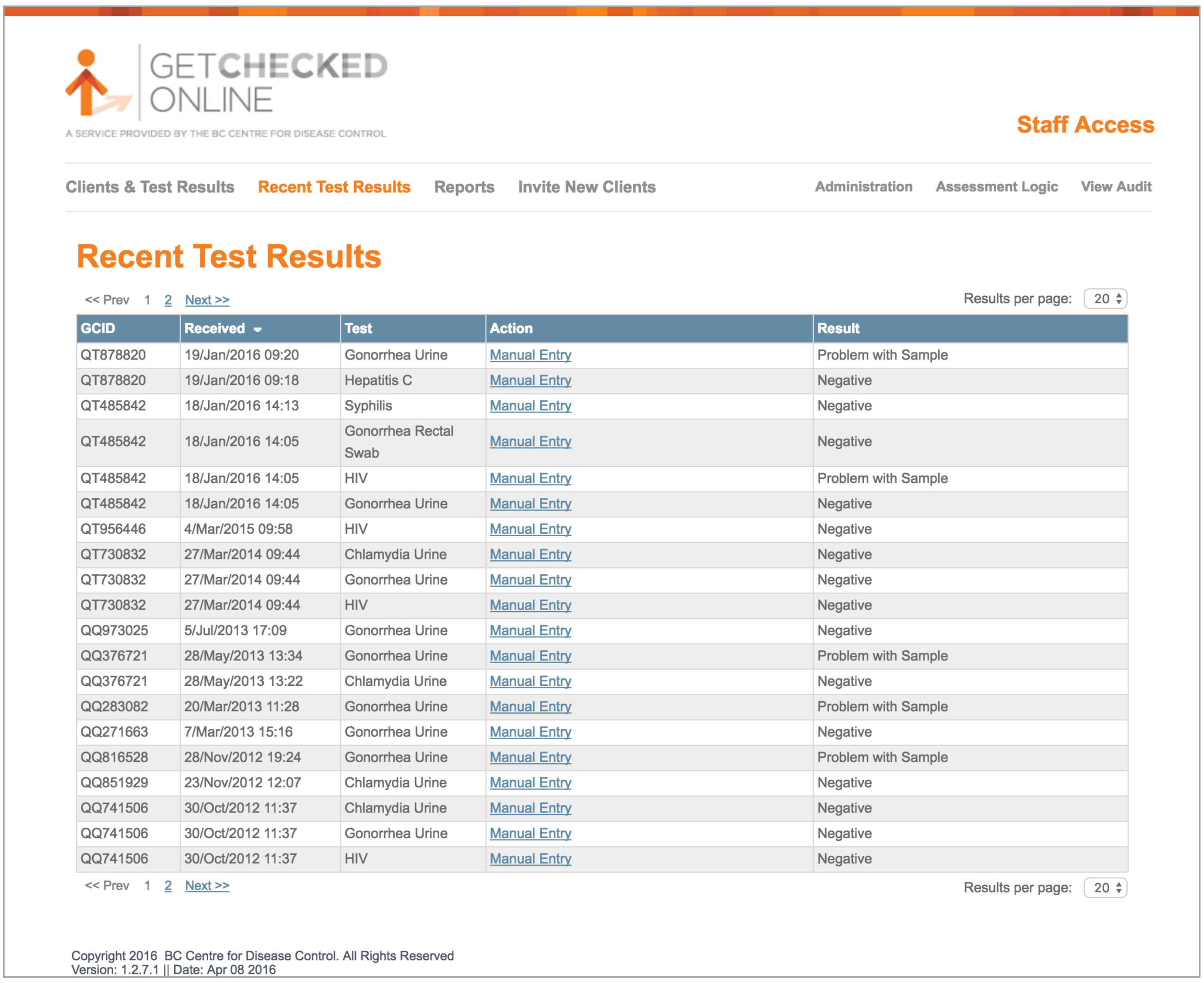

GETCHECKED ONLINE

A SERVICE PROVIDED BY THE BC CENTRE FOR DISEASE CONTROL

Staff Access

Clients & Test Results | Recent Test Results | Reports | Invite New Clients | Administration | Assessment Logic | View Audit

Recent Test Results

<< Prev 1 2 Next >> Results per page: 20

GCID	Received	Test	Action	Result
QT878820	19/Jan/2016 09:20	Gonorrhea Urine	Manual Entry	Problem with Sample
QT878820	19/Jan/2016 09:18	Hepatitis C	Manual Entry	Negative
QT485842	18/Jan/2016 14:13	Syphilis	Manual Entry	Negative
QT485842	18/Jan/2016 14:05	Gonorrhea Rectal Swab	Manual Entry	Negative
QT485842	18/Jan/2016 14:05	HIV	Manual Entry	Problem with Sample
QT485842	18/Jan/2016 14:05	Gonorrhea Urine	Manual Entry	Negative
QT956446	4/Mar/2015 09:58	HIV	Manual Entry	Negative
QT730832	27/Mar/2014 09:44	Chlamydia Urine	Manual Entry	Negative
QT730832	27/Mar/2014 09:44	Gonorrhea Urine	Manual Entry	Negative
QT730832	27/Mar/2014 09:44	HIV	Manual Entry	Negative
QQ973025	5/Jul/2013 17:09	Gonorrhea Urine	Manual Entry	Negative
QQ376721	28/May/2013 13:34	Gonorrhea Urine	Manual Entry	Problem with Sample
QQ376721	28/May/2013 13:22	Chlamydia Urine	Manual Entry	Negative
QQ283082	20/Mar/2013 11:28	Gonorrhea Urine	Manual Entry	Problem with Sample
QQ271663	7/Mar/2013 15:16	Gonorrhea Urine	Manual Entry	Negative
QQ816528	28/Nov/2012 19:24	Gonorrhea Urine	Manual Entry	Problem with Sample
QQ851929	23/Nov/2012 12:07	Chlamydia Urine	Manual Entry	Negative
QQ741506	30/Oct/2012 11:37	Chlamydia Urine	Manual Entry	Negative
QQ741506	30/Oct/2012 11:37	Gonorrhea Urine	Manual Entry	Negative
QQ741506	30/Oct/2012 11:37	HIV	Manual Entry	Negative

<< Prev 1 2 Next >> Results per page: 20

Copyright 2016 BC Centre for Disease Control. All Rights Reserved

Version: 1.2.7.1 || Date: Apr 08 2016

Internal Pages: Invite New Clients and Manage Promo Codes

The invite clients page is used to invite new clients to GetCheckedOnline. Clients can also create accounts using promo codes which are managed on the Manage Promo Codes screen.

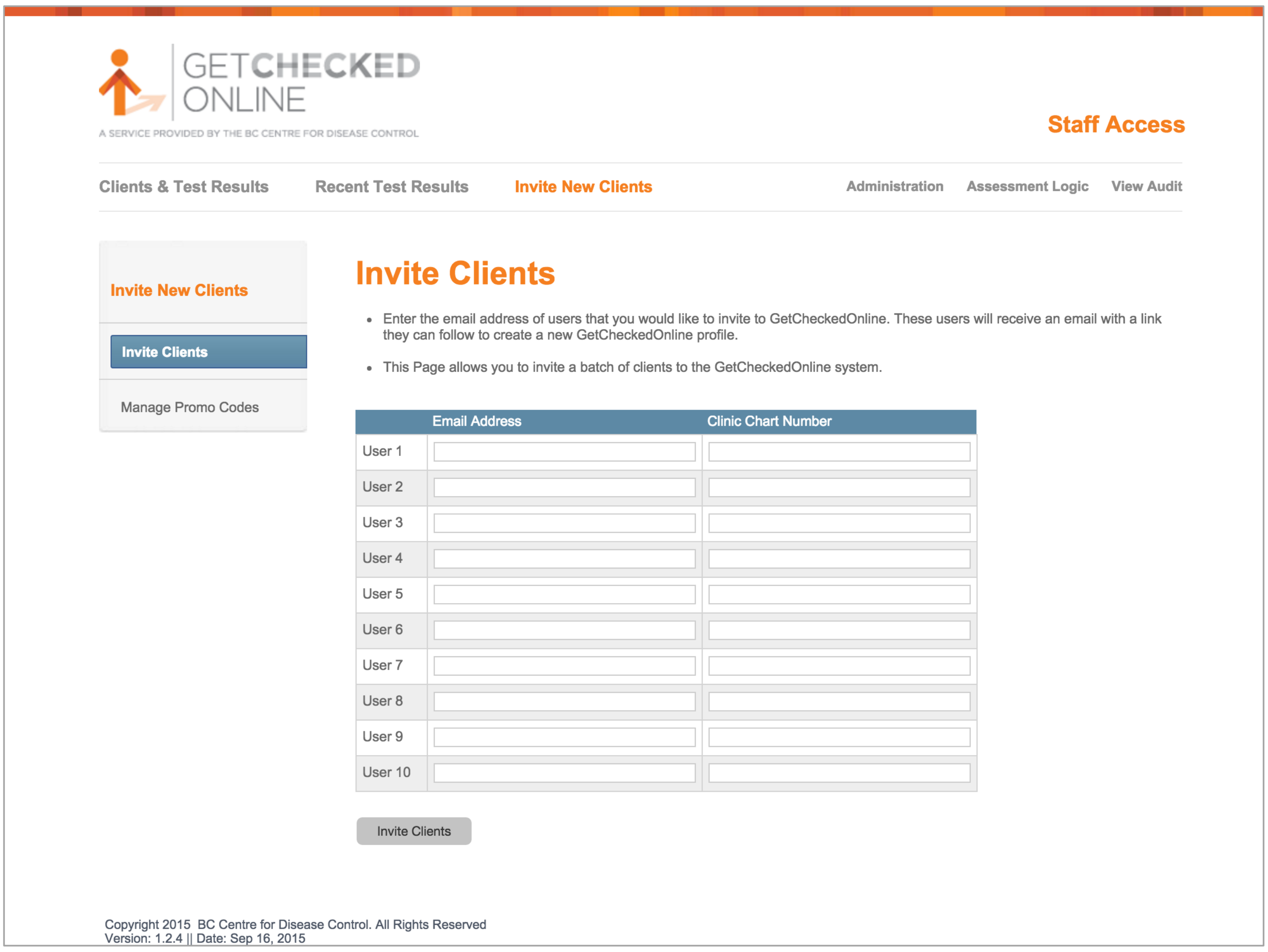

GETCHECKED ONLINE
A SERVICE PROVIDED BY THE BC CENTRE FOR DISEASE CONTROL

Staff Access

Clients & Test Results | Recent Test Results | Invite New Clients | Administration | Assessment Logic | View Audit

Invite New Clients
- Invite Clients
- Manage Promo Codes

Invite Clients

- Enter the email address of users that you would like to invite to GetCheckedOnline. These users will receive an email with a link they can follow to create a new GetCheckedOnline profile.
- This Page allows you to invite a batch of clients to the GetCheckedOnline system.

	Email Address	Clinic Chart Number
User 1		
User 2		
User 3		
User 4		
User 5		
User 6		
User 7		
User 8		
User 9		
User 10		

Invite Clients

Copyright 2015 BC Centre for Disease Control. All Rights Reserved
Version: 1.2.4 || Date: Sep 16, 2015

Manage Promo Codes:

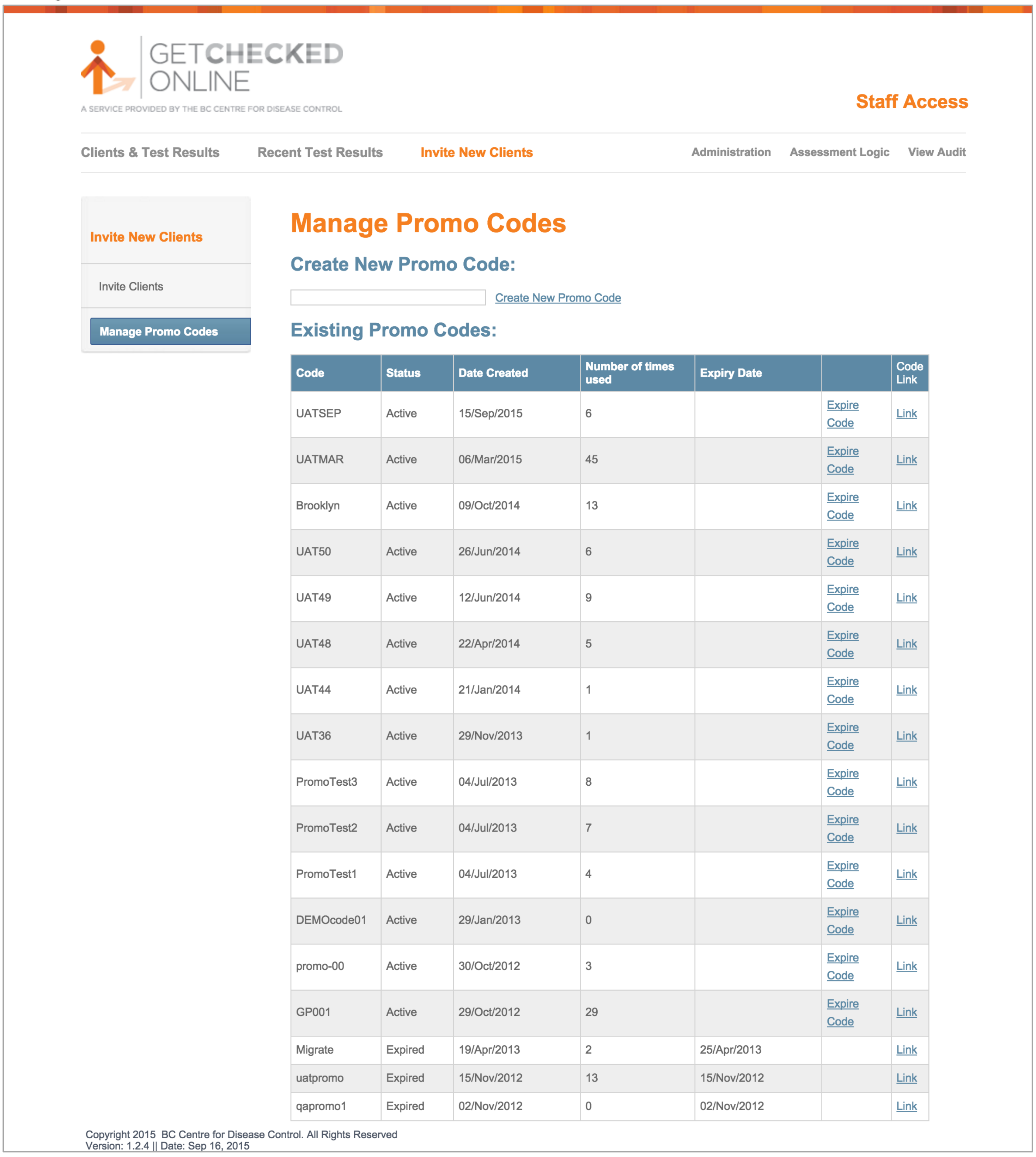

GETCHECKED ONLINE
A SERVICE PROVIDED BY THE BC CENTRE FOR DISEASE CONTROL

Staff Access

Clients & Test Results | Recent Test Results | Invite New Clients | Administration | Assessment Logic | View Audit

Invite New Clients
- Invite Clients
- Manage Promo Codes

Manage Promo Codes

Create New Promo Code:

Create New Promo Code

Existing Promo Codes:

Code	Status	Date Created	Number of times used	Expiry Date		Code Link
UATSEP	Active	15/Sep/2015	6		Expire Code	Link
UATMAR	Active	06/Mar/2015	45		Expire Code	Link
Brooklyn	Active	09/Oct/2014	13		Expire Code	Link
UAT50	Active	26/Jun/2014	6		Expire Code	Link
UAT49	Active	12/Jun/2014	9		Expire Code	Link
UAT48	Active	22/Apr/2014	5		Expire Code	Link
UAT44	Active	21/Jan/2014	1		Expire Code	Link
UAT36	Active	29/Nov/2013	1		Expire Code	Link
PromoTest3	Active	04/Jul/2013	8		Expire Code	Link
PromoTest2	Active	04/Jul/2013	7		Expire Code	Link
PromoTest1	Active	04/Jul/2013	4		Expire Code	Link
DEMOcode01	Active	29/Jan/2013	0		Expire Code	Link
promo-00	Active	30/Oct/2012	3		Expire Code	Link
GP001	Active	29/Oct/2012	29		Expire Code	Link
Migrate	Expired	19/Apr/2013	2	25/Apr/2013		Link
uatpromo	Expired	15/Nov/2012	13	15/Nov/2012		Link
qapromo1	Expired	02/Nov/2012	0	02/Nov/2012		Link

Copyright 2015 BC Centre for Disease Control. All Rights Reserved
Version: 1.2.4 || Date: Sep 16, 2015

Internal Pages: Reports

The reports section contains two reports, one showing clients with partial test results and the other showing frequent testers.

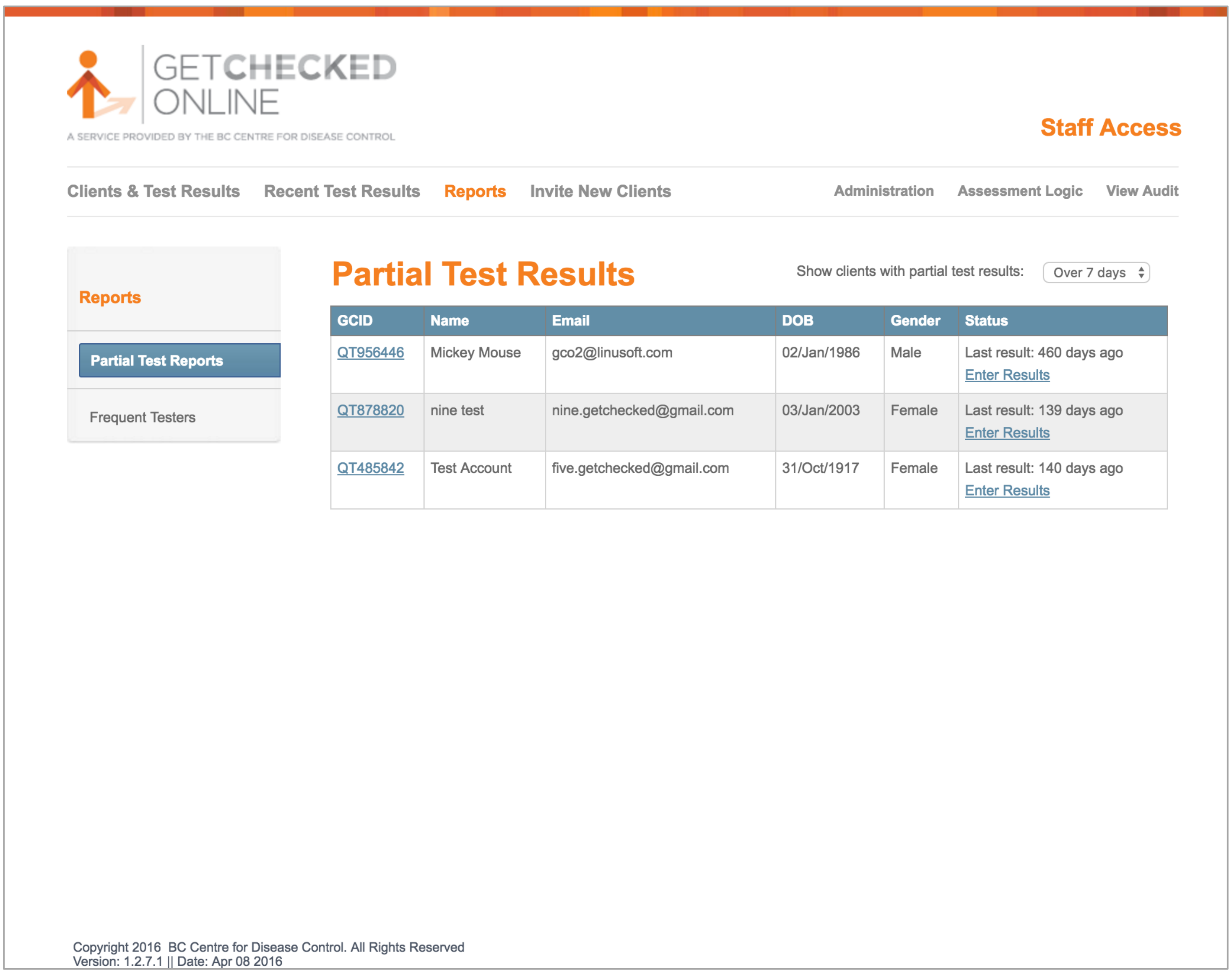

Internal Pages: Administration

The Administration section of the internal site is use to manage the configuration and some of the content of the application.

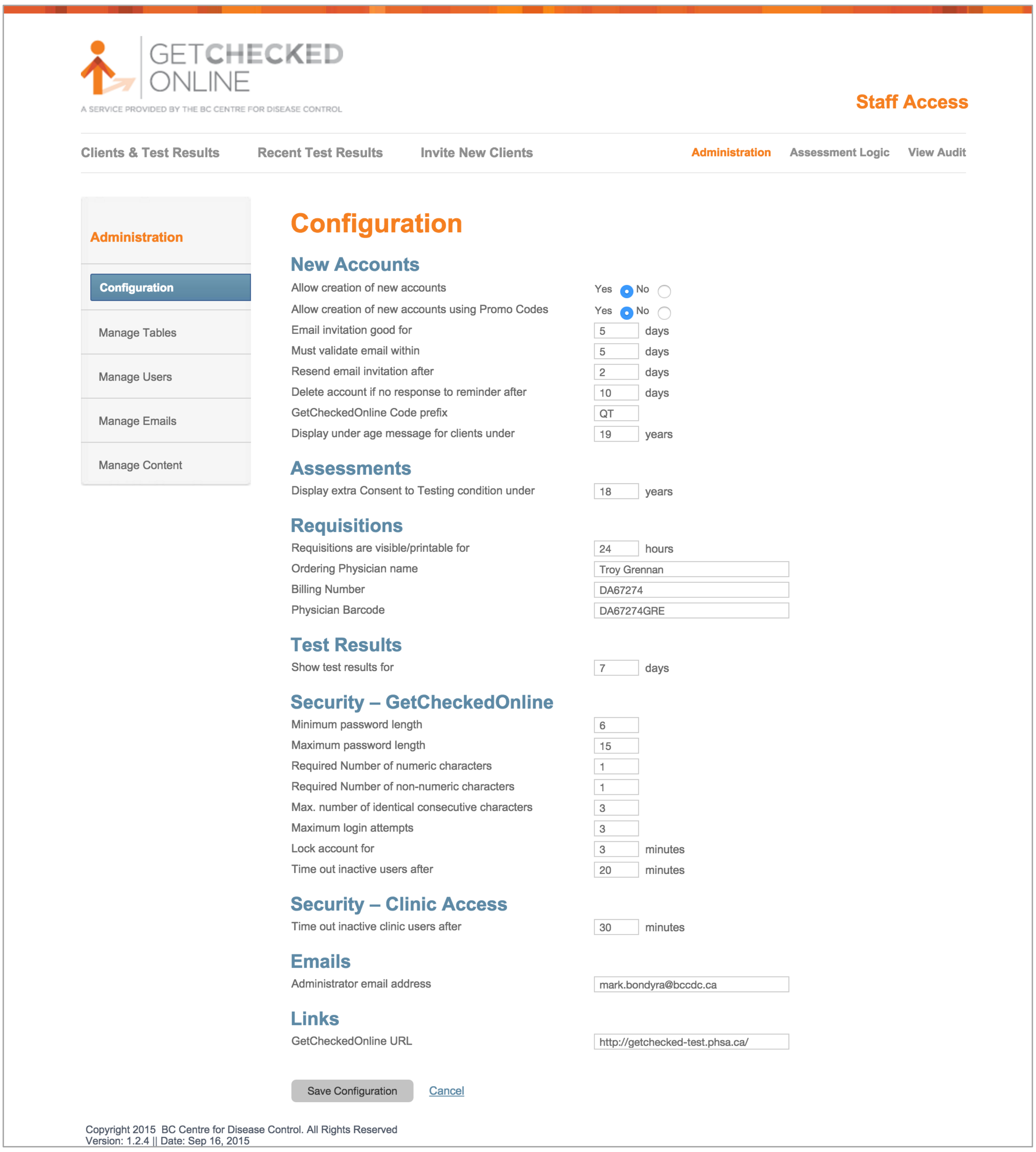

Internal Pages: Assessment Logic

This section is used to administer the assessment questions, triage screen, educational messages, tests and recommended testing frequencies.

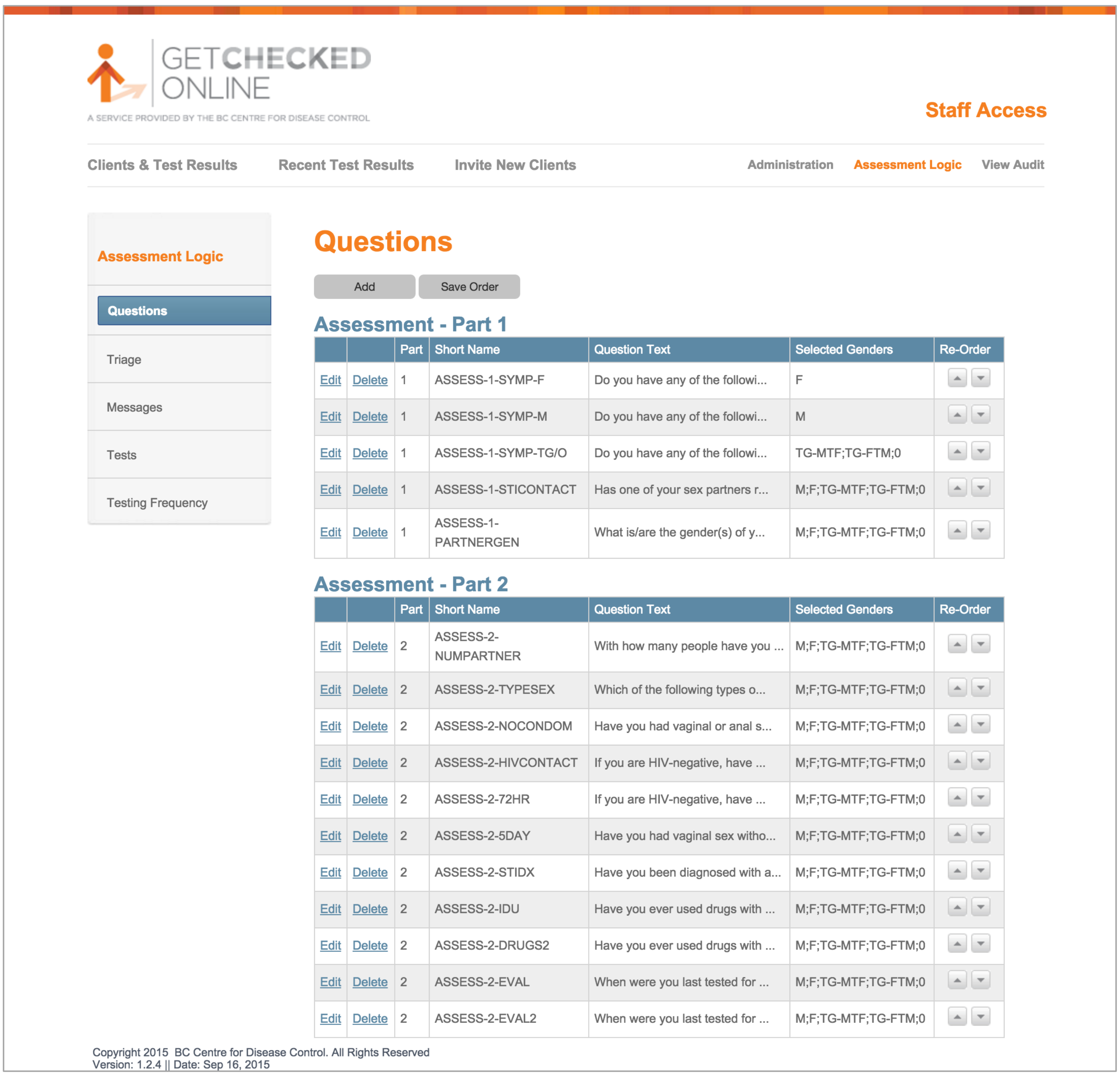

Assessment - Part 1

		Part	Short Name	Question Text	Selected Genders	Re-Order
Edit	Delete	1	ASSESS-1-SYMP-F	Do you have any of the followi...	F	
Edit	Delete	1	ASSESS-1-SYMP-M	Do you have any of the followi...	M	
Edit	Delete	1	ASSESS-1-SYMP-TG/O	Do you have any of the followi...	TG-MTF;TG-FTM;0	
Edit	Delete	1	ASSESS-1-STICONTACT	Has one of your sex partners r...	M;F;TG-MTF;TG-FTM;0	
Edit	Delete	1	ASSESS-1-PARTNERGEN	What is/are the gender(s) of y...	M;F;TG-MTF;TG-FTM;0	

Assessment - Part 2

		Part	Short Name	Question Text	Selected Genders	Re-Order
Edit	Delete	2	ASSESS-2-NUMPARTNER	With how many people have you ...	M;F;TG-MTF;TG-FTM;0	
Edit	Delete	2	ASSESS-2-TYPESEX	Which of the following types o...	M;F;TG-MTF;TG-FTM;0	
Edit	Delete	2	ASSESS-2-NOCONDOM	Have you had vaginal or anal s...	M;F;TG-MTF;TG-FTM;0	
Edit	Delete	2	ASSESS-2-HIVCONTACT	If you are HIV-negative, have ...	M;F;TG-MTF;TG-FTM;0	
Edit	Delete	2	ASSESS-2-72HR	If you are HIV-negative, have ...	M;F;TG-MTF;TG-FTM;0	
Edit	Delete	2	ASSESS-2-5DAY	Have you had vaginal sex witho...	M;F;TG-MTF;TG-FTM;0	
Edit	Delete	2	ASSESS-2-STIDX	Have you been diagnosed with a...	M;F;TG-MTF;TG-FTM;0	
Edit	Delete	2	ASSESS-2-IDU	Have you ever used drugs with ...	M;F;TG-MTF;TG-FTM;0	
Edit	Delete	2	ASSESS-2-DRUGS2	Have you ever used drugs with ...	M;F;TG-MTF;TG-FTM;0	
Edit	Delete	2	ASSESS-2-EVAL	When were you last tested for ...	M;F;TG-MTF;TG-FTM;0	
Edit	Delete	2	ASSESS-2-EVAL2	When were you last tested for ...	M;F;TG-MTF;TG-FTM;0	

Copyright 2015 BC Centre for Disease Control. All Rights Reserved
Version: 1.2.4 || Date: Sep 16, 2015

Internal Pages: Audit

The audit section give access to the system audit functionality.

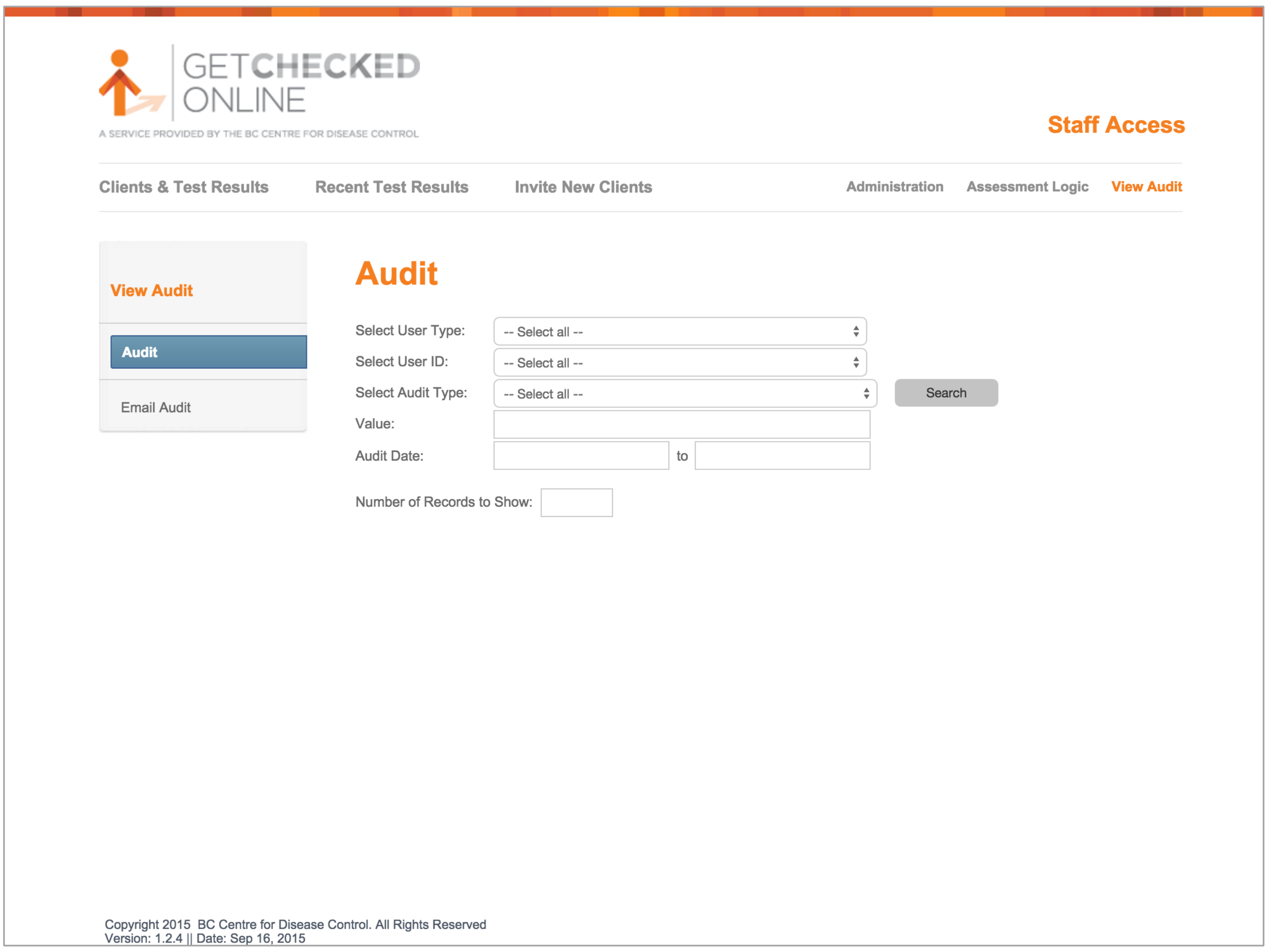

Supplement: Multimedia Appendix 1 [file resprot_v5i3e186_app1.pdf]
