# Supplementary material for: Get Checked… Where? The Development of a Comprehensive, Integrated Internet-Based Testing Program for Sexually Transmitted and Blood-Borne Infections in British Columbia, Canada
Source: JMIR Res Protoc. 2016 Sep 20;5(3):e186. doi: 10.2196/resprot.6293 (PMC5050385; doi:10.2196/resprot.6293)
Supplement: Multimedia Appendix 3 [file resprot_v5i3e186_app3.pdf]

Rectal Swab

Please read all instructions before taking your sample.

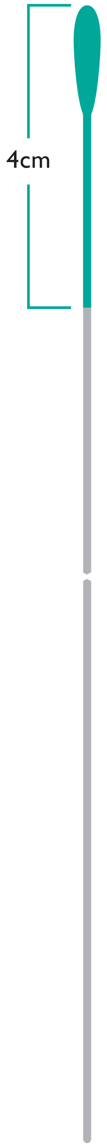

1

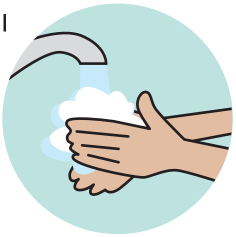

Wash your hands with soap and water.

2

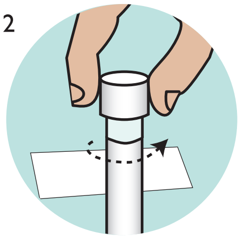

Spread a paper towel down on a flat surface.

Twist the cap off the tube that has **R** on the **green** sticker.

3

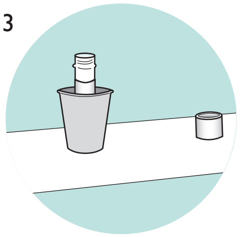

Put the cap foil side down on the paper towel.

Put the tube upright in a cup or glass.

4

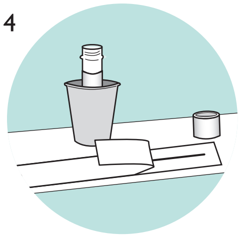

Partially open the swab package. Don't touch the soft tip of the swab with your hands or other surfaces.

5

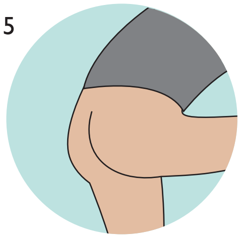

Get into a position that gives you the easiest access to your anus.

6

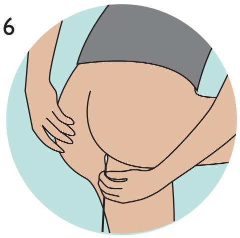

Take one hand and spread one bum cheek to the side. With your other hand, put the swab at the opening of your anus.

7

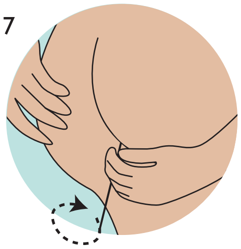

Insert the swab about 4 cm.

Gently move the swab in a circle 2 or 3 times, touching the walls of the rectum.

8

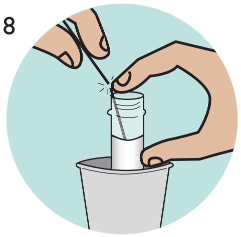

Take the swab out and put it in the tube.

Break the swab at the score line.

9

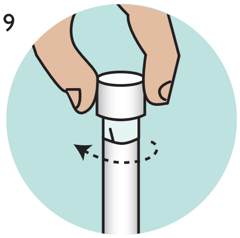

Put the cap back on the tube tightly.

10

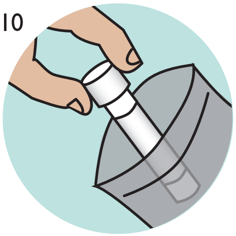

Put the tube in the plastic "Biohazard" bag that also contains the paper lab form. Close the bag.

11

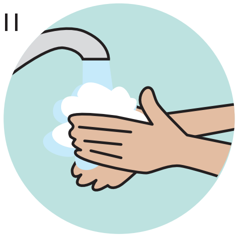

Wash your hands with soap and water.

12

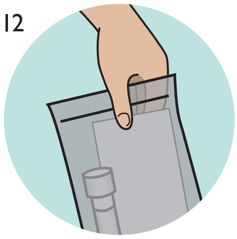

Within 10 days, bring the plastic bag with your swab(s) and lab form back to the LifeLabs location where you picked up the swab kit.

Worried you're not doing this correctly?

Research shows that collecting your own swab is just as effective as having it done by a nurse or doctor.

Your results will be ready in 7-12 days.

You will receive an email from GetCheckedOnline letting you know when your results are ready.

GETCHECKED ONLINE

BC Centre for Disease Control
An agency of the Provincial Health Services Authority

© 2016 BC Centre for Disease Control

Throat Swab

Take this swab first

Please read all instructions before taking your sample.

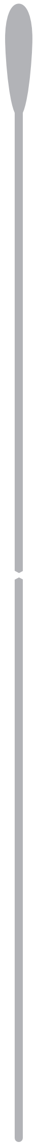

1

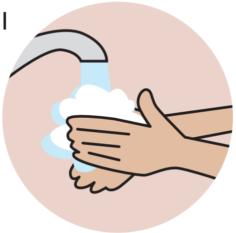

Wash your hands with soap and water.

2

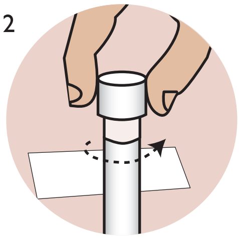

Spread a paper towel down on a flat surface.

Twist the cap off the tube that has **T** on the **red** sticker.

3

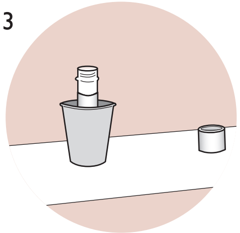

Put the cap foil side down on the paper towel.

Put the tube upright in a cup or glass.

4

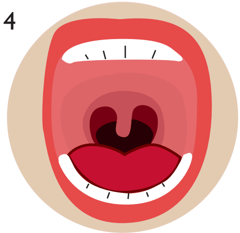

Take the swab out of the package. Open your mouth as wide as you can, take a deep breath and hold it.

5

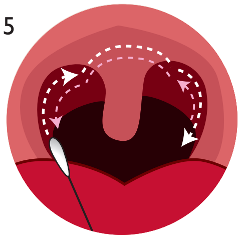

Trying not to touch your tongue, put the swab to the very back of your throat (it's OK if you accidentally touch your tongue).

Rub the tip firmly and quickly from one side of your throat to the other and back.

It's OK if you gag.

6

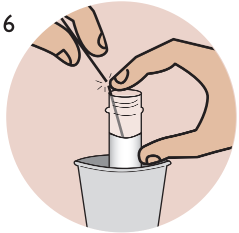

Take the swab out and put it in the tube.

Break the swab at the score line.

7

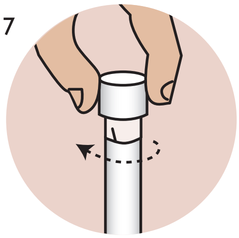

Put the cap back on the tube tightly.

8

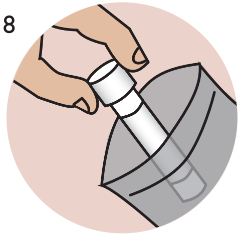

Put the tube in the plastic "Biohazard" bag that also contains the paper lab form. Close the bag.

9

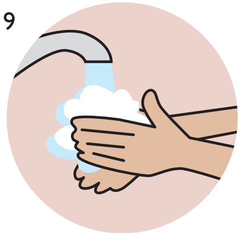

Wash your hands with soap and water.

10

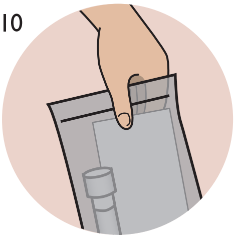

Within 10 days, bring the plastic bag with your swab(s) and lab form back to the LifeLabs location where you picked up the swab kit.

Worried you're not doing this correctly?

Research shows that collecting your own swab is just as effective as having it done by a nurse or doctor.

Your results will be ready in 7-12 days.

You will receive an email from GetCheckedOnline letting you know when your results are ready.

GETCHECKED ONLINE

BC Centre for Disease Control
An agency of the Provincial Health Services Authority

© 2016 BC Centre for Disease Control
